# Tacrine-Based Hybrids: Past, Present, and Future

**DOI:** 10.3390/ijms24021717

**Published:** 2023-01-15

**Authors:** Anna Bubley, Alexaner Erofeev, Peter Gorelkin, Elena Beloglazkina, Alexander Majouga, Olga Krasnovskaya

**Affiliations:** 1 Chemistry Department, Lomonosov Moscow State University, Leninskie Gory 1-3, Moscow 119991, Russia; 2 Department of Materials Science of Semiconductors and Dielectrics, National University of Science and Technology (MISIS), Leninskiy Prospect 4, Moscow 119049, Russia

**Keywords:** Alzheimer, acetylcholinesterase, butyrylcholinesterase, amyloid-β, tacrine

## Abstract

Alzheimer’s disease (AD) is a neurodegenerative disorder which is characterized by β-amyloid (Aβ) aggregation, τ-hyperphosphorylation, and loss of cholinergic neurons. The other important hallmarks of AD are oxidative stress, metal dyshomeostasis, inflammation, and cell cycle dysregulation. Multiple therapeutic targets may be proposed for the development of anti-AD drugs, and the “one drug–multiple targets” strategy is of current interest. Tacrine (THA) was the first clinically approved cholinesterase (ChE) inhibitor, which was withdrawn due to high hepatotoxicity. However, its high potency in ChE inhibition, low molecular weight, and simple structure make THA a promising scaffold for developing multi-target agents. In this review, we summarized THA-based hybrids published from 2006 to 2022, thus providing an overview of strategies that have been used in drug design and approaches that have resulted in significant cognitive improvements and reduced hepatotoxicity.

## 1. Introduction

AD is a progressive multifarious neurodegenerative disorder which is described by a progressive loss of cognitive abilities, such as memory, language skills, and attention, as well as by spatial disorientation and depression. The pathological hallmarks of AD are extracellular accumulation of Aβ plaques composed of Aβ peptides, neurofibrillary tangles (NFTs) composed of hyperphosphorylated tau protein, brain inflammation, and atrophy [1]. Aβ is formed from amyloid precursor protein (APP), which is cleaved by β-secretase (BACE-1) and γ-secretase. Thus, interfering with fibril formation, including metal cation chelation, the disruption of amyloid aggregation, and BACE-1 inhibition, are well-established approaches to the development of anti-AD drugs [2].

Oxidative stress and inflammation are also some of the hallmarks of AD [3]. An increase in reactive oxygen species (ROS) levels is caused by mitochondrial dysfunction, violation of the homeostasis of metal cations, the formation of Aβ fibrils, inflammatory processes, etc. [4].

Calcium regulation is important in learning and memory. The disruption of Ca^2+^ level homeostasis caused by the formation of Aβ leads to cell death [5,6]. Blocking Ca^2+^ channels is also one of the important strategies in the treatment of AD. Calcium channel blockers (CCBs), one of the more commonly used treatments for hypertension, are also considered as potential drug candidates for anti-AD therapy [7].

Glycogen synthase kinase-3 (GSK-3) is a serine-threonine kinase involved in neurodegeneration. GSK-3β isoform is found to be hyperactive in the brains of AD patients; GSK-3β inhibition is also one of the therapeutic strategies during anti-AD drug development [8,9].

Neurotransmitters such as noradrenaline, dopamine, serotonin (5-HT), and GABA are involved in the pathogenesis of AD; imbalances between neurotransmitters in the temporal cortex and hippocampus have been reported [10]. In addition, in the latter studies of AD, a deficiency of monoamines is found in the brainstem and hippocampus [11]. NMDA antagonists slow the decline in cognitive function in AD patients [3,12].

Different components of the cholinergic system are therapeutic targets in AD treatment. ACh is synthesized from acetyl coenzyme A and choline in the presence of choline acetyltransferase (ChAT). Then, ACh is released into the synapse and binds to either the G-protein coupled muscarinic receptors or the ionotropic nicotinic receptors to transmit signals from one neuron to the other [13]. ACh can be degraded by AChE or BuChE [14]. The activation of the muscarinic M1 receptor exerts a pro-cognitive effect, and an activation of the alpha7 nACh receptor might inhibit the formation of Aβ [15]. Thus, drugs capable of acting on muscarinic and nicotinic receptors are of interest [16,17]. Moreover, the mAChR antagonist scopolamine is used for inducing cognitive and behavioral deficits in animals [18].

Current clinical therapy for AD patients is based on the cholinergic hypothesis, which suggests that the decline of acetylcholine (ACh) levels causes cognitive and memory deficits [19]. An increase in the ACh concentration in a synaptic cleft by various ways, such as the inhibition of both acetylcholinesterase (AChE) and butyrylcholinesterase (BuChE), is the key approach in the treatment of AD now. AChE inhibitors (AChEIs), including donepezil, galantamine, and rivastigmine, are FDA-approved drugs for AD treatment [20].

Tacrine (9-amine-1,2,3,4-tetrahydroacridine) (THA) was the first FDA-approved ChE inhibitor for the treatment of AD. THA was produced under the brand name Cognex^®^ and the recommended dose was 40 mg per day. THA acts by inhibiting the metabolism of acetylcholine and thus prolonging its activity and raising levels in the cerebral cortex. Therapy with THA improves mental functioning in patients with mild-to-moderate dementia of Alzheimer disease [21,22]. THA undergoes first pass metabolism by the liver and is extensively metabolized by the cytochrome P450 system, which is supposedly the reason for its high hepatotoxicity. Thus, THA therapy is accompanied by increased serum alanine aminotransferase (ALT) and aspartate aminotransferase (ASAT) levels, which are indicative of liver damage and which were the reason for its withdrawal from use in 2013 [22].

**Figure 1:**
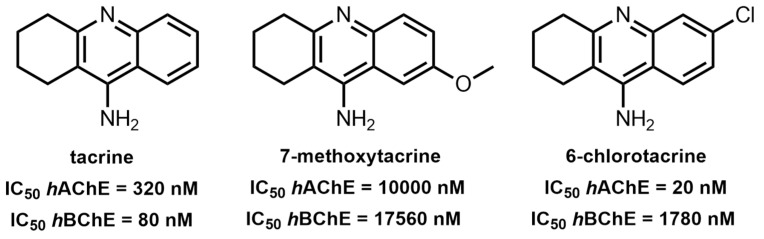
Tacrine (THA), 7-metoxytacrine (7-MEOTA), 6-chlorotacrine (6-Cl-THA), and their IC_50_ values.

Intensive research resulted in the design of more potent 6-chlorotacrine (6-Cl-THA) and less toxic 7-methoxytacrine (7-MEOTA) drug candidates [23,24,25] (Figure 1).

Despite the hepatotoxicity of THA, its suitability for chemical modification makes it a widely used scaffold for drug development [26]. Chemical modification of amino groups in the THA molecule leads to a decrease in hepatotoxicity [27]. Thus, a conjugation of THA with a second pharmacophoric moiety resulting in THA-based hybrids, pioneered by Pang et al. [28], is still an area of active research and development.

Since the strategy of ChE inhibition was successful, much attention is paid to the development of drugs that effectively interact with this enzyme. The crystallographic structure of AChE reveals that it has a narrow 20A gorge with two binding sites, the catalytic active site (CAS) at the bottom and the peripheral anionic site (PAS) near the entrance [29,30]. AChE inhibitors can bind to either one or two sites. Importantly, AChE could also promote Aβ formation by interaction through the PAS of AChE, yielding the toxic AChE-Aβ complex [31]. Therefore, the dual binding inhibitors, which target both PAS and CAS, are of interest in AD treatment, and THA-linker-residue hybrids with appropriate linker length are being designed [32].

Based on the above-mentioned multiple cellular and pathological hallmarks of AD, several therapeutic strategies should be used in developing effective anti-AD therapy, and a potential drug candidate should affect several therapeutic targets at once to be effective. Thus, multi-target-directed THA-based hybrids have been of interest for years, and novel potential anti-AD THA-based drugs are still reported. Several reports have considered designing THA-like compounds by replacing or annulating the benzene ring in THA with different heterocyclic systems [33,34] and THA dimers [35]. Additionally, several reviews have reported THA-based hybrids. In 2017, Sameem et al. reported a short review of THA-based scaffolds as multi-target drugs (MTDLs) [36] and Wu et al. reported a review of several THA-based hybrids [27]. In 2019, Girek et al. summarized phyto-THA hybrids [37]. In addition, in 2020, Eckroat et al. summarized structural analogues of THA developed in 2015–2020 [38].

**Figure 2:**
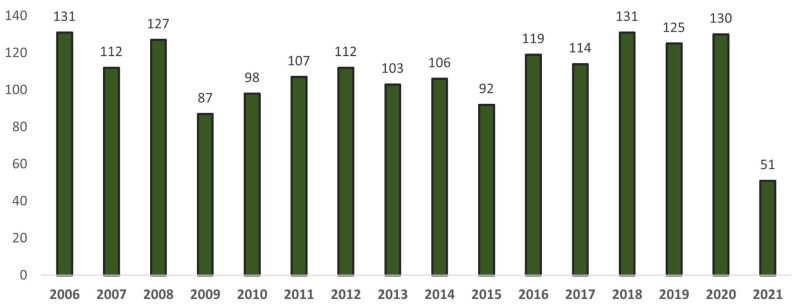
Result of a quick search of articles devoted to THA vs. year. Librarysearch.library.utoronto.ca. (accessed on 18 August 2022).

The development of new multi-target drugs based on THA and its analogues is a relevant task, and novel high-quality research works continue to be published. A quick search of the database of articles on THA and its analogues shows that the development of drugs against AD based on THA is of current interest (Figure 2).

In this review, we sum up THA-linker-residue hybrids published from 2006 to 2022, with many of them being superior to currently clinically used drugs in terms of their multiplicity of biological action, low toxicity, and drug efficacy.

## 2. Summary of Tacrine-Based Hybrids Reported in 2006–2022

**Figure 3:**
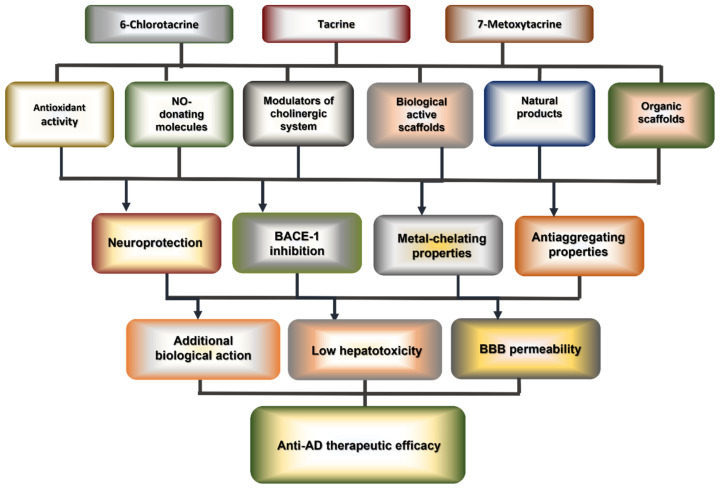
THA-based hybrids with various biological activities, summarized in this review.

Because the number of articles devoted to the development of analogues of THA is enormous, we structured the articles published in 2006–2022 [30,39,40,41,42,43,44,45,46,47,48,49,50,51,52,53,54,55,56,57,58,59,60,61,62,63,64,65,66,67,68,69,70,71,72,73,74,75,76,77,78,79,80,81,82,83,84,85,86,87,88,89,90,91,92,93,94,95,96,97,98,99,100,101,102,103,104,105,106,107,108,109,110,111,112,113,114,115,116,117,118,119,120,121,122,123,124,125,126,127,128,129,130,131,132,133] based on general patterns. This review is divided into several sections based on the biological action of the second ligand conjugated to the THA backbone (Figure 3, Table S1).

**Figure 4:**
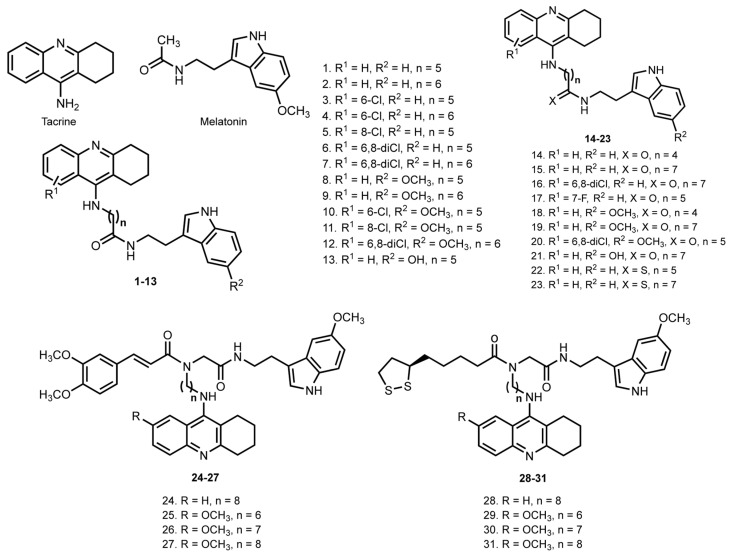
THA–melatonin hybrids **1**–**13** [39], an extended series of THA (6-Cl-THA)–melatonin hybrids **14**–**23** [40], and THA–melatonin hybrids with ferulic acid or lipoic acid **24**–**31** [41].

Tacrine hybrids with antioxidant activity (Section 3), NO-donors (Section 4), with biologically active molecules (Section 5), and with drugs that affect the cholinergic/serotonergic systems (Section 6) are summarized in various chapters. Additionally, two chapters are devoted to tacrine hybrids with natural products (Section 7) and organic ligands (Section 8). Below, the plan of this review is presented in the form of a diagram (Figure 4). In addition, in Table S1, we summarize all hybrids described in this review, present the best result of inhibitory activity among the series, and the spectrum of biological actions confirmed for hybrids.

Additionally, we summarize the in vivo therapeutic efficacy of hybrids data presented in this review in Table S2.

## 3. Tacrine Hybrids with Antioxidant Activity

### 3.1. Tacrine–Melatonin Hybrids

In 2006, Rodríguez-Franco et al. [39] reported hybrids of THA with melatonin, a pineal neurohormone with strong antioxidant action [134] (Figure 4).

Hybrids were potent inhibitors of cholinesterases at the low nanomolar level. Additionally, hybrid **7** is still one of the most potent inhibitors of human AChE described with IC_50_ 0.008 nM. An antioxidant activity of hybrids was determined by the oxygen radical absorbance capacity assay using fluorescein (ORAC-FL); hybrids showed potent peroxyl radical absorbance capacities ranging from 1.7- to 4-fold the value of trolox, a Vitamin E analogue which was used as a standard. Hybrids **1**–**12** proved the ability to cross the blood-brain barrier (BBB) in the PAMPA-BBB test.

In 2009, the same scientific group reported an extended series of THA–melatonin hybrids **14**–**23** [40]. All hybrids were potent inhibitors of mammalian ChEs at the low-nanomolar range. 6-Chloro- and 6,8-dichlorotacrine–melatonin hybrids **3** and **6** showed remarkable selectivity, being from 200- to 1000-fold more active toward hAChE than hBuChE and showing potent peroxyl radical absorbance capacities ranging from 1.5- to 4-fold the trolox value. Molecular modeling studies showed that hybrids target both the CAS and the PAS of AChE. A displacement of the binding of propidium iodide (PI) from the PAS at sub-micromolar concentrations was confirmed. In addition, an inhibition of Aβ self-aggregation and neuroprotective properties in a human neuroblastoma line were reported. 

In 2005, racemic lipocrine, a lipoic acid with a derivative of THA, was reported [135]. This hybrid inhibited AChE effectively (IC_50_ 0.253 nM) and reduced AChE-induced Aβ aggregation from ROS formation. Inspired by this, in 2016 Benchekroun et al. designed THA–melatonin hybrids **24**–**31** with ferulic acid (FA) or lipoic acid [41]. Hybrid **28** was the most effective inhibitor of BuChE with IC_50_ 1.25 nM and AChE with IC_50_ 3.62 nM. Additionally, **24**–**27** were potent antioxidants, showing values from 9.11 trolox equivalents. A neuroprotective effect for hybrids **25**–**27** was shown on SH-SY5Y neuroblastoma cells, with **26** being the best of the series. Additionally, **26** showed a neuroprotective effect against toxic insults mediated by hydrogen peroxide, Aβ_1−40_, and Aβ_1−42_. Hybrids **24**–**27** successfully induced the Nrf2 pathway in the AREc32 reporter cell line.

### 3.2. Other Hybrids with Antioxidant Activity

**Figure 5:**
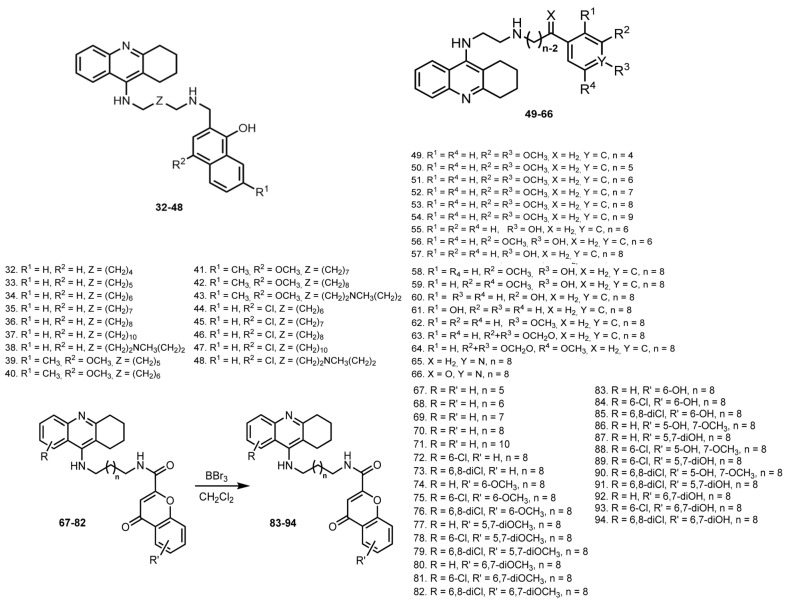
THA–hydroxyquinoline hybrids **32**–**48** [42], THA hybrids with benzene/pyridine moieties **49**–**66** [43,44], and THA–4-oxo-4H-chromene hybrids **67**–**94** [44].

In 2010, Fernández-Bachiller et al. reported THA-based hybrids **32**–**48** with 8–hydroxyquinoline [42] (Figure 5). An inhibition of human cholinesterase showed IC_50_ values for all tested hybrids in the nano- and subnanomolar range (0.5–5.5 nM). Hybrid **40** was the best hBuChE inhibitor with IC_50_ 2 nM. Hybrids conjugated with an unsubstituted 8-hydroxyquinoline fragment and a methylene tether of 7–10 carbons showed the best AChE inhibitory activities, with **35** showing IC_50_ 20 nM. The antioxidant capacity of selected hybrids **35**, **40**, and **45** was confirmed by their competition with fluorescein in the radical capture. Hybrid **35** showed 3.3 trolox equivalents, hybrid **40** showed 2.6, and hybrid **45** showed 4.7.

The affinity of selected compounds for the PAS was confirmed by the displacement of PI. All hybrids showed permeability values over the above limit in the PAMPA-BBB test. The metal-chelating properties of **35** were confirmed by UV-Vis spectrometry in the presence of Cu^2+^. Finally, **35** showed negligible cell toxicity on human neuroblastoma cell line SHSY5Y.

In 2011, Luo et al. reported THA hybrids with substituted benzene or pyridine moieties **49**–**66** [43,44]. Most compounds showed selectivity for BuChE over AChE, and **58** was found to be the best inhibitor for both ChEs with its IC_50_ 4.55 nM and 3.41 nM. Kinetic studies of the inhibition of AChE by **58** revealed a mixed-type inhibition, which was confirmed by a molecular modeling study. The antioxidant activity of **58**–**61** with hydroxyl group was proven via ORAC test; compounds showed potent peroxyl radical absorbance capacities ranging from 1.2- to 2.7-fold of the trolox value. Finally, **58** proved to inhibit self-mediated Aβ aggregation. 

In 2012, Fernández-Bachiller et al. reported hybrids of THA with flavonoid scaffold **67**–**94** derived from 4-oxo-4H-chromene with possible antioxidant and BACE-1 inhibitory activities [45]. Hybrids **67**–**94** showed a selectivity for BuChE, with **70** as the most active inhibitor. Hybrid **88** was the best hAChE inhibitor with IC_50_ 35 pM, and **74** was the most active with IC_50_ 38 pM. Expectedly, hybrids with hydroxyl groups exhibited antioxidant capacities. Hybrid **83** was 1.3-fold more potent than the vitamin E analogue and was the best antioxidant. All hybrids were found to be potent inhibitors of human BACE-1 with IC_50_s from 2 to 22 μM, better than that of apigenin (IC_50_ 38.5 μM), with the most active as **77** (IC_50_ 2.1 μM). All hybrids (except for **92** and **93**) showed the potential to cross the BBB in the PAMPA-BBB test. Finally, **83** showed potent combined inhibition of human BACE-1 and ChEs.

**Figure 6:**
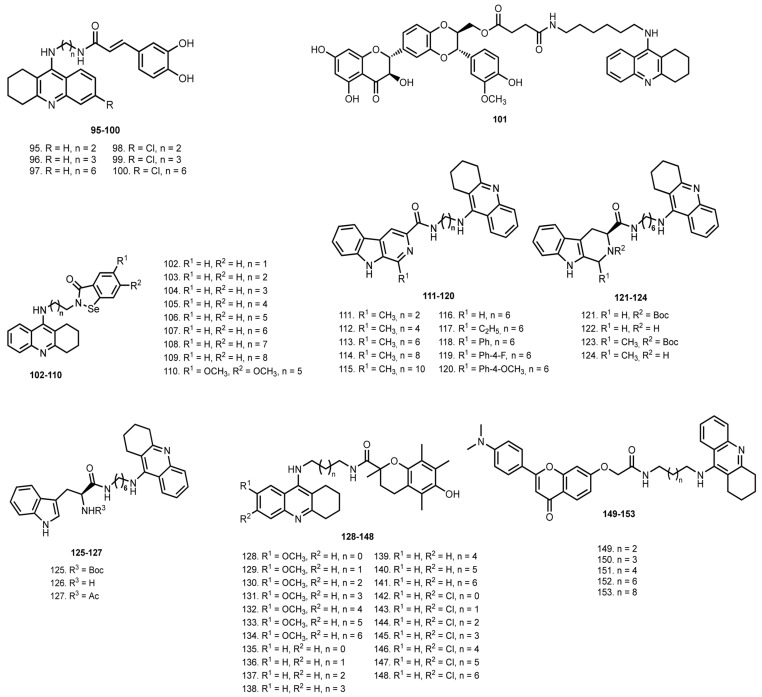
THA–caffeic acid hybrids **95**–**100** [46], THA–silibinin hybrid **101** [47], THA–Ebselen hybrids **102**–**110** [49], THA–(b-carbolines (pyrido [3,4-b]indoles) hybrids **111**–**127** [50], THA–trolox hybrids **128**–**148** [51], THA hybrids with N,N-dimethylated flavonoids **149**–**153** [52].

In 2012, Chao et al. reported hybrids of THA with caffeic acid **95**–**100** [46] (Figure 6). All hybrids inhibited ChEs, with the most potent AChE inhibitor being **99** (IC_50_ 0.3 µM). Expectedly, **95**–**100** showed a radical scavenging activity in a DPPH test due to the presence of a hydroxyl group. Hybrid **99** showed the best antioxidant activity in 4.8 ± 0.9 µM. The inhibition activity of **99** besides Aβ self- or AChE-induced aggregation was proven, as well as its antioxidant properties. Finally, the Cu^2+^–chelating properties of **99** were proven by UV-Vis spectra. 

In 2012, Chen et al. reported a hybrid of THA **101** with a flavonolignan silibinin, a natural antioxidant [47,48,136]. Hybrid **101** loses quite a bit of inhibitory activity at BuChE (16-fold lower) and moderately at AChE (3.5-fold lower) when compared with THA. A lower hepatotoxicity of **101** in comparison with THA was revealed on hepatocellular carcinoma HePG2 cells. No histomorphological changes in liver tissue were observed after administration of **101** in vivo, in contrast to the THA administration. The antioxidant effect of **101** was confirmed by an evaluation of the lipid peroxidation products level in vivo after drug administration. In in vivo behavior tests on scopolamine-injected mice, **101** showed the same pro-cognitive effect as THA. 

In 2013, Mao et al. reported THA hybrids with Ebselen **102**–**110**, an organoselenium with antioxidant activity, anti-inflammatory, and neuroprotective activities [49,137,138]. Hybrids **102**–**110** inhibited both AChE and BuChE with nanomolar activity. Hybrid **106** was the best AChE inhibitor with IC_50_ 6.32 nM among the derivatives with unsubstituted Ebselen moiety, whereas **110** with OMe-substituent showed a promising result in 2.55 nM.

Lineweaver–Burk plots of **110** against AChE revealed a mixed-type inhibition, which was also confirmed by molecular modelling. Expectedly, **106** and **110** proved to be antioxidants with eroxynitrite scavenging activity 1.17 and 1.26 times greater than that of ebselen, respectively. Unfortunately, **110** showed high toxicity on human hepatic stellate cells (HSC).

In 2014, Lan et al. reported THA–(b-carbolines (pyrido [3,4-b]indoles) hybrids **111**–**127** [50]. All hybrids inhibited both ChEs with IC_50_ values from sub-micromolar to nanomolar. Hybrid **122** was the best AChE inhibitor with IC_50_ 21.6 nM and **125** was the best BuChE inhibitor with IC_50_ 4.3 nM. In addition, **122** was the best hAChE inhibitor with IC_50_ 63.2 nM. Kinetic study revealed **122** as a mixed-type inhibitor of AChE, which was also confirmed by molecular modeling studies. Additionally, **122** displayed the most potent antioxidant activity in 1.57 trolox equivalents, and **126** and **122** showed a neuroprotective effect on the rat pheochromocytoma cell line PC12 from H_2_O_2_-induced oxidative stress. Thioflavin T (ThT)-based fluorometric assay with curcumin as a control showed moderate to good antiaggregating potencies of hybrids (22.4–66.5% at 20 µM) with the most effective compounds being **126** and **122**; **122** could also inhibit a Cu^2+^-induced Aβ aggregation. UV–Vis spectrometry revealed the ability of **122** to chelate Cu^2+^ ions. Finally, the PAMPA-BBB assay revealed the ability of hybrids to cross the BBB. 

In 2015, Nepovimova et al. reported hybrids of THA with Trolox **128**–**148**, a water-soluble analogue of vitamin E (±6-hydroxy-2,5,7,8-tetramethylchroman-2-carboxylic acid) and a “gold standard” antioxidant [51]. Hybrids **128**–**148** showed moderate inhibition activity toward hAChE with an IC_50_ from 13.29 to 0.08 μM. Hybrid **148** was the best hAChE inhibitor with IC_50_ 80 nM, four-fold weaker than 6-Cl-THA. Overall, **128**–**148** displayed moderate to good antioxidant capacities, with **148** as the best antioxidant with IC_50_ 44.09 μM. Mixed-type inhibition of AChE was established for **148** by kinetic assay. The low hepatotoxicity of **148** was shown on HepG2 cells, and a metabolic assay in human liver microsomes showed no potentially metabolic products emerging under experiment. Unfortunately, the limitations in the solubility of **148** did not allow the determining of LD_50_ in vivo.

In 2016, Luo et al. reported THA hybrids with N,N-dimethylated flavonoids **149**–**153** [52]. All hybrids inhibited ChEs in the nanomolar range. Hybrid **152** was the best AChE inhibitor with IC_50_ 59.61 nM and **153** was the best BuChE inhibitor with IC_50_ 24.67 nM. The antioxidant activity of hybrids was confirmed using oxygen radical absorbance capacity (ORAC) assay, in which hybrid **153** showed 3.2 trolox equivalents. The antiaggregating activity of hybrids was confirmed by ThT assay. A neuroprotective effect of **152** against H_2_O_2_-induced oxidative stress was shown on PC12 cells, as well as a registered reduction of intracellular ROS levels after treatment with **152**.

**Figure 7:**
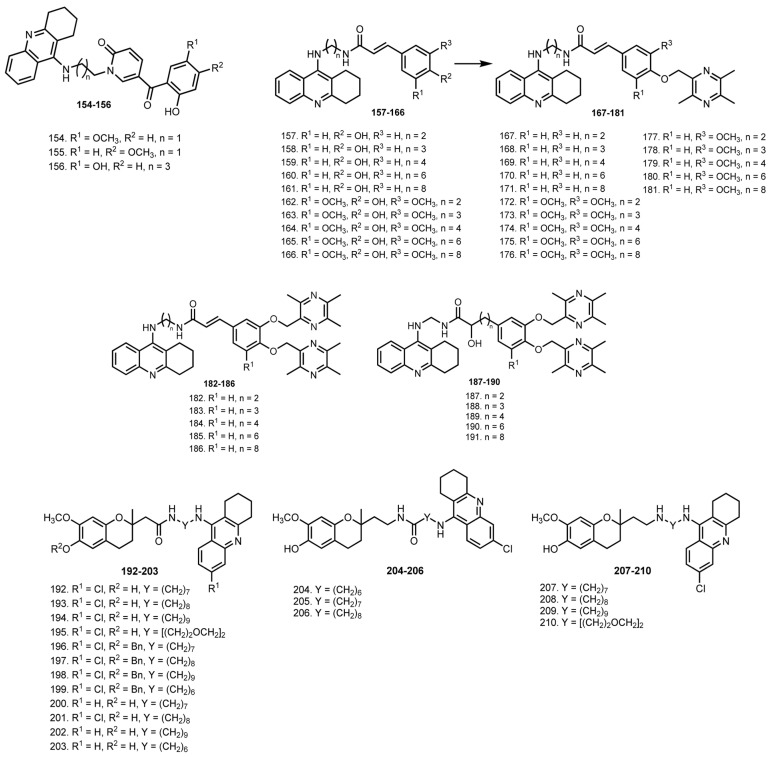
THA–(hydroxybenzoyl-pyridone) hybrids **154**–**156** [53], THA–phenolic acid dihybrids **157**–**166** and THA–phenolic acid–ligustrazine trihybrids **167**–**191** [54], and THA–antioxidant CR-6 hybrids **192**–**210** [56].

In 2016, Chand et al. reported THA–(hydroxybenzoyl-pyridone) hybrids **154**–**156** [53] (Figure 7). All hybrids showed moderate inhibition activity with the most potent being **156** (IC_50_ 0.57 µm against eeAChe). In addition, when antioxidant activity and metal-chelating properties of the hybrid were confirmed, hybrid **156** was the best antioxidant with EC_50_ = 204 μM. 

In 2018, Li et al. designed THA–phenolic acid dihybrids and THA–phenolic acid–ligustrazine trihybrids **157**–**191** [54]. All hybrids showed AChE and BuChE inhibitory activities. Hybrid **165** (IC_50_ 3.9 nM) and trihybrid **175** (IC_50_ 2.6 nM) exhibited the best AChE inhibitory activity. Aso, **165** was a potent inhibitor toward hAChE with IC_50_ 65.2 nM, more effective that THA (IC_50_ 116.8 nM). Kinetic and molecular modeling studies revealed **165** as a mixed-type inhibitor. Additionally, **165** could inhibit the self-mediated Aβ_42_ aggregation, which was confirmed via monitoring of the Aβ aggregation using an atomic force microscope (AFM). Expectedly, **165** showed potent peroxyl radical scavenging capacity with IC50 85.8 μM, and a neuroprotective effect on PC12 cells treated with CoCl_2_ was detected. MTT assay on HepG2 cells revealed a low hepatotoxicity of **165**, and further in vivo tests with ALT and ASAT measurements revealed a lower hepatotoxicity of **165** in comparison with THA.

In 2020, the same scientific group reported significant improvements in cognitive function in APP/PS1 transgenic mice treated with **165** [55]. After 4 weeks of intragastric administration of **165** (1.27 mg/100 g), cognitive function and synaptic plasticity were improved. In addition, the level of Aβ plaques in the DG region in the APP/PS1 mice was reduced. 

In 2020, Pérez-Areales et al. reported THA-based hybrids with antioxidant CR-6 **192**–**210** [56]. The most potent hAChE inhibitors were amines **207** (IC_50_ 442 pM), **208** (IC_50_ 121 pM), and **209** (IC_50_ 272 pM), which were 33-, 120-, and 53-fold more potent than the parent Cl-THA. The order of potencies was as follows: amines **207**–**210** > amides **192**–**195** > reverse amides **204**–**206** > O-benzylated amides **196**–**199**. Expectedly, CR-6–chlorotacrine hybrids were found to be less potent inhibitors toward hBuChE than hAChE. However, amines **207** (IC_50_ 17.4 nM), **208** (IC_50_ 13.4 nM), and **209** (IC_50_ 18.3 nM) were the most potent inhibitors of hBuChE, being 29-, 38-, and 28-fold more potent than the parent 6-Cl-THA. Because 6-Cl-THA is known to interact with CAS [139], the lead compound was expected to interact with AChE in the same way, which was confirmed using molecular dynamics simulations. Kinetic studies showed that the hybrid acts as a mixed-type inhibitor of hAChE. The in vitro antioxidant activity of all hybrids featuring a free hydroxyl group was revealed using DPPH assay, with IC_50_ values in the 6.9–22.9 μM range. Most of the hybrids were found to be inactive as BACE-1 inhibitors, with only the O-benzylated hybrids **196**–**199** showing weak inhibition. Favorable brain permeability of hybrids was confirmed using PAMPA-BBB assay. Chronic in vivo efficacy studies with **193** and **197** in double-transgenic APP/PS1 mice have shown positive tendencies in improving cognition and amyloid pathology.

**Figure 8:**
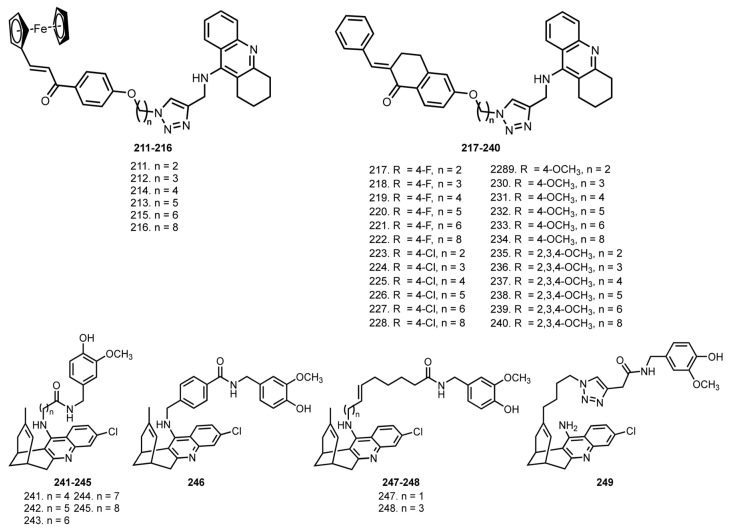
THA–triazole–chalkone conjugates **211**–**240** [57], huprine Y–capsaicin hybrids **241**–**249** [58].

In 2021, Rani et al. reported hybrids of THA with chalcones **211**–**240**, a scaffold with AChE-inhibitory, antioxidant, antiaggregating, anti-inflammatory, neuroprotective, and vasodilator activities [57,140] (Figure 8). Hybrids **216**, **225**, and **226** showed moderate activity against AChE, and only **225** showed above 50% inhibition at 10 µM against the BuChE. Molecular docking studies showed that **225** and **226** interact with residues of AchE. In vivo behavior studies showed that **216**, **225**, and **226** attenuated the effect of scopolamine treatment. Moreover, a recovery of scopolamine-induced glutathione depletion in the mice brain was confirmed in group treated with **216**, **225** and **226**. Additionally, a significant reduction in in-brain malondialdehyde level was detected.

**Figure 9:**
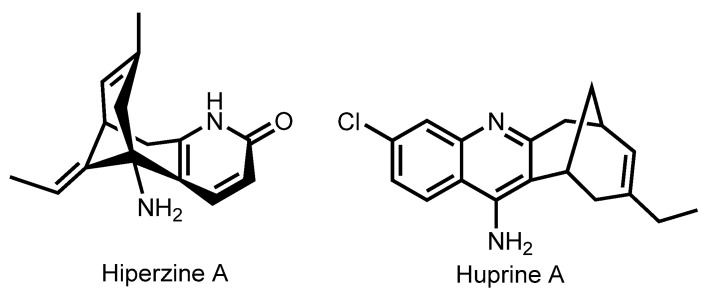
Huperzine A and THA–huperzine analogue Huprine A.

In 2021, Viayna et al. reported huprine Y-based hybrids **241**–**249** with an antioxidant capsaicin [58]. Huprines represent a family of potent and selective AChE inhibitors based on THA and (−)-huperzine A (HA) [141]. HA is a Chinese herb extract from Huperzia serrata, a reversible AChE inhibitor [142]. Despite the approval of HA by the FDA of China for AD therapy in 1994, the supply of this drug is still limited due to expensive synthesis [143,144]. Thus, design of potent HA analogs not requiring great synthetic effort is of interest (Figure 9).

**241**–**249** retained the high potency of the parent huprine Y against hAChE and hBuChE. The nine-atoms linker was found to be optimal for the inhibition of both hAChE and hBuChE, yielding **243**, which surpassed the nanomolar potency of huprine Y. Kinetic studies of AChE inhibition showed a dual site binding, and the interaction of **243** with PAS was confirmed by a PI displacement. All compounds showed antioxidant activity in the DPPH assay, with the best antioxidant being **247** (IC_50_ = 31.7 µM), and proved to be BACE-1 inhibitors, with the most active being **241**. The ability of hybrids to cross the BBB was proven in PAMPA-BBB assay. Additionally, biodistribution studies in C57BL6 mice revealed an ability of **243** and **249** to accumulate in the brain. 

Finally, the therapeutic efficacy of **243** and **249** was investigated in young (5 month) and old (10 month) APP/PS1 mice. Mice treated with **249** enhanced learning and memory in old APP/PS1 mice in all the performed tests, while neither **249** nor **243** were effective in young transgenic mice. A decrease in the Aβ_42_/Aβ_40_ ratio in the brains of mice treated with **249** was revealed. In addition, **249** significantly increased the strength of synaptic transmission, and reduced hippocampal levels of the oxidative stress marker 4-HNE and the neuroinflammation (astrogliosis) marker GFAP.

### 3.3. Tacrine–Ferulic Acid Hybrids

**Figure 10:**
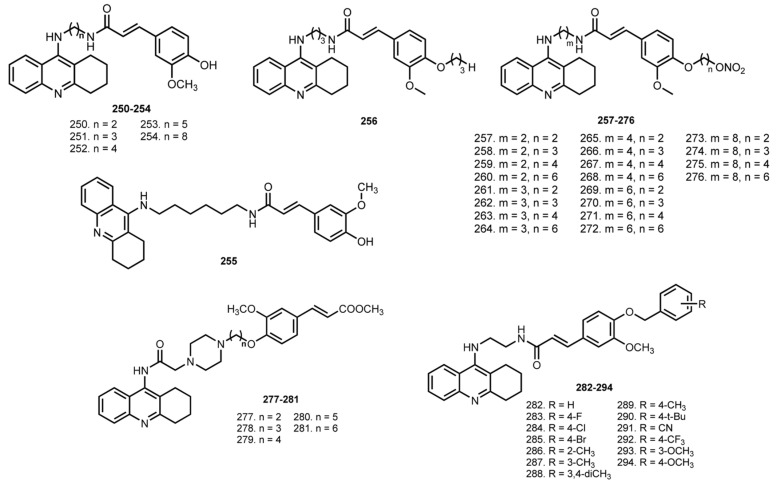
THA–ferulic acid hybrids **250**–**254** [59], **255** [61], THA–ferulic acid–NO-donor thihybrids **257**–**276** and model hybrid **256** [30], THA–ferulic acid hybrids with piperazine linker **277**–**281** [62], and hybrids **282**–**294** [63].

FA is a well-known antioxidant with multiple biological actions [145]. Due to its multiple activity and ease of chemical modifications, the design of FA–THA hybrids is of interest (Figure 10) [146]. 

In 2008, Fang et al. reported THA–FA hybrids **250**–**254** [59]. All compounds inhibited ChEs, with the most potent hybrids being **252** and **253** toward both ChEs, suggesting the optimal length of linker in 6–7 atoms. Kinetic study of AChE inhibition by **253** showed reversible and noncompetitive inhibition. All hybrids showed moderate to good antioxidant activity confirmed by ORAC-fluorescein assay and hybrid **253** showed 1.5 trolox equivalents.

In 2010, an investigation of the in vivo anti-AD effectiveness of **254** was reported [60]. Unfortunately, no beneficial effect on scopolamine-induced cognition impairment was detected. 

In 2012, Pi et al. reported an investigation of anti-AD properties of a similar THA–FA hybrid, **255,** with n = 6 [61]. Hybrid **255** showed an ability to inhibit AChE-induced Aβ aggregation and reduce Aβ-induced oxidative stress in PC12 cells. Thus, 10 µM of **255** reduced the Aβ1–40-induced ROS production in C12 cells. In addition, 255 improved the cognitive impairment, increased ChAT and superoxide dismutase activity, and decreased AChE activity and malondialdehyde (MDA) levels in the Aβ i.c.v. AD model.

In 2012, Chen et al. designed THA–FA–NO donor trihybrids **257**–**276** [30]. NO-donating hybrids showed better or comparable inhibition activity compared to parent **250**–**254** and a decrease in antioxidant activity. The in vitro reactivity of **257**–**276** as NO-donators was confirmed using the Griess reaction [147], in which **262** and **273** showed the height levels of nitrite. Additionally, ex vivo organ bath tests (coronary arteries from rats) vascular relaxation assay for **251**, **257**, **262**, **273**, and **256** revealed a high activity for all hybrids. Hybrid **262** showed a comparable EC_50_ with positive control isosorbide dinitrate (ISDN). Hybrids **251**, **262**, and **256** were active in improving memory impairment in scopolamine-induced mice in a transfer latency time (TLT) test. Importantly, **262** possessed better performance than the non-nitrate hybrid **256**. Finally, the levels of ASAT and ALT in serum of drug-treated mice were determined; **251** and **262** possessed higher safety than THA, and **262** showed the lowest hepatotoxicity.

Fu et al. designed THA–FA hybrids with piperazine linker [62]. All hybrids presented inhibitory activity for both ChEs and selectivity for AChE. The best AChE inhibitors were **279** with IC_50_ 52.7 nM and **280** with IC_50_ 61.7 nM. Low antiaggregating properties of hybrids were revealed, and the Cu^2+^-chelating properties of **280** were confirmed by UV-Vis spectroscopy. Finally, the protective effects of **280** against Aβ-induced neurotoxicity were shown on Neuro-2A cells.

In 2018, Zhu et al. reported THA–FA hybrids **282**–**294** with different substituents in the benzene ring [63]. Hybrid **288** was the most potent AChE inhibitor with IC_50_ 37.02 nM. The presence of electron-withdrawing substituents contributed to the inhibition of BuChE; hybrid **292** with CF_3_ substituent showed IC_50_ 52 nM. Molecular docking showed binding of **288** with both the CAS and PAS of AChE. Further, **285**, **288**, and **291** displayed inhibition on the aggregation of Aβ. When hybrids **285** and **288** were studied for in vivo behavioral analysis in scopolamine-induced cognition-impairment, treatment with **288** led to a remarkable improvement of memory in the scopolamine-induced cognitive impairment in the Morris water maze test. Finally, ALT and ASAT levels were measured after the treatment of animals with **285** and **288**, and the hybrids proved to be safe, which was also confirmed by morphologic results.

## 4. Tacrine Hybrids with NO-Donating Molecules

**Figure 11:**
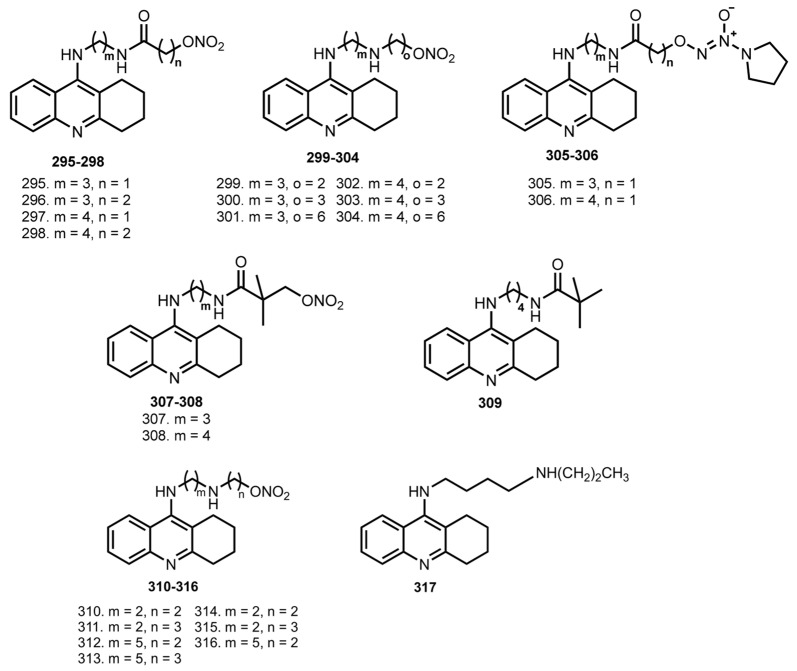
THA hybrids with NO-donating moieties **295**–**308** and model compound **309** [64], hybrids **310**–**316**, and model **317** [65].

Nitric oxide (NO) is a key signaling molecule involved in the regulation of many physiological processes [148]. NO plays roles in regulating synaptic plasticity, neurosecretion, and the sleep-wake cycle, and is considered as a molecule for the treatment of AD, which can cause therapeutic effects by increasing blood supply and regulating cerebral circulation [149,150,151,152,153,154]. In recent years, NO-donating and NO mimetic strategies in AD treatment proved to be effective, and several THA-based hybrids with NO-donating properties were reported (Figure 11).

In 2008, Fang et al. designed and synthesized a series of THA hybrids with NO-donating nitrato- and diazeniumdiolate moieties [64]. With the exception of **308** (226.0 nM), all compounds inhibited AChE with IC_50_ from 5.2 to 93 nM. Inhibition of BuChE was also similar to THA, with IC_50_ values from 5.2 to 41.0 nM. Hybrid **308** showed selectivity for the inhibition of BuChE over AChE, with IC_50_ 7 and 226 nM.

When the vasorelaxation effects of hybrids were accessed via a test with PGF2R-precontracted porcine pulmonary artery, **295**, **303**, and **308** showed moderate effect. When ASAT, lactate dehydogenase (LDH), albumin levels in serum, and concentration of protein in liver tissue after injection of **303** were measured, the hybrid did not show any hepatotoxicity.

In 2008, nitrate–THA hybrids **310**–**317** with shorter and longer diamine side chains were reported [65]. All hybrids retain the ChE inhibitory effect of THA, with the most interesting being **310** (IC_50_ 9.1 nM) and **314** (IC_50_ 7.7 nM). In in vivo tests on the scopolamine-induced cognition impairment animal model, hybrids **295**, **303**, and **310** showed improving recognition activity whereas the analogue **317** did not. This result indicates that the nitrate group of **303** may not only contribute to the vessel relaxant activity, but also is essential for the ChE inhibitory effect. Finally, **295** and **303** did not show obvious signs of hepatotoxicity.

## 5. Tacrine Hybrids with Biological Active Organic Scaffolds

### 5.1. Tacrine–Phenothiazine Hybrids 

**Figure 12:**
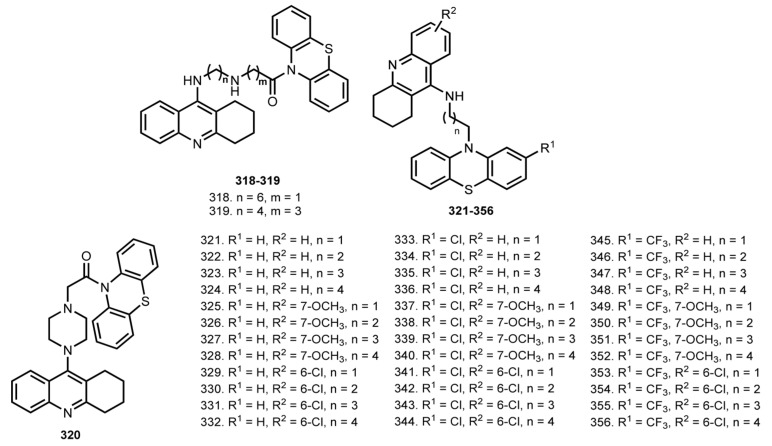
THA–phenothiazine hybrids **318**–**320** [66], **321**–**356** [67].

Phenothiazine is a first-generation heterocyclic anti-psychotic medication that can also prevent tau filament formation [155]. In 2014, Hui et al. reported THA–phenothiazine heterodimers **318**–**320**, which were designed based on molecular docking simulation [66] (Figure 12). Hybrid **318** was the most potent AChE inhibitor with IC_50_ 89 nM. Hybrid **318** proved the ability to reduce P-Tau accumulation in N2a cells, and the ability of **318** to bind Aβ fibrils was confirmed using surface plasmon resonance (SPR).

In 2021, Gorecki et al. reported THA–phenothiazine heterodimers **321**–**356** [67]. All hybrids were potent hChEs inhibitors. 6-Cl-THA-based derivatives were more potent on hAChE (IC_50_ 8–1500 nM) than THA analogues. The most selective compounds were **332** with IC_50_ 8/190 nM and **321** with IC_50_ 2040/15 nM. All compounds showed a toxicity on HePG2 cells in the micromolar range. Hybrids **330**, **332**, **336**, and **344** proved their potential ability to cross the BBB. 6-Cl-THA-based hybrids showed the ability to inhibit τ (**306**–**336**) aggregation; the chain length was found to influence the inhibitory potency, with optimal 2–3 methylene units. Length of the linker also proved to be crucial in self-induced Aβ aggregation, with hybrids **332** and **335** showing the best inhibitory potency. Finally, in vivo safety studies revealed a good tolerance of hybrid **332**.

### 5.2. Tacrine–Benzotiazole/Benzofuran Derivatives

**Figure 13:**
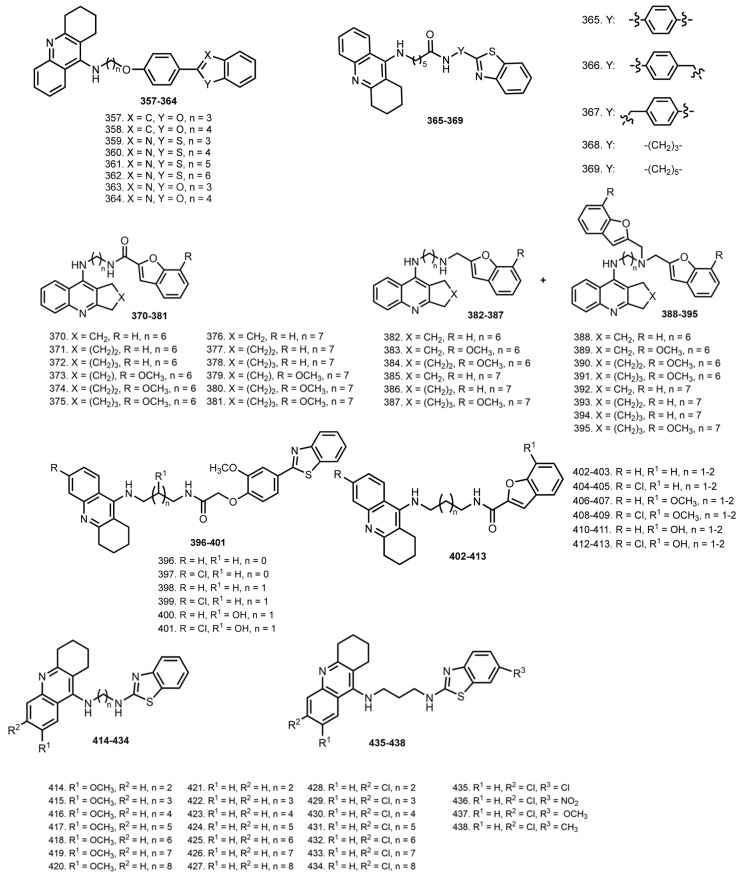
THA–benzofuran **357** and **358** and THA–benzotiazole hybrids **359**–**364** [68], THA–benzotiazole hybrids **365**–**369** [69], THA–benzofuran hybrids **370**–**395** [70], THA–benzotiazole hybrids **396**–**401** [71], THA–benzofurane hybrids **402**–**413** [72], THA–benzotiazole hybrids **414**–**434** and **435**–**438** [73].

Hybrids with Aβ-affinic benzofuran/benzotiazole moieties were repeatedly designed as dual action drugs capable of both Aβ binding and cholinesterase inhibition (Figure 13). Both benzotiazole and benzofuran are well-known scaffolds for Aβ binding; thus, ^11^C-Pittsburgh Compound-B (PiB) is a non-invasive tool for amyloid imaging in humans [156,157]

Pioneer THA–benzofuran/benzotiazole conjugates were reported by Huang et al. [68]. Hybrids **357**–**364** inhibited ChEs with IC_50_ values in the micromolar range. Hybrid **359** exhibited the most potent inhibition of AChE with IC_50_ 0.017 µM. Kinetics study of AChE inhibition showed **359** to bind both the CAS and PAS of AChE. The ability of hybrids to inhibit Aβ aggregation was assessed by the ThT assay, in which **359** demonstrated similar Aβ aggregation inhibitory activity with curcumin.

In 2013, Keri et al. also designed THA–benzotiazole hybrids **365**–**369** [69]. All hybrids displayed high inhibitory activities the against AChE enzyme, and with IC_50_ values in the micromolar range **365** was chosen as the lead compound with IC_50_ 0.34 µM. All the compounds showed some ability to inhibit the Aβ_42_ self-aggregation, which was confirmed by ThT assay. 

In 2016, Zha et al. reported hybrids **370**–**395** based on THA, as well as its analogues with different side cycle size, with benzofuran scaffolds [70]. The inhibitory activities against hAChE ranged from 7.49 µM to 0.86 nM. The most potent hybrid, **386**, showed a subnanomolar inhibitory potency, 493 times more potent than THA. The most selective hBuChE inhibitors were hybrids with hexamethyl chain **388**–**391**. Hybrid **386** was also the only derivative slightly selective for hAChE (2.5-fold). hBuChE inhibitory activity was associated with the presence of a 7-methoxy substituent on the benzofuran nucleus. The highest hBuChE inhibition was achieved with hybrids **384** and **390** (0.49 nM and 0.48 nM). Kinetic study of hAChE inhibition by **386** revealed mixed-type inhibition. Further, the ability of **386** to inhibit AChE-induced Aβ fibril formation was confirmed by a ThT assay. An inhibition of Aβ self-aggregation and an inhibitory potency against hBACE-1 were confirmed. Finally, treatment of scopolamine-induced ICR mice with **386** led to considerable amelioration in cognition impairment. In ASAT and ALT levels measurements in serum after treatment with similar doses of **386**, THA and bis-THA were measured and revealed low hepatotoxicity of **386**.

In 2019, THA–benzothiazole hybrids **396**–**401** were reported by Rajeshwari et al. [71]. The docking study revealed favorable interactions of hybrids with THA moiety binding CAS. All hybrids inhibited AChE (IC_50_ 0.06–0.27 µM), but **397** exhibited the best inhibitory activity (IC_50_ 0.06 µM). In addition, all hybrids inhibited Aβ self-aggregation. A neuroprotective effect of hybrids was confirmed on SH-SY5Y cells treated with Aβ peptide or ascorbate/iron. Hybrids **397**, **398,** and **401** prevented Aβ-induced cell toxicity; **396**, **398**, **399,** and **401** also showed the ability to inhibit Aβ-self-aggregation. However, the log BB assessment showed that hybrids are not drug candidates for oral administration.

In 2020, Fancellu et al. reported THA–benzofurane hybrids **402**–**413** [72]. The best AChE inhibitors were **404**, **408**, and **412** with IC_50_ 0.12, 0.13, and 0.13 µm. Additionally, all hybrids exhibited inhibitory activity in self-induced Aβ aggregation, and hybrids with the OH-group also showed high activity in Cu^2+^- induced Aβ aggregation. For **410**, an anti-aggregating activity was also confirmed using transmission electron microscopic (TEM) images. Hybrids **408** and **412** prevented Aβ-induced cell toxicity, and **408** also showed cell protection from Asc/Fe-induced oxidative stress.

In 2021, Nepovimova et al. reported THA–benzotiazole hybrids **414**–**438** [73]. All hybrids were potent hAChE inhibitors with IC_50_ values in the micromolar to nanomolar range. Hybrids based on 7-MEOTA (**414**–**420**) displayed the poorest inhibition of hAChE; THA-based hybrids (**421**–**427**) showed moderate results, whereas hybrids based on 6-Cl-THA (**428**–**434**) were the best hAChE inhibitors. Based on a set of test results, hybrid **436** was chosen as lead, and its interactions with AChE were simulated by molecular docking. 6-Cl-THA moiety was found to occupy the PAS of hAChES, in contrast to its previously reported CAS binding [139]. The antiaggregating potential of **414**–**434** was confirmed using ThT assay. Additionally, the inhibition effects of **416** and **429** were confirmed using steady-state fluorescence and microscopy techniques. In addition, **436** showed the lowest hepatotoxicity, which was confirmed by the MTT test. The BBB penetration ability of hybrids was confirmed by a PAMPA-BBB test. Finally, an insignificant therapeutic effect of **436** was observed in scopolamine-treated mice.

### 5.3. Tacrine Hybrids with NSAIDS

Inflammation is an important therapeutic target, and is one of the important factors in clinical symptoms of AD [158]. Thus, nonsteroidal anti-inflammatory drugs (NSAIDs) are of interest in AD therapy [159]. NSAIDs were reported to reduce inflammatory markers and reverse spatial memory deficits in APPsw transgenic mice or improve memory and learning, and decrease stress-related behaviors in FAD5X/Ppara-null mice [160,161,162]. 

**Figure 14:**
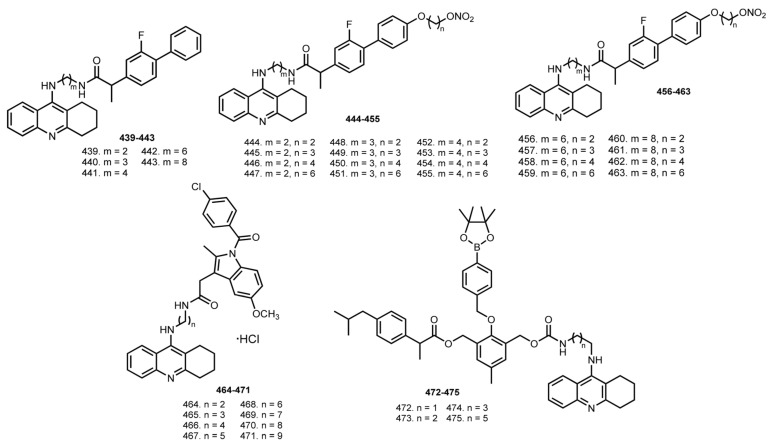
THA–flurbiprofen hybrids **439**–**443** [74], THA–flurbiprofen–NO–donating hybrids **444**–**455** [75], **456**–**463** [76], THA–indometacine hybrids **464**–**471** [77], ROS–responsive ibuprofen–THA hybrids **472**–**475** [78].

In 2013, Chen et al. reported THA–flurbiprofen hybrids **439**–**443** [74] (Figure 14). Hybrids **442** and **443** showed high activity toward both ChEs. Additionally, hybrid **442** showed a reduction in Aβ_40_ formation.

Similar hybrids **444**–**455** fortified with NO-donating ability were designed [75]. All hybrids showed comparable or better BuChE inhibitory activity (IC_50_s 3.9–13.9 nM) than parent hybrids **439**–**441**. The best results were obtained for **447** and **455**, with IC_50_ 4309.5 and 1456.4 nM against AChE. Kinetic study revealed a mixed-type inhibition of **447**. All hybrids showed promising levels of nitrite generated in Griess reactions. Vasorelaxation activity of **447** and **455** was confirmed in an ex vivo organ bath (coronary arteries from rats).

The same scientific group reported hybrids **456**–**463** [76]. All hybrids (ex. **463**) exhibited similar or higher inhibitory activities compared with THA. The most potent hybrids were **456** and **460** with IC_50_ 9.1 and 12.5 nM on AChE and IC_50_ 2.5 and 1.0 nM against BuChE. Kinetic study of ChE inhibition for **456** revealed mixed-type inhibition. Griess reaction revealed the NO-releasing ability of all hybrids **456**ȓ**463**. A vascular relaxation effect of **456** and **460** was confirmed on the coronary arteries of rats. 

Behavior studies in vivo were performed using a scopolamine-induced impairment in passive avoidance test. An improving memory impairment in the group treated parent with **442** (hybrid without NO-donating group), and the group treated with **456** was observed compared to the scopolamine group. Hybrid **456** showed no difference in comparison with THA and hybrid **442** (*p* > 0.05). ASAT and ALT levels were determined after mice were treated with THA, hybrids **442**, and **456** at equimolar doses. Hybrids **442** and **456** displayed higher safety than THA, and NO-donating hybrid **456** showed the lowest hepatotoxicity. 

In 2021, Zawada et al. reported THA–indometacine hybrids **464**–**471** [77]. The IC_50_ values for hybrids range from 10 to 260 nM. The most active compound against AChE was **471** (IC_50_ 10 nM). Kinetic study revealed a mixed-type inhibition for **471**. A low toxicity on the HepG2 and EA.hy926 cells was revealed. Moreover, **471** showed antioxidant effects in DDPH and ABTS studies.

In 2021, Liu et al. designed ROS-responsive ibuprofen–THA hybrids **472**–**475** [78]. Low neurotoxicity of hybrids was proven on SH-SY5Y cells. Neuroprotective activities of **475** against H_2_O_2_, and H_2_O_2_-scavenging capability were shown. An ability of **475** to degrade into counterparts in the presence of H_2_O_2_ was confirmed by HPLC. 

Hybrids **472**–**475** showed moderate or no obvious AChE inhibitory activity in the absence of H_2_O_2_. An ability of **475** to inhibit proinflammatory cytokines TNF-α and IL-1β in endotoxin lipopolysaccharide (LPS)-treated microglial cells (BV-2) was revealed, as well as the regulating of apoptosis-related proteins. In addition, **475** showed neglected hepatotoxicity in HepG2 cells. Finally, the therapeutic effect of **475** and the improving spatial memory of Aβ-induced AD model rats were confirmed. 

### 5.4. Tacrine–Hupyridone Hybrids

**Figure 15:**
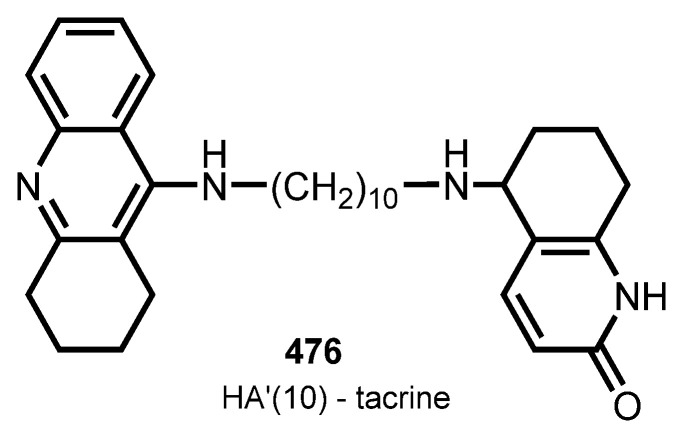
Hybrid HA’(10)–THA **476** [164].

Pyridones have been utilized as privileged scaffolds in drug discovery [163]. THA–hupyridone hybrids were first described in 1999 [164,165]. In 2007, Li et al. summarized a study of therapeutic efficacy of several THA dimers, including THA–hupyridone hybrids [166]. In addition, in 2021 Mak et al. summarized multifunctional dimers, including THA–hupyridone [167]. Herein, we will provide the most potent hybrid HA’(10)–THA **476**, first described by Carlier et al. [164] (Figure 15).

**476** possessed a nanomolar AChE inhibition (IC_50_ 8.8 nM). Since both THA and huperzine A can increase the expression of brain-derived neurotrophic factor (BDNF) in the brain, **476** was also suggested to elevate BDNF expression concurrently [168,169].

In 2018, Chen et al. reported **476** to prevent the surgery-induced decrease in BDNF in the hippocampus of aged mice [79]. Hybrid **476** might act on BDNF to enhance cognitive performance. Additionally, **476** proved to increase the expression of pAkt and pERK, and ChAT-positive area in the hippocampal regions of surgery-treated mice proved to effectively attenuate scopolamine-induced cognitive impairments in vivo and be less toxic than THA [80].

Recently, Xuan et al. reported that **476** produces cognitive-enhancing effects in APP/PS1 and Aβ oligomers-treated mice [81]. Neuroprotective effects of **476** were proved, including the inhibition of Aβ aggregation, the activation of the BDNF/TrkB pathway, the alleviation of neuroinflammation, and the decrease in AChE activity.

### 5.5. Tacrine–Donepezil Hybrids

Donepezil is a specific and reversible inhibitor of AChE, and is an FDA-approved drug for the symptomatic treatment of AD [170]. THA–donepezil hybrids were designed by different scientific groups. The first donepezil-THA hybrid was reported in 2004 by Shao et al. [82].

**Figure 16:**
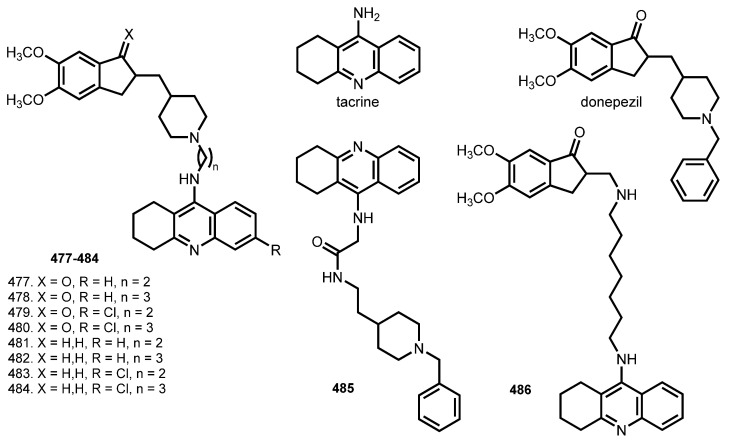
Donepezil, THA, donepezil–THA hybrids **477**–**484** [83], and previously reported donepezil–THA hybrids **485**, **486** [82].

Camps et al. reported [83] donepezil-THA hybrids in order to obtain more effective AChE inhibitors than previously reported by Shao et al. and Alonso et al. in 2004 [82,171] (Figure 16).

All hybrids were hAChE inhibitors, exhibiting IC_50_ values in the subnanomolar range. Hybrids **477**–**484** were more potent AChE inhibitors than were **485** (IC_50_ 6.0 nM) and **486** (IC_50_ 25 nM). Hybrid **480** was the most effective inhibitor (IC_50_ 90 pM). Hybrids **477**, **478, 481**, and **482** were more potent BuChE inhibitors than was **485** (IC_50_ 76 nM), though none of them was as potent as **486** (IC_50_ 0.6 nM). Hybrids **479** and **480** proved their ability to bind with PAS via displacement of ThT. In addition, six out of the eight hybrids exhibited an Aβ antiaggregating activity.

### 5.6. Tacrine–TPPU Hybrids

1-trifluoromethoxyphenyl-3-(1-propionylpiperidin-4-yl) urea (TPPU) is a potent soluble epoxide hydrolase (sEH) inhibitor [172]. sEH are able to metabolize epoxyeicosatrienoic acids (EETs), which reduce inflammation and oxidative stress, by epoxide ring opening to the corresponding diols by the soluble epoxide hydrolase [84]. sEH inhibition is a promising strategy for the treatment of pain, inflammation, cardiovascular diseases, and other conditions [173].

**Figure 17:**
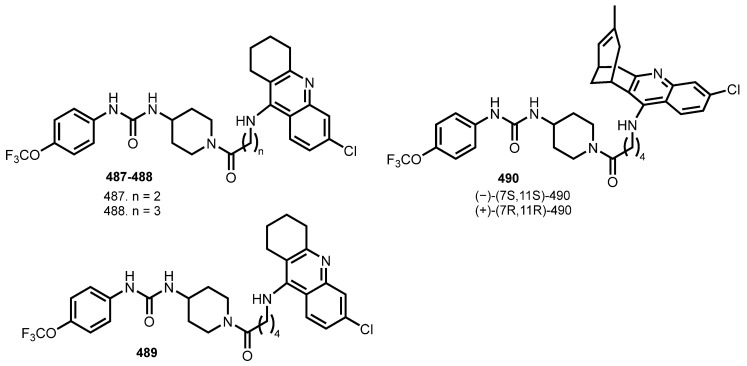
6-Cl-THA–TPPU **487**–**489** and huprine–TPPU hybrids (−)-(7S,11S)-490, (+)-(7R,11R)-490 [84].

In 2022, Codony et al. reported hybrids **487**–**489** with dual targeting of sEH and AChE [84] (Figure 17). Dual inhibitors were designed by linking the scaffolds of TPPU, 6-Cl-THA, and huprine.

Most of the hybrids displayed well-balanced potencies in the low nanomolar range when tested in vitro on the two recombinant human enzymes, hsEH and hAChE. All hybrids retained the hsEH inhibitory activity of TPPU (IC_50_ 3.7 nM) with IC_50_s in the subnanomolar to low nanomolar range, with hybrids **487** and **(−)-490** displaying an even higher potency. Regarding hAChE inhibition, **487** and **489** retained the potency of 6-Cl-THA, and **488** was five-fold more potent. **(−)-(7S,11S)-490** proved to be 850-fold more potent than its enantiomer, in line with the eudismic ratio of huprine Y. Hybrid **489** was chosen as the lead, with IC_50_ 12.9 nM against hAChE and IC_50_ 179 nM against hBuChE. Molecular dynamics simulations revealed **489** to interact with both sites of AChE. Hybrid **489** was the most stable compound in human microsomes. In vivo investigation in senescence-accelerated mouse-prone 8 (SAMP8) revealed a significant amelioration in short-term and long-term working memory after oral administration of **489** (2 mg kg/day).

### 5.7. Tacrine–Huprine Hybrids 

**Figure 18:**
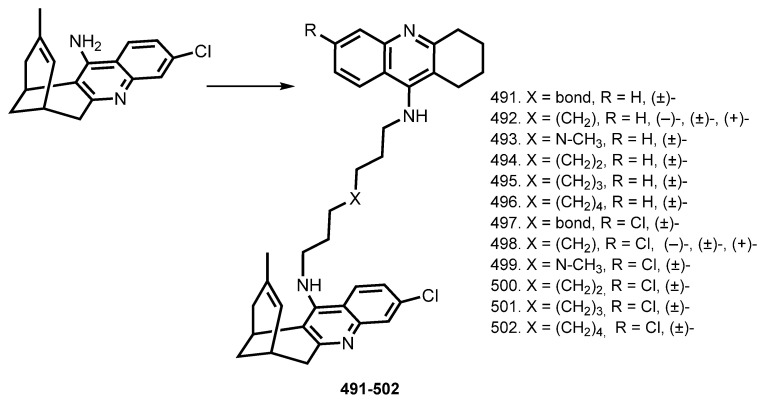
Huprine−tacrine heterodimers **491**–**502** [85].

Huprine is a potent AChE inhibitor based on THA scaffold [174]. In 2012, Galdeano et al. reported enantiopure huprine—THA heterodimers **491**–**502** [85] (Figure 18). Hybrids in racemic form **(±)-491, (±)-493**–**(±)-497**, and **(±)-499**–**(±)-502**, as well as the enantiopure (−)-(7S,11S)- and (+)-(7R,11R)-heptamethylene-linked heterodimers **(−)-492**, **(+)-492**, **(−)-498**, and **(+)-498** were synthesized and their biological activities were investigated. 

Expectedly, ChEs inhibitory activity was governed by spacer length. The levorotatory (7S,11S)-huprine-based heterodimers were the eutomers with regard to hAChE inhibition, with **(−)-492** and **(−)-498** being five- to six-fold more potent than the dextrorotatory enantiomers. The most potent hybrids were racemic **(±)-493** and **(±)-499**, and **(±)-491** and **(±)-497**. Additionally, heterodimers inhibited hAChE-induced Aβ aggregation and blocked the chaperoning effect of AChE on PrP106−126 aggregation. An activity of heterodimers **(−)-492**, **(+)-492, (±)-492**, **(±)-494**, and **(±)-495** toward self-induced Aβ aggregation and BACE-1 inhibition of hybrids **(±)-496, (−)-498**, **(+)-498**, and **(±)-500**–**(±)-502** were revealed. Finally, ex vivo experiments proved the ability of **(±)-494** and **(±)-500** to cross the BBB and inhibit in-brain AChE activity. 

### 5.8. Tacrine–Bifendate Hybrids 

**Figure 19:**
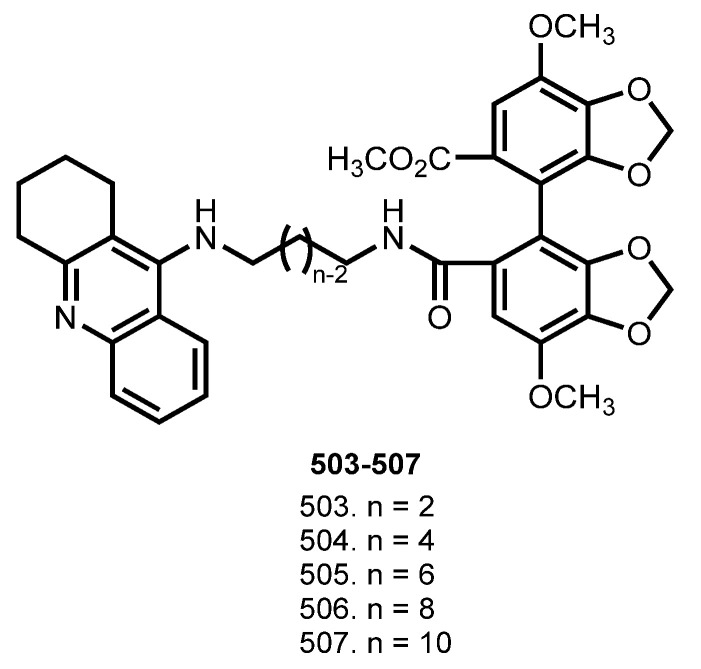
THA -Bifendate hybrids **503**–**507** [86].

In 2018, Cen et al. reported hybrids of THA with Bifendate (**503**–**507**), which is used for the treatment of chronic viral hepatitis B in China, and which was also reported to protect the liver mitochondria in mice from THA-induced injury [86,175,176] (Figure 19).

Hybrids **503**–**507** showed potent inhibitory activities at the nanomolar concentrations and good selectivity for BuChE. Hybrid **506** was the most potent AChE inhibitor (IC_50_ 27.32 nM). Hybrid **504** was the most potent inhibitor of BuChE (IC_50_ 4.02 nM). In addition, **506** showed a high inhibition of hAChE. Hybrids **503**–**507** prevented the self-mediated Aβ aggregation, and the antiaggregating potential of **506** was confirmed by TEM study. Low hepatotoxicity of **506** was confirmed on HepG2 and HL-7702 cells. No increases in ALT and ASAT levels were observed after the administration of **506** in mice, but amelioration of the cognition functions in the scopolamine treated ICR mice was proven. 

### 5.9. Tacrine hybrids with HDAC Inhibitors

Histone deacetylases (HDACs) are generally considered as therapeutic targets in the treatment of AD. The roles of histone deacetylases HDACs on cognitive impairments have been demonstrated in studies of AD animal models [177]. Furthermore, different types of HDACs may have distinct roles in the cognitive changes of AD. 

**Figure 20:**
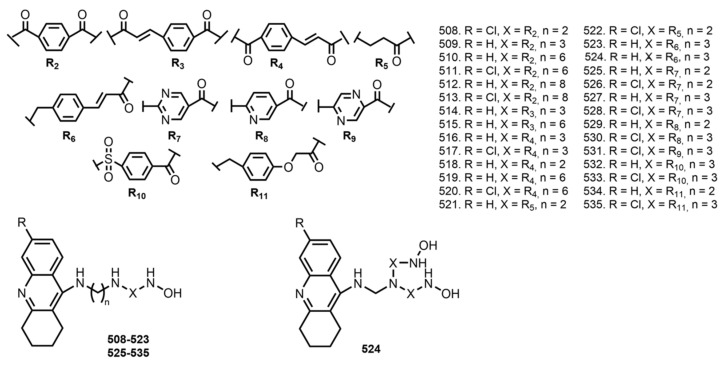
THA (6-Cl-THA)–HDAC inhibitors hybrids **508**–**535** [87].

In 2020, Xu et al. reported THA-based hybrids **508**–**535** with HDAC inhibitors [87] (Figure 20). Well-established pharmacophore models such as SAHA, LBH589, and PXD101 [178] were used. 

All hybrids inhibited ChEs, with improved inhibition on AChE compared to THA. Hybrids **517** and **535** were the most potent inhibitors of AChE (IC_50_ 0.12 and 0.26 nM). The inhibitory potency on HDACs of hybrids **508**–**535** was determined against HeLa nuclear extract. Hybrids **511**, **517**–**520**, **523**–**524**, **528**, and **535** showed superior or comparable inhibitory potency when compared with reference compounds SAHA or PXD101; hybrids **517**, **520**, and **524** were the most potent, with IC_50_ 0.23, 0.32, and 0.28 nM. An antioxidant activity of all hybrids (except **520**) was confirmed using ABTS assay. Hybrids **508**–**535** also exhibited an inhibition of Aβ self-aggregation. An ability of hybrids to chelate copper ions was confirmed. Kinetic study of AChE inhibition by **517** revealed mixed–type inhibition. Additionally, hybrids **512**, **517**, **519**, and **524** were predicted as a BBB penetrant. 

### 5.10. Tacrine Hybrids with Thio Derivatives

**Figure 21:**
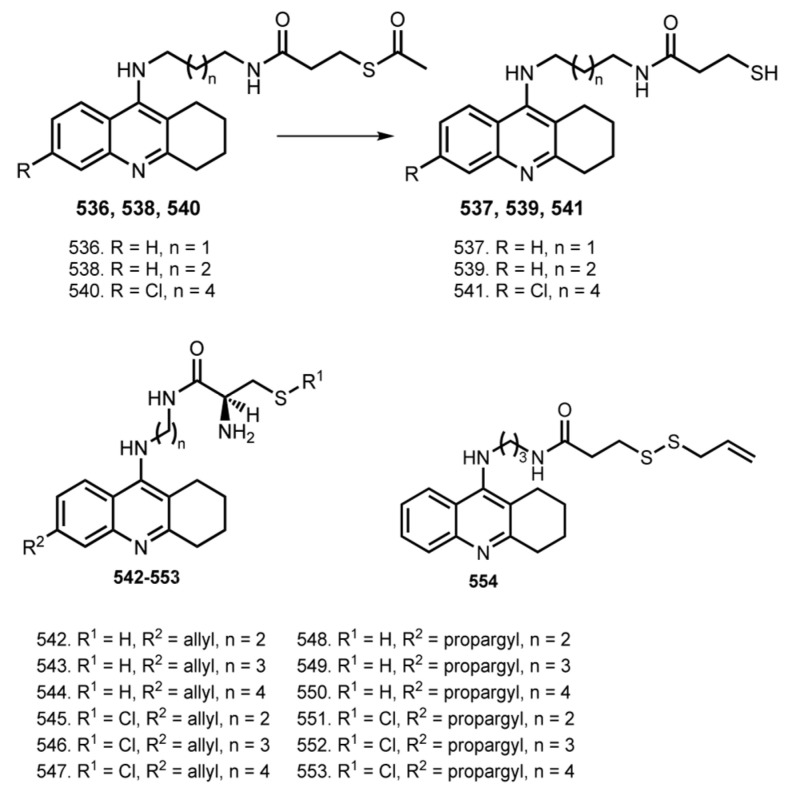
THA derivates conjugated with mercapto group **536**–**541**, designed by Wang et al. [88], THA and 6-Cl-THA–based hybrids **542**–**553** with allyl and propargyl derivatives of cysteine [89], hybrid with H_2_S-releasing moiety **554** [90].

Modulation of synaptic plasticity, especially the long-term potentiation (LTP), has been proposed as a potential therapeutic strategy for improving cognitive function of AD patients [179]. Compounds with mercapto group, such as dithiothreitol (DTT), glutathione (GSH), and N-acetyl cysteine (NAC), can facilitate the induction of LTP in normal rats and even reverse the LTP impairment in aged rats [180]. Inspired by this, several THA hybrids with thio derivatives were reported (Figure 21).

In 2012, Wang et al. designed THA derivates **536**–**541** conjugated with mercapto group [88]. Hybrids generally retained the ChE inhibitory effect, and **540** displayed the most potent inhibitory activity against AchE with pIC_50_ 7.37 ± 0.02. Mercaptotacrine derivatives were more potent inhibitors of BuchE than AchE, similar to that of THA, except for **540**. Effects of hybrids on high-frequency stimulation (HF)-induced LTP in the CA1 region of Sprague−Dawley rat hippocampal slices were evaluated and an increase in the magnitude of LTP during the incubation of hippocampal slices with **537** and **541** was detected. Additionally, an enhanced hippocampal LTP after intracerebroventricular (icv) injection of **541** was detected in vivo. A neuroprotective action of **536**, **538**, and **540** against H_2_O_2_ -induced oxidative stress was proven on human neuroblastoma cell line SH-SY5Y. All hybrids showed neuroprotective effects in a concentration-dependent manner, whereas hybrids **537**, **539**, and **541** presented a U-shaped dose-protection dependency. Finally, AST and ALT activities in serum samples were measured after administration of the drugs in vivo; hybrids **536** and **537** showed little hepatotoxicity. 

In 2016, Keri et al. reported THA and 6-Cl-THA-based hybrids **542**–**553** with allyl and propargyl derivatives of cysteine [89]. Among the compounds investigated, the 6-Cl-THA-based hybrids presented high inhibitory activity in the submicromolar range, with the most active being **545** (IC_50_ 0.30 µM against AChE). Hybrids **542**, **547**, **552**, and **553** showed neuroprotection from H_2_O_2_-mediated oxidation on SH-SY5Y cells. 

Levels of H_2_S and activity of its synthesized enzyme cystathione β-synthase (CBS) are severely reduced in the brains of AD patients [181,182]. Treatment with H_2_S or a H_2_S donor improves cognitive function in AD patients and rat models [183]. 

In 2019, Cheng et al. reported THA-based hybrid **554** with H_2_S-releasing moieties (ACS81) [90]. Hybrid **554** improved cognitive and locomotor activity in AD mice, while also reducing inflammation and increasing synaptic plasticity in the hippocampus. Furthermore, hepatotoxicity studies confirmed that **554** was much safer than THA. Treatment with **554** was able to inhibit the AChE levels in the serum and hippocampus of AlCl_3_-treated AD mice with comparable effects to THA. Additionally, **554** inhibited hippocampal inflammation, as evidenced by the decreased mRNA expression of proinflammatory cytokines (TNF-α, IL-6, and IL-1β). Hybrid **554** also increased hippocampal H_2_S levels, decreased inflammation, and improved synaptic plasticity in the hippocampus. Importantly, **554** did not show evidence of hepatotoxicity or liver inflammation as measured by hepatic transaminases and proinflammatory cytokines.

### 5.11. Tacrine Hybrids with Fluorescent Probes

**Figure 22:**
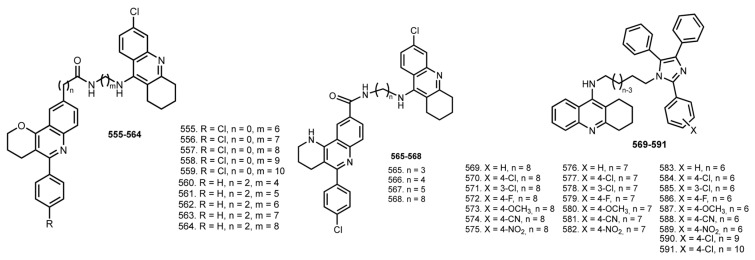
6-Cl-THA-5-phenylpyrano [3,2-c]quinoline hybrids **555**–**564** [91], 6-Cl-THA–tetrahydrobenzo[h][1,6]naphthyridine **565**–**568** [92], THA–lophine hybrids **569**–**591** [93].

PI is well-known to bind the AChE [184]. Inspired by PI structure, in 2009 Camps et al. designed 6-Cl-THA-based hybrids **555**–**564** with 5-phenylpyrano [3,2-c]quinoline [91] (Figure 22). All hybrids showed nanomolar activity toward ChEs. The most potent hAChE inhibitor was **557**. Hybrids **560**–**564** turned out to be two- to three-fold more potent toward the human enzyme, with no significant dependency on the length of the linker. Hybrids **560**–**564** expectedly proved to be more potent BuChE inhibitors that chloro-substituted **555**–**559**, with IC_50_ values in the nanomolar range, up to three- to four-fold more potent than 6-Cl-THA. Molecular modeling and kinetic studies confirmed the dual site binding to hAChE. Hybrids **560**, **563**, and **564** can be considered as moderate inhibitors of Aβ self-aggregation. In addition, **564** showed as a potent BACE-1 inhibitor. Finally, these hybrids are able to cross BBB according to PAMPA-BBB assay. 

In 2014, Pietro et al. reported similar THA hybrids with tetrahydrobenzo[h][1,6]naphthyridine [92].

All the 6-Cl-THA-based hybrids turned out to be potent inhibitors of hAChE, with **565** being the most potent (IC_50_ 6.27 pM). Additionally, **565**–**568** exhibited an inhibition of Aβ_42_ and tau aggregation. All hybrids were predicted to cross the BBB.

In 2013, Costa et al. reported THA-based hybrids **569**–**591** with 2,4,5-triphenyl-1H-imidazole (lophine), which can be used as a fluorescent-labeling reagent and was reported as a ChE inhibitor [93,185]. Hybrids **569**–**591** were found to be potent inhibitors, with IC_50_ in the nanomolar range. The most active AChE inhibitor was **570** (IC_50_ 5.87 nM) and the most active BuChE inhibitor was **581** (IC_50_ 7.10 nM), which was inactive toward AChE.

### 5.12. Tacrine Hybrids with Ca^2+^ Channel Blocker

**Figure 23:**
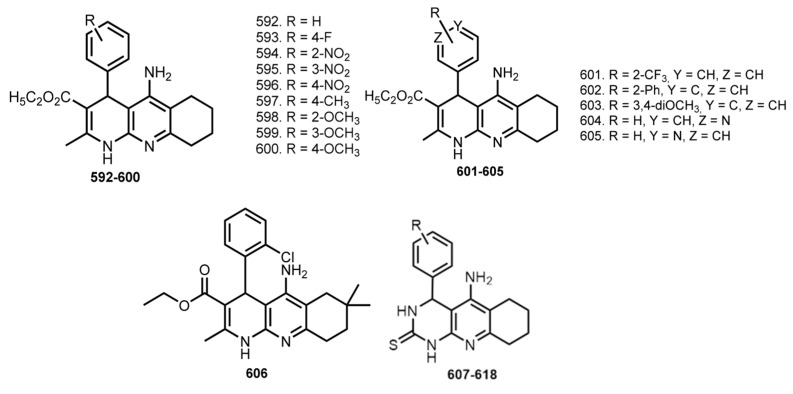
THA–nimodipine hybrids **592**–**600**, **601**–**605** [94,95], THA–dihydropyridine hybrid **606** [97], THA-dihydropyirimidine-thione hybrids **607**–**618** [98].

In 2006, Marco-Contelles designed hybrids **592**–**600** in which the aromatic moieties of THA are surrogated to nimodipine-like moiety [94] (Figure 23). Nimodipine is an FDA-approved selective blocker of L-type voltage-dependent Ca^2+^ channels [186]. 

The most potent inhibitor of AChE was **600** (IC_50_ 45 nM). Hybrids **593** and **599** were also of high potency and excellent selectivity for AChE. A Ca^2+^ influx induced by K^+^ depolarization in SH-SY5Y cells was evaluated. Most of the hybrids demonstrated a significant Ca^2+^ blockade, with the most potent being **598** with a blockade similar to that obtained for nimodipine. Finally, **593**, **599**, and **600** showed neuroprotective properties against Ca^2+^ overload and H_2_O_2_-induced oxidative stress on SH-SY5Y cells.

In 2009, an expanded series of THA–nimodipine hybrids (**601**–**605**) was reported [95]. Most of the tacripyrines were more potent inhibitors of AChE than THA. Hybrid **600** was again chosen as the lead compound (IC_50_ 45 nM). Molecular modeling results showed binding of **(R)-600** with PAS. Hybrid **600** proved to be an inhibitor of AChE-induced Aβ_40_ aggregation and Aβ_42_ self-aggregation. Most hybrids promoted significant Ca^2+^ blockade, with the most potent being **604**, whose activity was similar with nimodipine. PAMPA-BBB assay showed that almost all tacripyrines could cross the BBB and reach their biological targets.

In 2011, the same scientific group provided the pharmacological analysis of both enantiomers of **600** [96]. Both enantiomers showed similar results in inhibiting cholinesterase activity, AChE-induced Aβ aggregation, and Aβ self-aggregation in vitro. **(S)-600** afforded significant protection against Aβ_25–35_-induced toxicity when tested on SH-SY5Y cells.

In 2015, Xiu-Lian et al. reported a similar THA-based hybrid **606** [97]. Hybrid **606** in low concentrations proved its ability to reduce tau phosphorylation levels, which was confirmed on HEK293/tau cells. In addition, its ability to inhibit the generation and release of Aβ was confirmed on mouse neuroblastoma N2a/APP cells.

In 2018, hybrids of THA with dihydropyirimidine-thiones **607**–**618** were reported [98]. Most tacripyrimidines showed selectivity for hBuChE with IC_50_ from 0.372 mM (**616**) to 154 mM (**614**). Additionally, most tacripyrimidines **607**–**618** inhibited hAChE with IC_50_ from 3.05 mM (**611**) to 31.0 mM (**615**). The most selective and potent hAChEI was **617** (hAChE: IC_50_ 0.0373 mM). All tacripyrimidines except **616** significantly inhibited Ca^2+^ influx induced by K-depolarization in SH-SY5Y cells. A hepatotoxicity study revealed most tacripyrimidines to be similarly or slightly less toxic than THA. Finally, **611** was chosen as a well-balanced inhibitor of ChEs and a calcium channel blocker, with no toxicity toward HepG2 cells up to 300 mM and excellent predicted oral absorption and BBB permeability.

**Figure 24:**
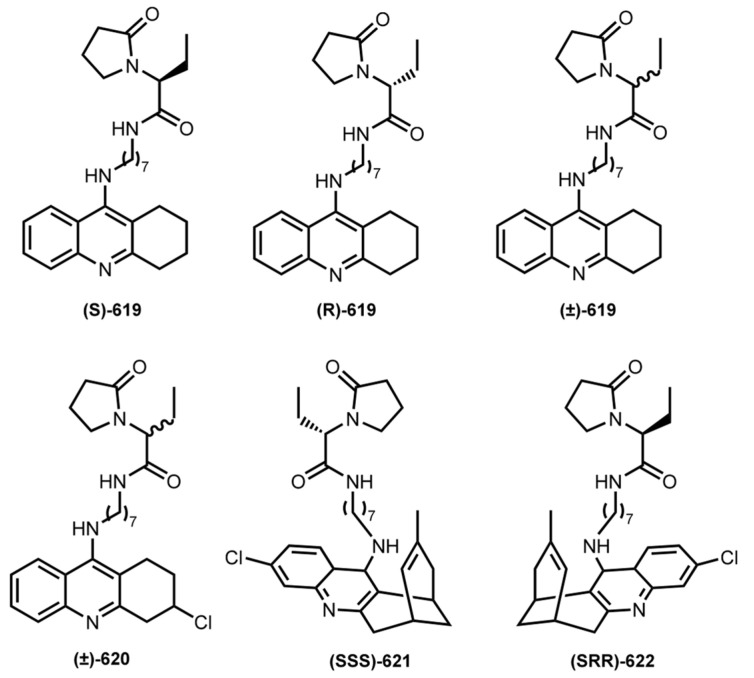
THA **(S)-619**, **(R)-619**, **(±)-619**, 6-Cl-THA **(±)-620**, huprine Y **(SSS)-621**, and **(SRR)-622**–based hybrids with 2-(2-oxopyrrolidin-1-yl)butyramide moiety of levetiracetam [99].

In 2015, Sola et al. reported hybrids **619**–**622** based on a THA, 6-Cl-THA, or huprine Y with 2-(2-oxopyrrolidin-1-yl)butyramide moiety of levetiracetam, an antiepileptic drug [99] that was reported to improve memory performance in mouse models of AD [187] (Figure 24).

All hybrids were potent inhibitors of hAChE, with IC_50_ in the low nanomolar range. Huprine is a stereoactive drug, with (7R,11R)-huprine derivatives being more potent hBuChE inhibitors than (7S,11S)-counterparts [188]. Expectedly, **621** and **622** showed different inhibition activity. However, no differences in inhibitory activity of (7S,11S)-huprine Y hybrids were detected. Hybrids **621** and **622** exhibited a moderately potent Aβ_42_ and tau antiaggregating activity. The inhibition of mouse brain AChE after i.p. administration of the levetiracetam-based hybrids was also confirmed. A significant reduction in the frequency of spontaneous convulsions in APP/PS1 mice treated with the levetiracetam-huprine hybrid **621** was revealed. APP/PS1 mice treated with hybrids **619** and **621** exhibited significantly increased recognition indices when compared to vehicle-treated animals. In addition, immunohistochemical determination revealed a reduction of the Aβ burden in the cortex of APP/PS1 mice after chronic treatment with **621**. Finally, chronic treatment with **621** led to a significant reduction of GFAP positive astrocytes around Aβ plaques and Iba1 positive microglial cells in APP/PS1 mice.

## 6. Tacrine Hybrids with Modulators of Cholinergic/Serotonergic System

### 6.1. Tacrine Hybrids with Modulators of Serotonin Receptors

**Figure 25:**
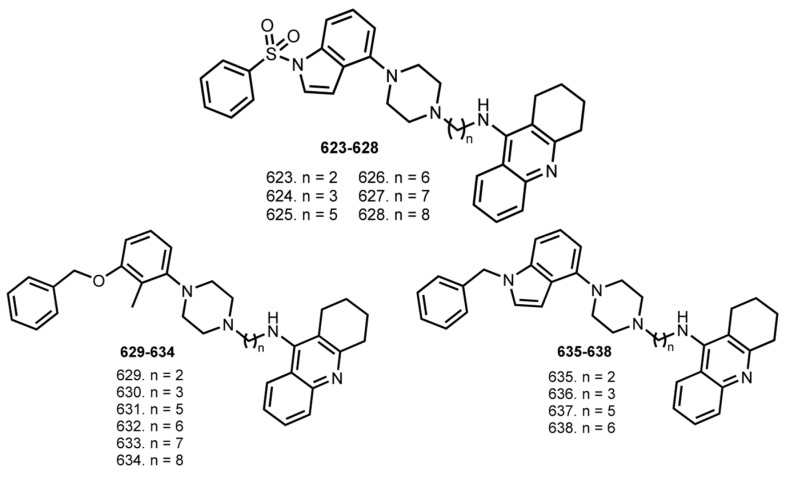
THA-1-(phenylsulfonyl)-4-(piperazin-1-yl)-1H-indole hybrids **623**–**628** [100], THA–5HT6-agonist hybrids **629**–**638** [101].

In 2016, Wigockowska et al. designed hybrids **623**–**628** as potential ChE inhibitors and 5-HT_6_ antagonists. As a 5-HT_6_ antagonist, 1-(phenylsulfonyl)-4-(piperazin-1-yl)-1H-indole was chosen (Figure 25) [100].

All hybrids displayed high affinities for 5-HT_6_ receptor in the low nanomolar range. IC_50_ values for hybrids were in the range from 7.1 to 57.0 nM for AChE and from 8.2 to 21.3 nM for BuChE. Hybrid **626** was chosen for its balanced activity as lead compound based on a set of test results. Hybrid **626** significantly diminished serotonin-induced calcium mobilization, confirming its 5-HT_6_ antagonistic properties (Kb = 27.0 nM). A kinetic study revealed a non-competitive type of inhibition of AChE/BuChE. Hybrid **626** decreased the rats’ locomotion activity and reduced scopolamine-induced hyperlocomotion in rats.

In 2018, an expanded library of similar hybrids **629**–**638** was synthesized [101]. The affinity of hybrids for recombinant human 5-HT_6_ receptor was found to increase with the linker length and reached a Ki value of 18 nM for **632**, which was confirmed by a radioligand binding assay. All hybrids were potent ChEs inhibitors, with the most active being **634** (IC_50_ 50 nM against AChE) and **632** (IC_50_ 14 nM against hAChE). A kinetic study revealed a non-competitive mode of action for **632**. Further, the inhibitory effect on Aβ aggregation was determined by ThT assay, and the most active hybrids were **632**, **633**, **635**–**638**. A PAMPA-BBB test showed a possible effective CNS permeability of hybrids. An in vitro metabolic stability study on human liver microsomes did not detect any hepatotoxic metabolites.

**Figure 26:**
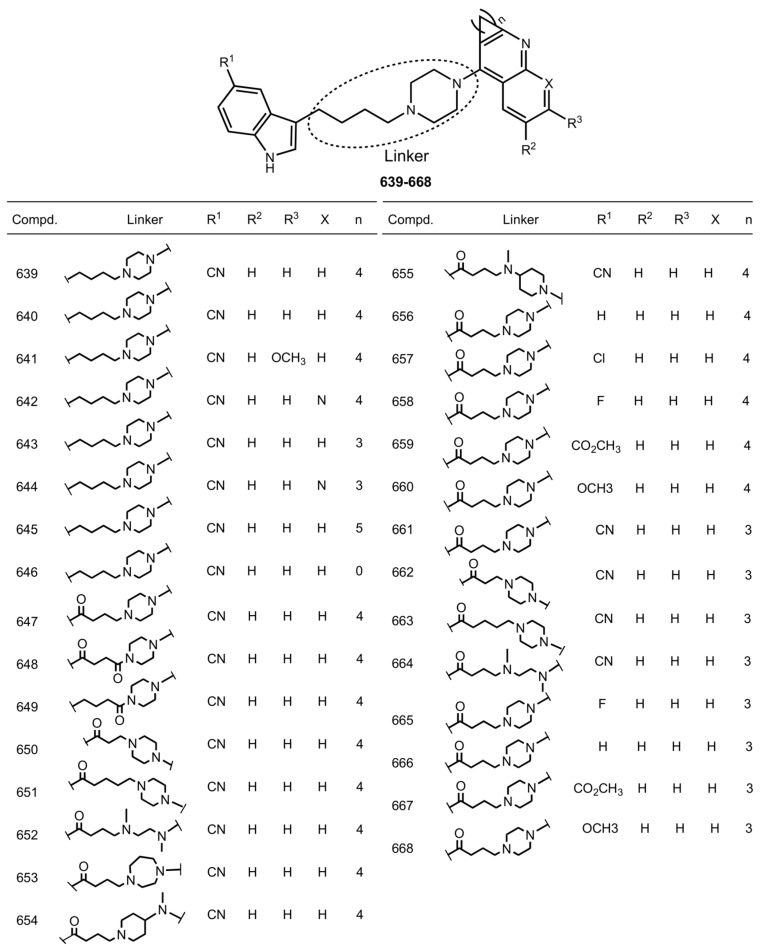
THA–Vilazodone hybrids **639**–**668** [102].

In 2017, Li et al. reported hybrids of THA with Vilazodone **639**–**668**, an inhibitor of serotonin reuptake and partial agonist of 5-HT_1_A receptor [102,189] (Figure 26). 

Activities of hybrids such as 5-HT_1_A agonists and 5-HT reuptake inhibitors were evaluated; hybrid **643** showed relatively balanced activities against the three targets. Low hepatotoxicity of **643** was confirmed on HepG2 cells. Low cardiotoxicity of **643** was confirmed by hERG activity inhibition. The antidepressive effect of **643** was confirmed by the tail suspension test in vivo, and cognitive improvements were detected in scopolamine-treated mice. 

### 6.2. Tacrine Hybrids with Modulator of Muscarinic Receptors

**Figure 27:**
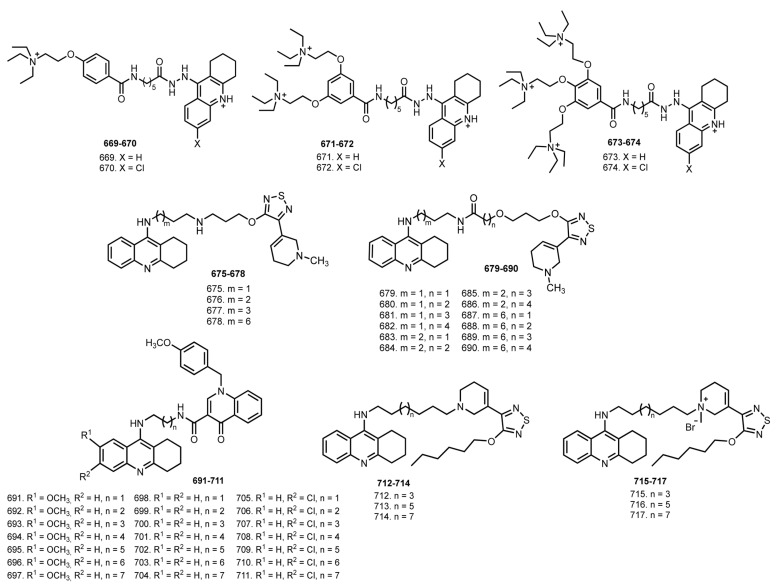
Gallamine–THA hybrids **669**–**674** [103], THA–xanomeline hybrids with amine linker **675**–**678** and amide linker **679**–**690** [104], 7-MEOTA–BQCA hybrids **691**–**697**, THA–BQCA hybrids **698**–**704**, 6-Cl-THA–BQCA hybrids **705**–**711** [105], and THA–xanomeline hybrids **712**–**717** [106].

In 2007, Elsinghorst et al. reported THA-based hybrids **669**–**674** with gallamine, an archetypal muscarinic allosteric agent [103] (Figure 27). An increasing substitution of the gallamine-derived moiety tends to reduce the inhibitory potency. Hybrid **669** was found to be a potent inhibitor of AChE (IC_50_ 500 pM). The interaction of the building blocks and hybrids **669**–**674** with M2 receptors was measured in receptors whose acetylcholine binding site was blocked by the radioligand [^3^H]NMS. Muscarinic allosteric ligands typically retard the dissociation of [^3^H]NMS by allosteric binding to [^3^H]NMS-occupied receptors, thereby prolonging the incubation time needed for reaching [^3^H]NMS equilibrium binding. All hybrids restrict [^3^H]NMS dissociation. Finally, hybrids showed an increase in the allosteric potency by factors of 100 relative to gallamine and 4800 relative to THA.

In 2010, Fang et al. reported THA-xanomeline hybrids **675**–**790** [104]. Xanomeline is an M1 activator, an M1/M4-preferring orthosteric agonist with antidementive properties in vivo [190]. All compounds were potent inhibitors of both cholinesterases. The most potent compound was **690** with pIC_50_ 8.21 against eeAChE. The affinity of hybrids for unliganded receptors was determined using the orthosteric radioligand [^3^H]N-methylscopolamine ([^3^H]NMS). All hybrids induced an allosteric inhibition of [^3^H]NMS dissociation. The most potent log K_Xdiss_ of **680** was more than three log units higher compared to xanomeline. In vivo studies in rats revealed the ability of **687** to significantly enhance scopolamine action.

In 2018, Hepnarova et al. reported hybrids of THA **691**–**711** with benzylquinolone carboxylic acid (BQCA; 1-(4-methoxybenzyl)-4-oxo-1,4-dihydroquinoline-3-carboxylic acid), a selective positive allosteric modulator of M1 mAChRs which does not interact with the Ach site with pro-cognitive action [105,191,192]. 7-MEOTA-based hybrids **691**–**697**, THA-based hybrids **698**–**704** and 6-Cl-THA-based hybrids **705**–**711** were potent inhibitors of cholinesterases. The most active hAChE inhibitors in each family were found as follows; **696**, **699**, and **706** with IC_50_ 1.5 µM, 0.13 µM, and 42 nM from each subset. Unfortunately, all hybrids exerted an antagonistic profile of M1 mAChR, instead of the expected agonostic profile. 

In 2020, Maspero et al. reported THA hybrids **712**–**717** with xanomeline, a selective muscarinic acetylcholine receptor agonist and M1/M4 preferring muscarinic acetylcholine receptor activator [106,193]. Hybrids **712**–**717** were able to inhibit AChE; eight methylene units were optimal for the highest AChE inhibition. The most active inhibitor was **715** with pIC_50_ 9.55. However, hybrids **712**–**717** were unable to activate the M1 receptor subtype.

### 6.3. Tacrine Hybrids with Cannabinoid CB1 Receptor Antagonists

Cannabinoid signaling systems are involved in a variety of physiological processes. The selective CB1 antagonist/inverse agonist drug Rimonabant is an FDA-approved drug to treat obesity and metabolic-related disorders [194]. In 2007, Wise et al. reported [195] a combination of rimonabant and donezepil, a CB1 antagonist and an AChE inhibitor, as an effective memory-enhanced therapy. 

**Figure 28:**
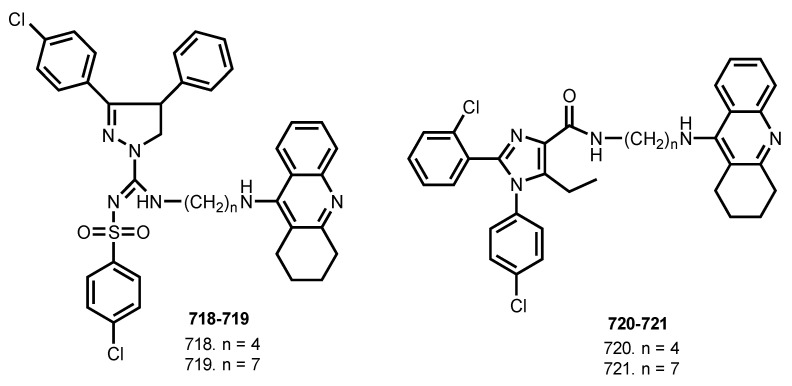
AChEIs/CB1 receptor antagonists **718**–**721** [107].

In 2010, Lange et al. reported hybrids of THA with cannabinoid CB1 receptor antagonists **718**–**721** [107] (Figure 28).

Hybrids exhibited AChE inhibiting activities and significant cannabinoid CB1 receptor antagonistic properties. The most effective AchE inhibitor was **720** (pIC_50_ 6.5). Hybrids **718**–**721** showed significant CB1 receptor affinities and, in general, acted as CB1 receptor antagonists, while **720** showed significant CB1 receptor affinity with Ki = 48 nM.

### 6.4. Tacrine Hybrids with Modulator of NMDA Receptors

**Figure 29:**
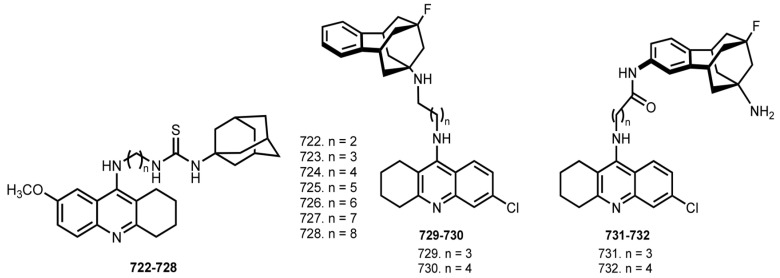
7-MEOTA–adamantylamine hybrids **722**–**728** [108], benzohomoadamantane–6-Cl-THA hybrids **729**–**732** [109].

In 2013, Spilovska et al. designed 7-MEOTA-adamantylamine thioureas **722**–**728** [108] (Figure 29).

All hybrids exhibited good inhibitory activity toward ChEs. The most potent cholinesterase inhibitor was **725**, with an IC_50_ 0.47 µM for hAChE and 0.11 µM for hBuChE.

In 2019, Perez-Areales et al. designed benzohomoadamantane -6-Cl-THA hybrids **729**–**732** with unsubstituted amino groups [109]. All hybrids were potent hAChE inhibitors, 6- to 44-fold more potent than 6-Cl-THA. Hybrid **731** was the most potent hAChE inhibitor. The most potent hBuChE inhibitors were hybrids **730** and **732** (IC_50_ 210 and 21 nM).

When the effects of hybrids on the increase in intracellular calcium evoked by NMDA in neurons loaded with Fura-2 was evaluated [196], the most potent NMDA antagonists were **730** and **731**. Unfortunately, low BBB permeation for **731** and **732** was predicted, whereas when substituted at the bridgehead amino group **729** and **730** were predicted to be able to cross the BBB.

### 6.5. Tacrine Hybrids with Modulators of Opioid Receptors

**Figure 30:**
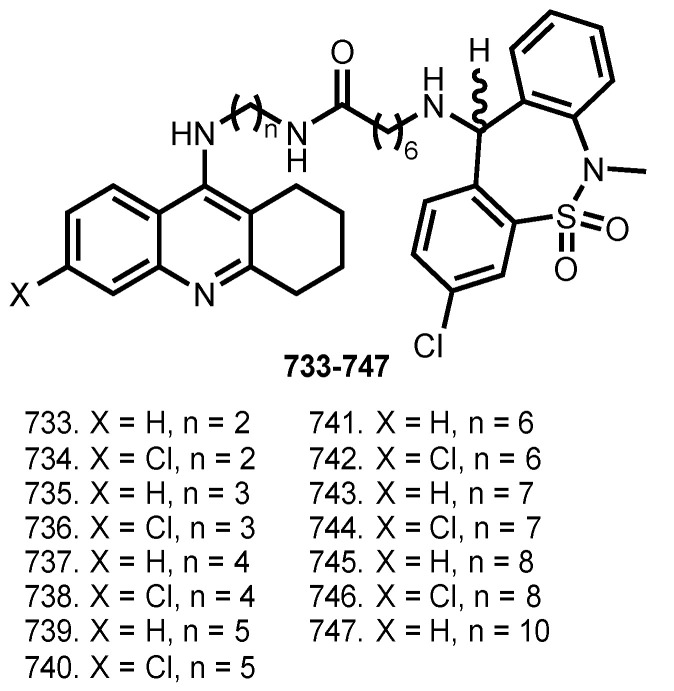
THA–Tianeptine hybrids **733**–**747** [110].

In 2016, Ceschi et al. reported hybrids of THA with antidepressant Tianeptine **733**–**747** [110] (Figure 30).

THA-tianeptine hybrids were potent inhibitors of cholinesterases, the most active AChE inhibitor was **736** (IC_50_ 6.79 nM), and **737** was the most active and selective in inhibiting BuChE (IC_50_ 3.59 nM). Molecular modeling studies showed that THA moiety targets CAS, while tianeptine binds to PAS. Additionally, **737** and **739** were able to reduce the in vitro basal secretion of S100B, a calcium-binding protein which is known to regulate several processes associated with AD [197].

### 6.6. Tacrine Hybrids with MAO Inhibitors

MAO inhibitors (namely, two isoforms MAO-A and MAO-B) are considered as promising therapeutic agents for AD [198,199,200]. Ladostigil is a drug with cholinesterase and brain-selective monoamine oxidase inhibitory activities approved for phase IIb clinical trial [189].

**Figure 31:**
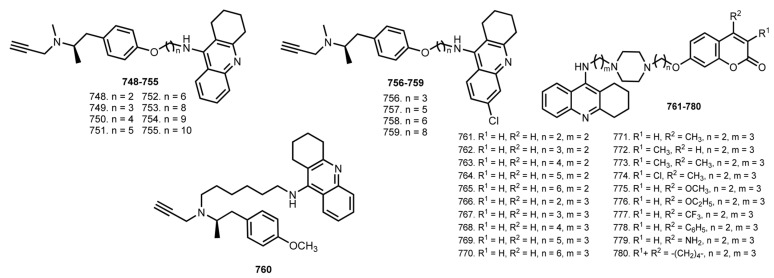
THA–selegiline hybrids **748**–**760** [111], THA–coumarin hybrids **761**–**780** [112].

In 2013, Lu et al. reported a number of THA-based hybrids with drug Selegiline, a selective inhibitor of MAO-B [111,201]. (Figure 31) 

Hybrids **748**–**751** were inhibitors of AChE, with the most active being **749** (IC_50_ 36.1 nM). In addition, **755** exhibited the best IC_50_ toward BuChE in 2.03 nM. Hybrid **754** was chosen as lead due to balanced activity based on a set of test results. A kinetic study revealed a mixed-type inhibitory behavior for **754**. Most of the hybrids were effective in inhibiting MAO-A and MAO-B in the sub-micromolar range. Hybrid **759** showed the highest inhibitory activity for both MAO-A (IC_50_ 0.1926 mM) and MAO-B (IC_50_ 0.1290 mM), and **754** exhibited the best balance of inhibition for both ChE and MAO. Finally, **754** proved to be an irreversible MAO-B inhibitor.

In 2015, Xie et al. designed THA-coumarin hybrids **761**–**780** [112]. Coumarin moiety was chosen due to its MAO inhibitory activity [202] and AChE inhibitory activity [203]. Hybrid **766** gave the highest AChE inhibitory activity with IC_50_ 17.70 nM. The substituents in coumarin moiety were found to worsen the inhibitory activity. Hybrids **774** (IC_50_ 31.88 nM for AChE) and **771** (IC_50_ 50.76 nM for BuChE) were the most potent inhibitors, with their inhibitory activity 1.8- and 1.3-fold less than those of their no substituted analog **766**. All hybrids showed inhibition activity against hMAO-A and hMAO-B, with the most selective toward MAO-B being **773** (IC_50_ 0.24 mM). An inhibitory activity of **773** as a mixed-type competitive inhibitor was confirmed. Finally, **773** showed negligible toxicity on SH-SY5Y cells.

## 7. Tacrine Hybrids with Natural Products

**Figure 32:**
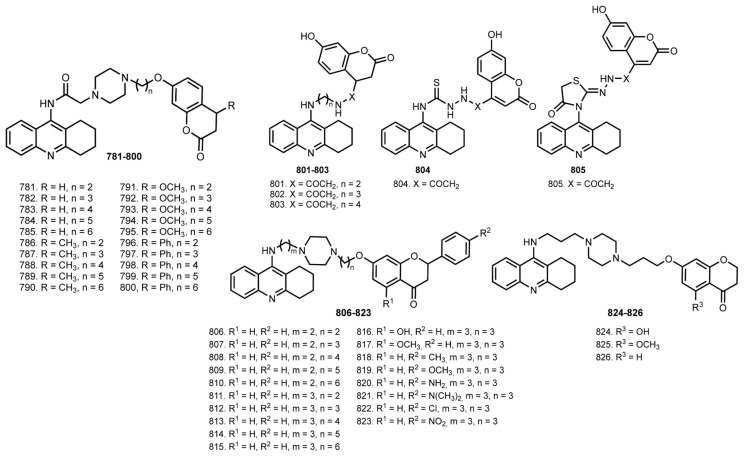
THA–coumarin hybrids **781**–**800** [113], **801**–**805** [114], and THA–flavonoid hybrids **806**–**826** [115].

In 2013, Xie et al. reported THA–coumarin hybrids **781**–**800**, structural analogues of hybrids **761**–**780** (Figure 32) [112,113]. Hybrids showed moderate activity toward both ChEs, with the best AChE inhibitor being **786** (IC_50_ 0.092 µM), and the most effective BuChE inhibitor being **790** (IC_50_ 0.099 µM). Kinetic study revealed a mixed-type inhibition for **786**. An ability of hybrids to inhibit self-induced Aβ aggregation was confirmed using a ThT-test, with **786** as the most potent AChE inhibitor that also showed the highest inhibitory potency. The metal-chelating ability of hybrids was confirmed using UV-Vis spectrometry in the presence of Cu^2+^ and Fe^2+^. Finally, a low toxicity of **786** was confirmed on SH-SY5Y cells.

In 2014, Hamulakova et al. reported THA–coumarin hybrids **801**–**805** [114]. The most potent inhibitor of hAChE was **803** (IC_50_ 0.0154 μM). A selectivity for hAChE was demonstrated by **803** (SI 21.30) and for hBuChE by **804** (SI 0.174).

In 2013, Li et al. reported THA–flavonoid hybrids **806**–**826** [115]. All hybrids inhibited both ChE, with the most potent being **825** (IC_50_ 8.4 nM toward AChE) and **826** (IC_50_ 25.8 nM toward BuChE). Further, most hybrids inhibited Aβ self-induced aggregation, with the most potent being **824**. Hybrids **816** and **824** showed moderate metal-chelating ability. Additionally, **816** was non-toxic to SH-SY5Y cells.

**Figure 33:**
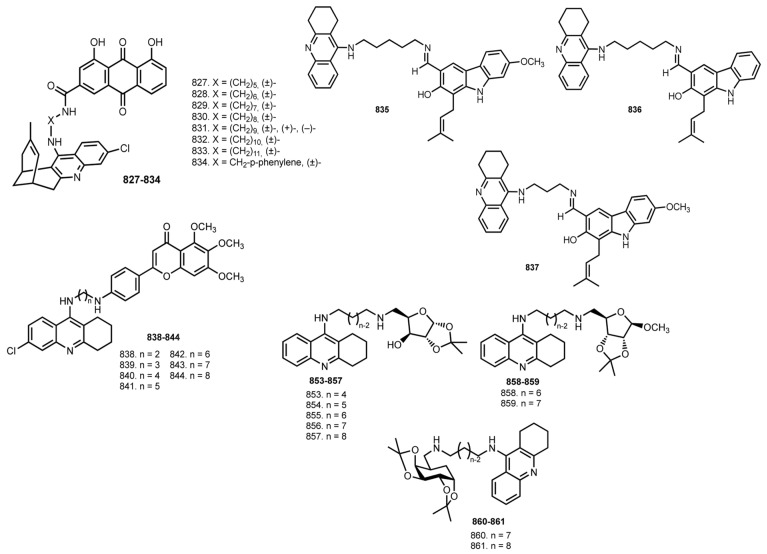
Huprine Y-rhein hybrids **827**-**834** [116], THA-carbazoles hybrids **835**–**837** [117], 6-Cl-THA–Scutellarin hybrids **838**–**844** [118], THA–resveratrol hybrids **845**–**852** [119], THA hybrids with natural-based D-xylose, D-ribose, and and D-galactose **853**–**861** [120].

In 2014, Viayna et al. reported huprine Y-rhein hybrids **827**–**834** [116] (Figure 33). All racemic hybrids were potent inhibitors of hAChE, with IC_50_ values in the low nanomolar range. The most potent hAChE inhibitor was **(±)-827** (IC_50_ 1.07 nM). In addition, all hybrids were selective for hAChE. The binding mode to AChE was explored for **834** via docking studies, in which **(−)-834** bound to AChE more favorably than did **(+)-834**. For all hybrids, a significant Aβ_42_ antiaggregating activity was confirmed. Additionally, **(±)-830**, **(±)-832**, and **(±)-833**, as well as **(±)-834**, exhibited a moderately potent BACE-1 inhibitory activity. The levorotatory **(−)-831** was a far more potent hAChE inhibitor than its enantiomer **(+)-831**, with IC_50_ 2930 and 2.39 nM. A kinetic study demonstrated that **(−)-831** acts as a mixed-type inhibitor of hAChE. **(+)-831** was two-fold more potent hBuChE inhibitor than **(−)-831**. In addition, both **(−)-831** and **(+)-831** proved to prevent the loss of synaptic proteins in hippocampal slices of 2-month-old C57bl6 mice. In vivo experiments with transgenic APP-PS1 mice showed that **(+)-** and **(−)-831** can lower the levels of hippocampal total soluble Aβ and increase the levels of APP.

In 2014, Thiratmatrakul et al. reported hybrids of THA with phytochemicals carbazoles **835**–**837** [117]. All hybrids showed potent ABTS radical scavenging capacities with IC_50_ in the range of 8.34–11.24 µM, and selectivity against AChE over BuChE. Hybrid **835** displayed the most potent inhibitory activity and inhibition selectivity toward AChE, (IC_50_ 0.48 µM). A neuroprotective effect of hybrids against H_2_O_2_ -induced oxidative stress was shown on NG108–15cells and **835** proved to be most potent in protecting cell damage. Additionally, neuroprotective effect of hybrids against Aβ peptide induced toxicity was shown on C6 astroglioma cells. Hybrid **835** was also the most potent in increasing cell viability. Behavioral studies indicated that **835** could improve scopolamine-induced cognitive deficits in mice. 

In 2017, Spilovska et al. reported THA–scutellarin hybrids **838**–**844** [118]. The most active was **838** (IC_50_ 1.63 nM against AChE) and the most potent inhibitor of hBuChE was **839** (IC_50_ 174 nM). Only **843** and **844** showed lower cytotoxicity compared to the 6-Cl-THA.

In 2017, Jeřábek et al. reported THA-resveratrol hybrids **845**–**852** [119]. The most potent AChE inhibitor was **845** (IC_50_ 0.8 µM). Some antiaggregating properties of hybrids were revealed by ThT assay. Only **852** showed no neurotoxicity on primary rat cerebellar granule neurons (CGNs). Nitrite production in LPS-treated glial cells was evaluated, which was significantly reduced by treatment of cells with **852**. Finally, an ability of **852** to modulate the switch from the M1 to M2 phenotype on glial cells was investigated; a decrease in iNOS and slightly attenuating MRC1 expression was detected. Unfortunately, hepatotoxicity of hybrids on HePG2 cells was shown. 

In 2018, Lopes et al. reported THA hybrids with natural-based D-xylose, D-ribose and D-galactose **853**–**861** [120]. Hybrid **857** showed an IC_50_ 2.2 nM against AChE and of 4.93 nM against BuChE. Docking studies revealed that sugar moieties are stabilized in the PAS region through cation-π and CH/π interactions with Trp279.

**Figure 34:**
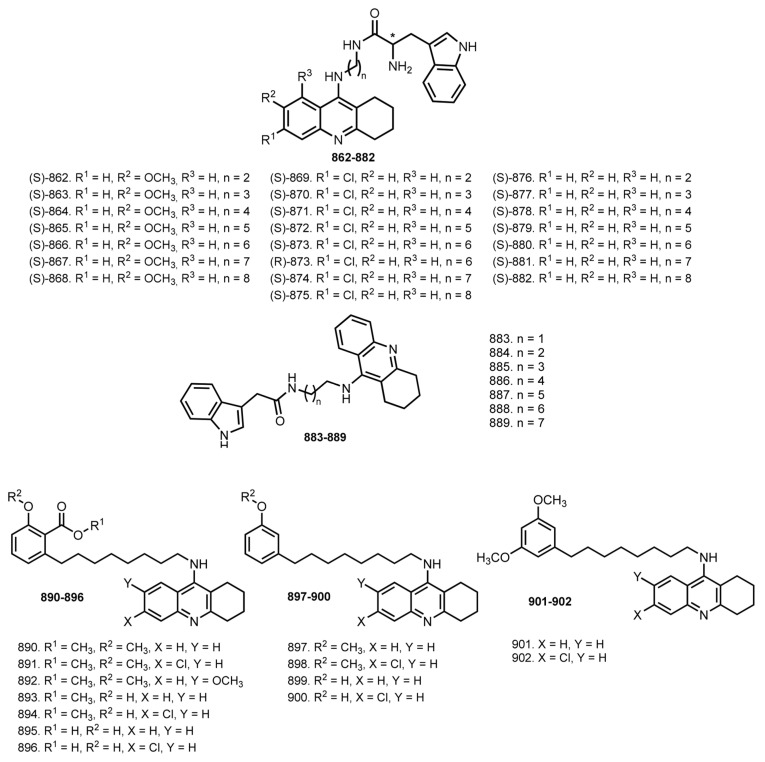
THA–tryptophan hybrids **862**–**882** [121], THA–indole hybrids **883**–**889** [122], THA-based hybrids with anacardic acid **890**–**896**, cardanol **897**–**900**, and cardols **901** and **902** [123].

In 2019, Chalupova et al. designed THA–tryptophan heterodimers **862**–**882** [121] (Figure 34). All hybrids (except **(S)-864**) were potent inhibitors of hAChE; **(S)-873** (IC_50_ 6.3 nM) was chosen as lead compound based on a set of test results. S-enantiomer was found to be 15-fold more potent than the R- (9.1 nM vs. 140 nM). Moreover, the crystal structure confirmed the ability of **(S)-873** to target both the CAS and PAS of AChE. PI displacement studies showed that the interaction of **(S)-873** with PAS was about 6.9-fold weaker than that of PI. All hybrids were able to significantly inhibit Aβ_42_ -self-aggregation. 

Cell toxicity studies revealed the order of toxicity is as follows: THA derivatives < 7-MEOTA analogues < 6-Cl-THA derivatives. The maximum tolerated dose of **(S)-873** was found to be 70 mg/kg, meaning **(S)-873** is safer than THA. The therapeutic effect of **(S)-873** in a scopolamine-induced cognitive deficit rat model confirmed the pro-cognitive potential of the hybrid.

In 2019, Cheng et al. reported THA–indole hybrids **883**–**889** [122]. A moderate inhibition activity was shown by all hybrids. Hybrid **887** with IC_50_ 0.173 μM against AChE was chosen as lead based on a set of test results. Kinetic study that revealed **887** is a mixed type ChE inhibitor. In addition, **887** exhibits a much stronger effect in modulating neural network activity compared to THA, indicating better antidementia and nootropic potentials. 

In 2021, Rossi et al. reported THA-based hybrids conjugated with anacardic acid, cardanol, and cardols **890**–**902** [123]. All hybrids were effective AChE inhibitors, with the most active being **891** (IC_50_ 2.54 nM). As BuChE inhibitors, **890**, **891**, and **893** were the top-ranked with IC_50_ 0.0352 nM, 0.265 nM, and 0.177 nM. A crystal structure of **890** with hBuChE revealed that **890** accommodates the active site gorge of hBuChE. Toxicity studies revealed low hepato- and neurotoxicity in hybrids. Anti-inflammatory activity of **890** and **891** was shown on LPS-treated microglial BV-2 cells; a protective activity against neurotoxic insults was detected, as was the suppression of LPS-induced IL-1β, COX-2, and iNOS expression (TNF-α only for **891**). The PAMPA-BBB test predicted that both hybrids have the potential to cross the BBB.

## 8. Tacrine Hybrids with Other Organic Scaffolds

**Figure 35:**
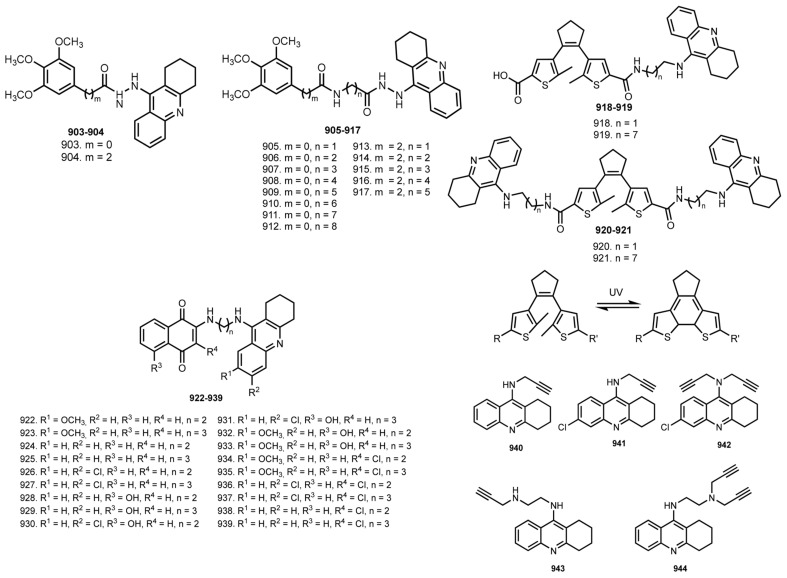
THA-trimethoxybenzene hybrids **903**–**917** [124], photoswitchable hybrids **918**–**921** [125], quinone–THA hybrids **922**–**939** [126], and THA–propargylamine hybrids **940**–**944** [127].

In 2006, Elsinghorst et al. reported THA hybrids with trimethoxybenzene **903**–**917** [124] (Figure 35). The most active inhibitor of hAChE was **912** (IC_50_ 5 nM). In addition, several hybrids showed selectivity toward hBuChE, with the most active inhibitor being **916** (IC_50_ 0.139 nM).

In 2014, Chen et al. reported a first example of photoswitchable THA-based hybrids [125]. Hybrids **918**–**921** showed reversible light-controlled behavior. The absorbances around 270 nm decreased upon UV-irradiation, whereas the absorbance band at 340 nm increased, and a new absorbance band maximum at 525 nm evolved. However, in all cases, the inhibitory activity against AChE was practically the same before and after irradiation of hybrids, except for the **921** (IC_50_ 4.3 nM for “open” molecule and 1.8 nM for “closed’). Computational docking studies suggest that **921** might bind to both CAS and the PAS of AChE in both opened and closed forms. Additionally, when AChE-induced Aβ aggregation under the action of both photoforms of **921** were investigated, ring-closed **921** showed lower inhibition than a ring-open form. 

In 2014, Nepovimova et al. designed quinone-THA hybrids **922**–**939** [126]. All hybrids were effective inhibitors of hAChE, with **930** being 752-fold more active in inhibiting hAChE than hBuChE. Hybrids **922**–**939** significantly prevented self-induced Aβ aggregation. Hybrids **926** and **930** were least neurotoxic on N2A cells. A neuroprotective effect of **926** and **930** was shown on N2A cells incubated with Aβ. The antioxidant properties of **926** and **930** were confirmed on TBH-stressed T67 cells pre-treated with sulforaphane. In addition, AChE inhibition was also confirmed ex vivo. A percentage of brain AChE inhibition versus untreated controls was evaluated after the drug’s injection; both **926** and **930** provide dose-dependent inhibition of cholinesterase activity in telencephalon at 1 h postdosing. Finally, **926** showed a superior hepatotoxicity profile to THA.

In 2015, Mao et al. reported THA–propargylamine hybrids **940**–**944** [127]. Hybrid **941** showed good inhibition activity for both ChEs (IC_50_ 11.2 and 83.5 nM). Additionally, a mixed-type inhibitory behavior for **940** and **941** was revealed. Hybrid **940** showed the absence of neurotoxicity on SH-SY5Y cells. Finally, **940** and **941** showed low hepatotoxicity on HSC.

**Figure 36:**
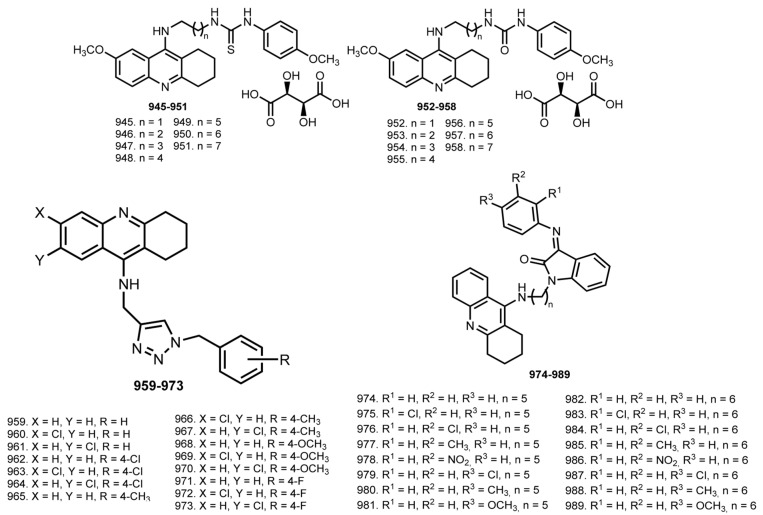
7-MEOTA-p-anisidine hybrids **945**–**958** [128], THA-1,2,3-triazole hybrids **959**–**973** [129], Schiff base hybrids **974**–**989** [130].

In 2015, Korabecny et al. designed 7-MEOTA hybrids **945**–**958** with p-anisidine [128] (Figure 36). All hybrids turned out to be potent inhibitors, but **955** showed the best inhibiting activity (IC_50_ 1.35 µm against hAChE,) and **951** showed IC_50_ 1.36 µm against hAChE. Kinetic analysis revealed a non-competitive type of inhibition of AChE by **951** and **955**. In silico studies confirmed the dual binding site character of the selected ligands, with prevailing interactions with the PAS region of hAChE. 

In 2017, Najaf et al. reported THA–1,2,3-triazole hybrids **959**–**973** [129]. All hybrids showed moderate inhibition activity against ChEs, with the best inhibitor being **970** (IC_50_ 0.521 mM); **968** was the best BuChE inhibitor (IC_50_ 0.055 mM). Kinetic study confirmed mixed types of inhibition for both AChE and BuChE. In vivo evaluation of **970** confirmed memory improvement in scopolamine-induced impairment.

In 2019, Riazimontazer et al. reported THA–isatin schiff base hybrids **974**–**989** [130]. Most of the hybrids were potent ChE inhibitors with IC_50_ values from 0.42 nM to 79.66 nM. The most active, **984**, showed IC_50_ against AChE 0.42 nM. Hybrid **977** exhibited the strongest inhibition of BuChE with IC_50_ 0.11 nM. Kinetic study of AchE inhibition revealed a mixed-type inhibition for **984**. In addition, **984** and **986** exhibit good inhibitory activity on AchE-induced Aβ aggregation. Metal-chelating properties for **986**, **984**, and **989** were shown.

**Figure 37:**
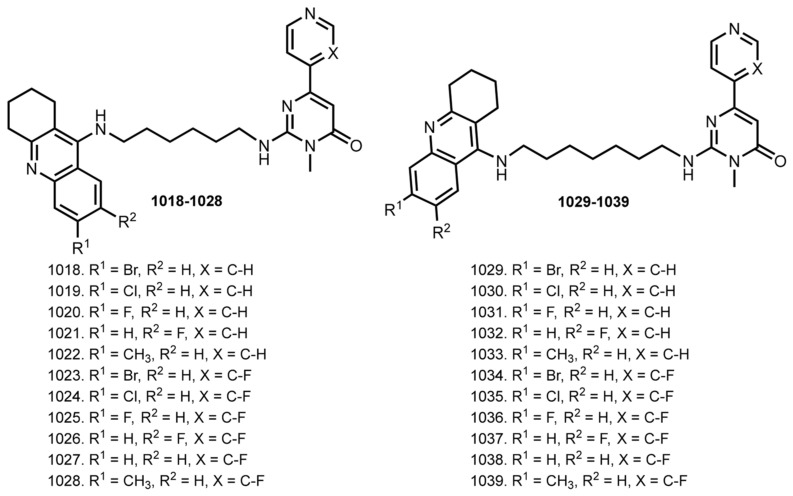
THA—pyrimidone hybrids **990**–**1039** [131].

In 2021, Yao et al. reported THA−pyrimidone hybrids **990**–**1039** [131] (Figure 37).

Br- and Cl- as substituents in THA unit were found to enhance AChE inhibition, fluorine-substituted pyridine groups were found to intensify to GSK-3β target, and alkylamine linkers with a linear chain of seven carbons were chosen as the most beneficial moiety. Hybrid **1035** was chosen as the compound with excellent dual AChE/GSK-3 inhibition (AChE: IC_50_ 51.1 nM; GSK/3β: IC_50_ 89.3 nM).

The docking studies for **1035** proved both CAS and PAS binding. Hybrid **1035** could fit the binding pockets of AChE and GSK-3β and exhibited good affinity with the interactions of several secondary bonds through the cooperation of the THA unit, alkylamine linker, and pyrimidone moiety, making it an excellent dual AChE/GSK-3β inhibitor. Additionally, **1035** was proven to regulate the tau protein pathway in SH-SY5Y-derived neurons, and to alleviate glyceraldehyde-induced cytotoxicity in DSH-SY5Y cells. A kinase selectivity profiling study showed that **1035** is a pan-GSK-3 inhibitor and possessed good kinase selectivity profiles. The capacity of **1035** to successfully permeate the BBB was confirmed by UPLC-MS/MS. Finally, treatment of scopolamine-induced ICR mice with **1035** led to significant amelioration of memory and spatial behavior. 

**Figure 38:**
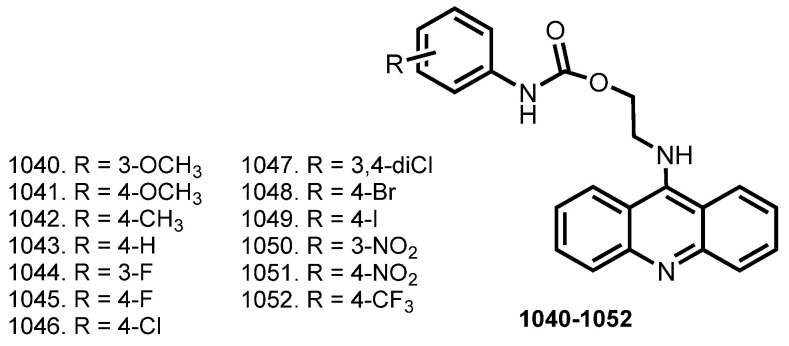
THA–carbamate hybrids **1040**–**1052** [132].

In 2021, Ozten et al. reported carbamate hybrids of THA **1040**–**1052** [132] (Figure 38). 

All hybrids inhibited both ChEs, but **1050** was chosen as the best inhibitor of AChE and BuChE (IC_50_ 22.15 nM and 16.96 nM).

**Figure 39:**
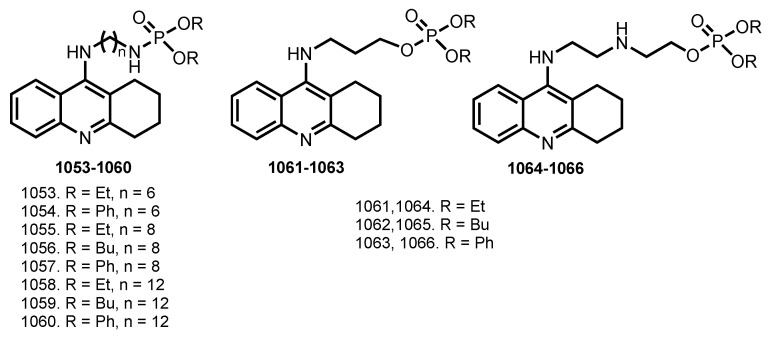
THA hybrids with phosphorus moieties **1053**–**1066** [133].

In 2022, Przybyłowska et al. reported THA hybrids **1053**–**1066** with phosphorus moiety [133] (Figure 39).

Hybrids were mostly more neurocytoxic than THA. Only **1064** showed a significant reduction of hepatotoxicity against HepG2 cells when compared with THA. In ChEs test inhibition, **1053** and **1058** showed similar activity with THA. The most active hybrid was **1055** with IC_50_ 6.11 nM against AChE and 12.86 nM against BuChE. In addition, **1060** and **1053** were potent against BuChE, with IC_50_ 1.969 nM and 6.753 nM. 

## 9. Discussion

In summary, the most effective inhibitors of hAChE among those presented in this review are **7** and **565**, which are hybrids of 6,8-dichlorotacrin-melatonin (**7**) and 6-chlorotacrin-tetrahydrobenzo[h][1,6]naphthyridine (**565**) with IC_50_ 8 and 6 pm [39,93]. Additionally, hybrids with picomolar efficacy are 6-chlorotacrine-4-oxo-4H-chromene **88** with IC_50_ 35 pm against hAChE and **74** with IC_50_ 38 pm against hBuChE [45]. THA hybrid with anacardic acid **890** also showed picomolar activity against hBuChE with IC_50_ 35 pm [123]. 

THA–melatonin hybrid **7**, first described in 2006, is still one of the most potent hybrids described to date. At the same time, THA–melatonin hybrids **3** and **6** showed selectivity for one esterase, which also makes them promising scaffolds for the development of anti-AD drugs. The conjugation of additional lipoic or ferulic acid moieties reduces inhibition activity (**24**–**31**), apparently due to steric hindrances arising in the resulting hybrid when binding to the target ChE [41].

THA–silibinin hybrid **101** demonstrated low hepatotoxicity in vivo, but was not superior to THA in improving cognitive abilities, presumably due to high steric hindrance [47]. Hybrid **165** showed both cognitive improvements and a decrease in hepatotoxicity in vivo, which clearly indicates the effectiveness of antioxidant strategy [54]. In addition, a reduction in Aβ plaque levels in the APP/PS1 mice certainly proves the effectiveness of simultaneous anti-amyloid and anti-AChE approaches in the treatment of AD.

An extremely interesting result was observed for hybrids of THA and FA. Thus, **254** with linker length n = 8 did not show cognitive improvement in vivo, while hydride **255** with linker length n = 6 showed significant cognitive improvements [51,52]. This once again emphasizes the importance of choosing the linker length both for the effectiveness of cholinesterase inhibition and for anti-AD therapy in vivo. It is also important to note that THA-FA hybrids **277**–**281** were linked through a conformationally rigid piperazine linker [62]. Comparison of the inhibition data of these hybrids with their analogues clearly indicates that the restriction of the conformational mobility of the linker negatively affects the inhibitory effect.

Also, the effectiveness of introducing NO-donors into THA hybrids for anti-AD therapeutic efficacy should be noted. Thus, a comparison of the in vivo efficacy of hybrids **262** (with the NO-donating group) and **256** (without the NO-donating group) clearly indicates that the vasorelaxant effect of NO-donors not only reduces the hepatotoxicity of hybrids, but also improves cognitive functions in vivo better than hybrids that do not possess a vasorelaxant effect [53]. Convincing evidence for the effectiveness of NO-group donors was also concluded from the results of in vivo studies of hybrids **295, 310**, and **317**. Hybrid **317** (without the NO-donating group) did not show improvement in cognitive functions in tests in vivo, while **295** and **310** showed improvements in cognitive functions, apparently due to the vasorelaxant effect [64,65]. It is also worth noting the THA–flurbiprofen hybrids **439**–**443** which were then optimized by conjugation with NO-donor molecules to give **444**–**463** [74,75,76]. At the same time, **456** did not outperform THA in improving its cognitive functions; however, the reduced hepatoxicity of **456** is attributed by the authors to its ability to donate NO. 

Despite the seeming simplicity of THA-linker-second residue hybrids synthesis, careful design is critical. Thus, among the THA–benzothiazole hybrids **359**–**364**, **370**–**395**, and **402**–**413** developed, only **386** showed high inhibitory activity along with low hepatotoxicity in vivo and therapeutic efficacy in vivo [68,69,70,72]. 

Not only the length of the linker is critical in drug design, but also its nature and donor properties. Thus, the amide linker between THA and benzofuran in **370**–**381** renders a negative effect on inhibitory activity compared to the amine linker in **382**–**387**. Additionally, both aromatic moieties in a hybrid should not be sterically expanded to be able to pack into the enzyme binding site. Thus, **388**–**395** show selectivity for BuChE, apparently due to the steric impossibility of binding to AChE. Regarding the negative effect of an amide linker on inhibition activity, **781**–**800** exhibited moderate anti-AD activity, while in subsequent work, similar hybrids **761**–**780** were designed not with an amide, but with an amine linker [112,113]. Optimization of the molecular structure resulted in an increase in anticholinesterase activity, and in the ability of **761**–**780** to inhibit MAO.

The exceptionally successful design of hybrids with analogues of PI deserves special attention. The known possibility of the second moiety to bind to the AChE provides a successful design of the anti-AChE drug, in which the optimal length of the linker between the CAS and PAS binding fragment should be selected. Thus, **565** is one of the most potent inhibitors of hAChE reported to date, with an IC_50_ 6.27 pM [92]. It is interesting to note that the design of **565** is also a result of careful drug development, as originally synthesized **555**–**564** were not effective inhibitors [91]. However, the successful design made it possible to obtain one of the strongest inhibitors described to date. In vivo studies on the efficacy of **565** would be extremely interesting.

Of obvious interest are hybrids acting on the cholinergic system. Thus, **592**–**605**, which are both cholinesterase inhibitors and calcium channel blockers, were developed and the leader compound **(S)-600** was chosen, of which studies of the in vivo therapeutic efficacy would be very interesting [95,96]. In addition, similar hybrids **607**–**618**, representing a weak structural analogue based on dihydropyirimidine-thiones, are also able to block calcium channels [98]. For **611**, the study of anti-AD activity for (R) and (S) enantiomers would certainly be interesting.

Hybrids **669**–**674** and **675**–**690** are all cholinesterase inhibitors and muscarinic receptor agonists; **687** shows therapeutic efficacy in vivo [103,104]. Both the hybrid series **675**–**690** and **712**–**717** are THA–xanomeline hybrids [104,106]. At the same time, the first series are successful activators of M1 receptors, while the second series are not able to affect receptors, which once again confirms the importance of not only the choice of the second moiety, but also the design of the linker. Additionally, THA–BOCA hybrids **691**–**711** turned out to be antagonists of muscarinic receptors, which confirms the complexity of receptor-targeted drug design [105]. The design of hybrids of THA with a memantine residue is a classic “dual drug” idea, while **722**–**728** do not show significant anticholinesterase activity [108]. At the same time, the change in the linker and the conjugation of the free amino group to the adamantane core significantly increased the activity of the resulting hybrids, and **729**–**732** turned out to be promising cholinesterase inhibitors and NMDA receptor antagonists [109]. Unfortunately, these hybrids are presumably unable to cross the BBB, possibly due to steric hindrance. Hybrids **759** and **773** are promising anticholinergic agents and MAO-B inhibitors, and their in vivo efficacies as anti-AD agents are also of interest [111,112].

Of particular note is the therapeutic efficacy of hybrids based on Huprine Y. Thus, Huprine Y–capsaicin hybrid **249** significantly enhanced learning and memory in old APP/PS1 mice [58]. Huprine-THA heterodimers **(±)-494** and **(±)-500** inhibited mouse brain AChE activity [85]. In addition, Huprine Y-based hybrid **(SSS)-621** with 2-(2-oxopyrrolidin-1-yl)butyramide moiety of levetiracetam significantly increased recognition indices in vivo, reduced Aβ burden in the cortex, and reduced GFAP positive astrocytes around Aβ plaques [99]. Additionally, both enantiomers of hybrid **(+)**- and **(−)-831** are able to lower the levels of hippocampal Aβ and increase the levels of APP both in initial and advanced stages of AD in vivo [116]. It should also be noted that the right and left hybrids **(+)** and **(−)-831** showed a tremendous difference in the inhibition of hAChE, and both showed anti-AD efficacy in vivo.

THA-carbazoles hydride **835** is not an effective cholinesterase inhibitor compared to the other hybrids described above, but demonstrated an ability to cause cognitive improvements in vivo [117]. As hybrids of THA with natural products, resveratrol **845**–**852** showed high hepatotoxicity [119]. THA hybrids with sugar moieties **853**–**861** and THA–anacardic acid hybrids **890**–**891** showed high efficacy along with low toxicity, and their further studies are promising [120,123]. An interesting result is the activity of the **(S)-873** enantiomer toward AChE [121].

The design of photoactive THA hybrids **918**–**921** is an interesting concept, but its practical applicability is questionable [125]. The spectacular work on the development of quinone–THA hybrids by Nepovimova et al., in which **926** is a selective AChE inhibitor, exhibits multiple activities and inhibits AChE in vivo [126]. Hybrids **940**–**944** with propargylamine as the second residue are striking in their simplicity and efficiency; further research on hybrids would be interesting [132]. 7-MEOTA-based hybrids with p-anisidine **945**–**958** are not as active, presumably due to the low activity of the parental 7-MEOTA [128]. Despite the fact that simple THA–triazole hybrids **959**–**973** are not active compared to other previously described hybrids, **970** showed therapeutic efficacy in vivo [129]. Hybrids of THA with isatin, especially **984**, also showed good activity [130]. An extremely interesting THA hybrid is pyrimidone **1035**, which exhibits AChE/GSK-3 inhibition activity and also exhibits therapeutic efficacy in vivo [131].

Comparison of structurally similar hybrids **67**–**94**, **149**–**153**, and **806**–**826** based on THA and flavonoids is interesting [45,52,115]. Thus, **67**–**94** are able to inhibit BACE-1, while the inhibitory ability of the hybrids of the other two series has not been evaluated [45]. Hybrids **67**–**94** and **149**–**153** exhibit antioxidant activity, while **806**–**826** are not antioxidants. The most successful choliesterase inhibitors are hybrids **67**–**94**; the lead compounds in this series are **88** with IC_50_ 0.035 nm against AChE and **74** with 0.038 nM against BuChE, while hybrids **149**–**153** and **806**–**826** demonstrated weaker inhibitory activity. Thus, the most successful design of THA–flavonoid hybrids is reported by Fernández-Bachiller et al. [45].

Comparison of structurally similar hybrids **128**–**148** and **192**–**210** with antioxidants Trolox and Cp-6 is also of interest [51,56]. Hybrids **128**–**148** with an amide linker showed moderate inhibitory activity, while hybrids **192**–**201** showed picomolar activity. The importance of the linker choice is confirmed again, which is confirmed by the order of activities in the second series: amines **207**–**210** > amides **192**–**195** > reverse amides **204**–**206** > O-benzylated amides **196**–**199**. That is, the amine linker between THA and the second residue without an acceptor amide bond plays one of the key roles in the activity of the hybrid. 

## 10. Conclusions

The design of THA hybrids is a promising alternative to anti-AD drugs used in clinical practice due to their high efficiency and multiple biological actions. In addition, synthesis of THA hybrids results in reducing hepatotoxicity. A wide possibility opens up with the conjugation of THA with a second biological active moiety, which provides an additional biological activity and several mechanisms of anti-AD therapy in one drug. Thus, in this review, many hybrids are able not only to inhibit cholinesterase, but also to affect amyloid aggregation and reduce the number of Aβ plaques in the brain. Additionally, many of the described hybrids showed a high selectivity for one of the cholinesterase, are able to donate NO and cause a vasorelaxant effect, inhibit BACE-1, cause an antidepressant and a neuroprotective effect, and chelate metal cations, thereby counteracting the formation of toxic amyloid aggregates. The impressive results of in vivo studies also confirm that THA derivatives are able to improve cognitive functions by not only cholinesterase inhibition, but also by reducing the number of amyloid plaques in AD brains, while not causing noticeable hepatotoxicity.

## Data Availability

Not applicable.

## Supplementary Materials

The following supporting information can be downloaded at: https://www.mdpi.com/article/10.3390/ijms24021717/s1.

## Abbreviations

AD—Alzheimer’s disease, Aβ—β-amyloid, ACh—acetylcholine, AChE—acetylcholinesterase, BuChE—butyrylcholinesterase, 6-Cl-THA—6-chlorotacrine, 7-MEOTA—7-metoxytacrine, HDAC—Histone deacetylase, THA—tacrine, i.c.v.—intracerebroventricular injection, i.p.—intraperitoneal injection, i.g.—intragastric administration, i.d.—intradermal injection, ORAC-FL—radical absorbance capacity assay using fluorescein, ALT—alanine aminotransferase, TPPU—N-[1-(1-Oxopropyl)-4-piperidinyl]-N′-[4-(trifluoromethoxy)phenyl]-urea, ASAT—aspartate aminotransferase, LDH—lactate dehydogenase, NSAIDs—nonsteroidal anti-inflammatory drugs, isosorbide mononitrate (ISMNI), MAOs—monoamine oxidases, FAD—flavin adenine dinucleotide, ROS—reactive oxygen species, TEM—transmission electron microscopic, LTP—long-term potentiation, CAS—catalytic active site, PAS—peripheral anionic site, GSK-3—glycogen synthase kinase-3, APP—amyloid precursor protein, LPS—bacterial endotoxin lipopolysaccharide, AFM—atomic force microscope, SPR—surface plasmon resonance, HSC—human hepatic stellate cells, PI—propidium iodide.

## References

[1] Takahashi R.H., Nagao T., Gouras G.K. (2017). Plaque formation and the intraneuronal accumulation of β-amyloid in Alzheimer’s disease. Pathol. Int..

[2] Das B., Yan R. (2017). Role of BACE1 in Alzheimer’s synaptic function. Transl. Neurodegener..

[3] Ali M.M., Ghouri R.G., Ans A.H., Akbar A., Toheed A. (2019). Recommendations for Anti-inflammatory Treatments in Alzheimer’s Disease: A Comprehensive Review of the Literature. Cureus.

[4] Huang W.-J., Zhang X., Chen W.-W. (2016). Role of oxidative stress in Alzheimer’s disease. Biomed. Rep..

[5] Arbel-Ornath M., Hudry E., Boivin J.R., Hashimoto T., Takeda S., Kuchibhotla K.V., Hou S., Lattarulo C.R., Belcher A.M., Shakerdge N. (2017). Soluble oligomeric amyloid-β induces calcium dyshomeostasis that precedes synapse loss in the living mouse brain. Mol. Neurodegener..

[6] Drews A., De S., Flagmeier P., Wirthensohn D.C., Chen W.-H., Whiten D.R., Rodrigues M., Vincke C., Muyldermans S., Paterson R.W. (2017). Inhibiting the Ca^2+^ Influx Induced by Human CSF. Cell Rep..

[7] Goodison W.V., Frisardi V., Kehoe P.G. (2012). Calcium Channel Blockers and Alzheimer’s Disease: Potential Relevance in Treatment Strategies of Metabolic Syndrome. J. Alzheimer’s Dis..

[8] Lauretti E., Dincer O., Praticò D. (2020). Glycogen synthase kinase-3 signaling in Alzheimer’s disease. Biochim. Biophys. Acta—Mol. Cell Res..

[9] Hooper C., Killick R., Lovestone S. (2008). The GSK3 hypothesis of Alzheimer’s disease. J. Neurochem..

[10] Gsell W., Jungkunz G., Riederer P. (2004). Functional Neurochemistry of Alzheimer’s Disease. Cur. Pharm. Dis..

[11] Werner F., Coveñas R. (2013). Classical Neurotransmitters and Neuropeptides Involved in Major Depression in a Multi-neurotransmitter System: A Focus on Antidepressant Drugs. Curr. Med. Chem..

[12] Ishibashi M., Yamazaki Y., Miledi R., Sumikawa K. (2014). Nicotinic and muscarinic agonists and acetylcholinesterase inhibitors stimulate a common pathway to enhance GluN2B-NMDAR responses. Proc. Natl. Acad. Sci. USA.

[13] Dwomoh L., Tejeda G.S., Tobin A.B. (2022). Targeting the M1 muscarinic acetylcholine receptor in Alzheimer’s disease. Neuronal Signal..

[14] Colovic M.B., Krstic D.Z., Lazarevic-Pasti T., Bondzic A.M., Vasic V.M. (2013). Acetylcholinesterase Inhibitors: Pharmacology and Toxicology. Curr. Neuropharmacol..

[15] Terry A.V., Callahan P., Hernandez C.M. (2015). Nicotinic ligands as multifunctional agents for the treatment of neuropsychiatric disorders. Biochem. Pharmacol..

[16] Hoskin J.L., Al-Hasan Y., Sabbagh M.N. (2019). Nicotinic Acetylcholine Receptor Agonists for the Treatment of Alzheimer’s Dementia: An Update. Nicotine Tob. Res..

[17] Greenlee W., Clader J., Asberom T., McCombie S., Ford J., Guzik H., Kozlowski J., Li S., Liu C., Lowe D. (2001). Muscarinic agonists and antagonists in the treatment of Alzheimer’s disease. Il Farm..

[18] Furuie H., Yamada K., Ichitani Y. (2013). MK-801-induced and scopolamine-induced hyperactivity in rats neonatally treated chronically with MK-801. Behav. Pharmacol..

[19] Francis P.T., Palmer A.M., Snape M., Wilcock G.K. (1999). The cholinergic hypothesis of Alzheimer’s disease: A review of progress. J. Neurol. Neurosurg. Psychiatry.

[20] Marucci G., Buccioni M., Ben D.D., Lambertucci C., Volpini R., Amenta F. (2021). Efficacy of acetylcholinesterase inhibitors in Alzheimer’s disease. Neuropharmacology.

[21] Tayeb H.O., Yang H.D., Price B.H., Tarazi F.I. (2012). Pharmacotherapies for Alzheimer’s disease: Beyond cholinesterase inhibitors. Pharmacol. Ther..

[22] (2012). LiverTox: Clinical and Research Information on Drug-Induced Liver Injury [Internet].

[23] Recanatini M., Cavalli A., Belluti F., Piazzi L., Rampa A., Bisi A., Gobbi S., Valenti P., Andrisano V., Bartolini M. (2000). SAR of 9-Amino-1,2,3,4-tetrahydroacridine-Based Acetylcholinesterase Inhibitors: Synthesis, Enzyme Inhibitory Activity, QSAR, and Structure-Based CoMFA of Tacrine Analogues. J. Med. Chem..

[24] Soukup O., Jun D., Zdarova-Karasova J., Patocka J., Musilek K., Korabecny J., Krusek J., Kaniakova M., Sepsova V., Mandikova J. (2013). A Resurrection of 7-MEOTA: A Comparison with Tacrine. Curr. Alzheimer Res..

[25] Korabecny J., Musilek K., Holas O., Binder J., Zemek F., Marek J., Pohanka M., Opletalova V., Dohnal V., Kuca K. (2010). Synthesis and in vitro evaluation of N-alkyl-7-methoxytacrine hydrochlorides as potential cholinesterase inhibitors in Alzheimer disease. Bioorg. Med. Chem. Lett..

[26] Quintanova C., Keri R.S., Marques S.M., Fernandes G.M., Cardoso S.M., Luísa Serralheiro M., Amélia Santos M. (2015). Design, synthesis and bioevaluation of tacrine hybrids with cinnamate and cinnamylidene acetate derivatives as potential anti-Alzheimer drugs. MedChemComm.

[27] Wu W.-Y., Dai Y.-C., Li N.-G., Dong Z.-X., Gu T., Shi Z.-H., Xue X., Tang Y.-P., Duan J.-A. (2017). Novel multitarget-directed tacrine derivatives as potential candidates for the treatment of Alzheimer’s disease. J. Enzyme Inhib. Med. Chem..

[28] Carlier P.R., Chow E.S., Han Y., Liu J., El Yazal J., Pang Y.-P. (1999). Heterodimeric Tacrine-Based Acetylcholinesterase Inhibitors: Investigating Ligand−Peripheral Site Interactions. J. Med. Chem..

[29] Harel M., Sonoda L.K., Silman I., Sussman J.L., Rosenberry T.L. (2008). Crystal Structure of Thioflavin T Bound to the Peripheral Site of Torpedo californica Acetylcholinesterase Reveals How Thioflavin T Acts as a Sensitive Fluorescent Reporter of Ligand Binding to the Acylation Site. J. Am. Chem. Soc..

[30] Chen Y., Sun J., Fang L., Liu M., Peng S., Liao H., Lehmann J., Zhang Y. (2012). Tacrine–Ferulic Acid–Nitric Oxide (NO) Donor Trihybrids as Potent, Multifunctional Acetyl- and Butyrylcholinesterase Inhibitors. J. Med. Chem..

[31] Carvajal F.J., Inestrosa N.C. (2011). Interactions of AChE with Aβ Aggregates in Alzheimer’s Brain: Therapeutic Relevance of IDN 5706. Front. Mol. Neurosci..

[32] Pourshojaei Y., Abiri A., Eskandari K., Haghighijoo Z., Edraki N., Asadipour A. (2019). Phenoxyethyl Piperidine/Morpholine Derivatives as PAS and CAS Inhibitors of Cholinesterases: Insights for Future Drug Design. Sci. Rep..

[33] Khoobi M., Ghanoni F., Nadri H., Moradi A., Hamedani M.P., Homayouni Moghadam F., Emami S., Vosooghi M., Zadmard R., Foroumadi A. (2015). New tetracyclic tacrine analogs containing pyrano[2,3-c]pyrazole: Efficient synthesis, biological assessment and docking simulation study. Eur. J. Med. Chem..

[34] Pourabdi L., Khoobi M., Nadri H., Moradi A., Moghadam F.H., Emami S., Mojtahedi M.M., Haririan I., Forootanfar H., Ameri A. (2016). Synthesis and structure-activity relationship study of tacrine-based pyrano[2,3-c]pyrazoles targeting AChE/BuChE and 15-LOX. Eur. J. Med. Chem..

[35] Roldán-Peña J.M., Alejandre-Ramos D., López Ó., Maya I., Lagunes I., Padrón J.M., Peña-Altamira L.E., Bartolini M., Monti B., Bolognesi M.L. (2017). New tacrine dimers with antioxidant linkers as dual drugs: Anti-Alzheimer’s and antiproliferative agents. Eur. J. Med. Chem..

[36] Sameem B., Saeedi M., Mahdavi M., Shafiee A. (2017). A review on tacrine-based scaffolds as multi-target drugs (MTDLs) for Alzheimer’s disease. Eur. J. Med. Chem..

[37] Girek M., Szymański P. (2019). Phyto-Tacrine Hybrids as Promising Drugs to Treat Alzheimer’s Disease. ChemistrySelect.

[38] Eckroat T.J., Manross D.L., Cowan S.C. (2020). Merged Tacrine-Based, Multitarget-Directed Acetylcholinesterase Inhibitors 2015–Present: Synthesis and Biological Activity. Int. J. Mol. Sci..

[39] Rodríguez-Franco M.I., Fernández-Bachiller M.I., Pérez C., Hernández-Ledesma B., Bartolomé B. (2006). Novel Tacrine−Melatonin Hybrids as Dual-Acting Drugs for Alzheimer Disease, with Improved Acetylcholinesterase Inhibitory and Antioxidant Properties. J. Med. Chem..

[40] Fernández-Bachiller M.I., Pérez C., Campillo N.E., Páez J.A., González-Muñoz G.C., Usán P., García-Palomero E., López M.G., Villarroya M., García A.G. (2009). Tacrine-Melatonin Hybrids as Multifunctional Agents for Alzheimer’s Disease, with Cholinergic, Antioxidant, and Neuroprotective Properties. ChemMedChem.

[41] Benchekroun M., Romero A., Egea J., León R., Michalska P., Buendía I., Jimeno M.L., Jun D., Janockova J., Sepsova V. (2016). The Antioxidant Additive Approach for Alzheimer’s Disease Therapy: New Ferulic (Lipoic) Acid Plus Melatonin Modified Tacrines as Cholinesterases Inhibitors, Direct Antioxidants, and Nuclear Factor (Erythroid-Derived 2)-Like 2 Activators. J. Med. Chem..

[42] Fernández-Bachiller M.I., Pérez C., González-Muñoz G.C., Conde S., López M.G., Villarroya M., García A.G., Rodríguez-Franco M.I. (2010). Novel Tacrine−8-Hydroxyquinoline Hybrids as Multifunctional Agents for the Treatment of Alzheimer’s Disease, with Neuroprotective, Cholinergic, Antioxidant, and Copper-Complexing Properties. J. Med. Chem..

[43] Luo W., Li Y.-P., He Y., Huang S.-L., Tan J.-H., Ou T.-M., Li D., Gu L.-Q., Huang Z.-S. (2011). Design, synthesis and evaluation of novel tacrine-multialkoxybenzene hybrids as dual inhibitors for cholinesterases and amyloid beta aggregation. Bioorg. Med. Chem..

[44] Luo W., Li Y.-P., He Y., Huang S.-L., Li D., Gu L.-Q., Huang Z.-S. (2011). Synthesis and evaluation of heterobivalent tacrine derivatives as potential multi-functional anti-Alzheimer agents. Eur. J. Med. Chem..

[45] Fernández-Bachiller M.I., Pérez C., Monjas L., Rademann J., Rodríguez-Franco M.I. (2012). New Tacrine–4-Oxo-4 H -chromene Hybrids as Multifunctional Agents for the Treatment of Alzheimer’s Disease, with Cholinergic, Antioxidant, and β-Amyloid-Reducing Properties. J. Med. Chem..

[46] Chao X., He X., Yang Y., Zhou X., Jin M., Liu S., Cheng Z., Liu P., Wang Y., Yu J. (2012). Design, synthesis and pharmacological evaluation of novel tacrine–caffeic acid hybrids as multi-targeted compounds against Alzheimer’s disease. Bioorg. Med. Chem. Lett..

[47] Chen X., Zenger K., Lupp A., Kling B., Heilmann J., Fleck C., Kraus B., Decker M. (2012). Tacrine-Silibinin Codrug Shows Neuro- and Hepatoprotective Effects in Vitro and Pro-Cognitive and Hepatoprotective Effects in Vivo. J. Med. Chem..

[48] Zenger K., Chen X., Decker M., Kraus B. (2013). In-vitro stability and metabolism of a tacrine–silibinin codrug. J. Pharm. Pharmacol..

[49] Mao F., Chen J., Zhou Q., Luo Z., Huang L., Li X. (2013). Novel tacrine–ebselen hybrids with improved cholinesterase inhibitory, hydrogen peroxide and peroxynitrite scavenging activity. Bioorg. Med. Chem. Lett..

[50] Lan J.-S., Xie S.-S., Li S.-Y., Pan L.-F., Wang X.-B., Kong L.-Y. (2014). Design, synthesis and evaluation of novel tacrine-(β-carboline) hybrids as multifunctional agents for the treatment of Alzheimer’s disease. Bioorg. Med. Chem..

[51] Nepovimova E., Korabecny J., Dolezal R., Babkova K., Ondrejicek A., Jun D., Sepsova V., Horova A., Hrabinova M., Soukup O. (2015). Tacrine–Trolox Hybrids: A Novel Class of Centrally Active, Nonhepatotoxic Multi-Target-Directed Ligands Exerting Anticholinesterase and Antioxidant Activities with Low In Vivo Toxicity. J. Med. Chem..

[52] Luo W., Wang T., Hong C., Yang Y., Chen Y., Cen J., Xie S., Wang C. (2016). Design, synthesis and evaluation of 4-dimethylamine flavonoid derivatives as potential multifunctional anti-Alzheimer agents. Eur. J. Med. Chem..

[53] Chand K., Alsoghier H.M., Chaves S., Santos M.A. (2016). Tacrine-(hydroxybenzoyl-pyridone) hybrids as potential multifunctional anti-Alzheimer’s agents: AChE inhibition, antioxidant activity and metal chelating capacity. J. Inorg. Biochem..

[54] Li G., Hong G., Li X., Zhang Y., Xu Z., Mao L., Feng X., Liu T. (2018). Synthesis and activity towards Alzheimer’s disease in vitro: Tacrine, phenolic acid and ligustrazine hybrids. Eur. J. Med. Chem..

[55] Li K., Jiang Y., Li G., Liu T., Yang Z. (2020). Novel Multitarget Directed Tacrine Hybrids as Anti-Alzheimer’s Compounds Improved Synaptic Plasticity and Cognitive Impairment in APP/PS1 Transgenic Mice. ACS Chem. Neurosci..

[56] Pérez-Areales F., Garrido M., Aso E., Bartolini M., De Simone A., Espargaró A., Ginex T., Sabate R., Pérez B., Andrisano V. (2020). Centrally Active Multitarget Anti-Alzheimer Agents Derived from the Antioxidant Lead CR-6. J. Med. Chem..

[57] Rani A., Singh A., Kaur J., Singh G., Bhatti R., Gumede N., Kisten P., Singh P., Sumanjit, Kumar V. (2021). 1H-1,2,3-triazole grafted tacrine-chalcone conjugates as potential cholinesterase inhibitors with the evaluation of their behavioral tests and oxidative stress in mice brain cells. Bioorg. Chem..

[58] Viayna E., Coquelle N., Cieslikiewicz-Bouet M., Cisternas P., Oliva C.A., Sánchez-López E., Ettcheto M., Bartolini M., De Simone A., Ricchini M. (2021). Discovery of a Potent Dual Inhibitor of Acetylcholinesterase and Butyrylcholinesterase with Antioxidant Activity that Alleviates Alzheimer-like Pathology in Old APP/PS1 Mice. J. Med. Chem..

[59] Fang L., Kraus B., Lehmann J., Heilmann J., Zhang Y., Decker M. (2008). Design and synthesis of tacrine–ferulic acid hybrids as multi-potent anti-Alzheimer drug candidates. Bioorg. Med. Chem. Lett..

[60] Fleck C., Appenroth D., Fang L., Schott Y., Lehmann J., Decker M. (2011). Investigation into the in vivo effects of five novel tacrine/ferulic acid and β-carboline derivatives on scopolamine-induced cognitive impairment in rats using radial maze paradigm. Arzneimittelforschung.

[61] Pi R., Mao X., Chao X., Cheng Z., Liu M., Duan X., Ye M., Chen X., Mei Z., Liu P. (2012). Tacrine-6-Ferulic Acid, a Novel Multifunctional Dimer, Inhibits Amyloid-β-Mediated Alzheimer’s Disease-Associated Pathogenesis In Vitro and In Vivo. PLoS ONE.

[62] Fu Y., Mu Y., Lei H., Wang P., Li X., Leng Q., Han L., Qu X., Wang Z., Huang X. (2016). Design, Synthesis and Evaluation of Novel Tacrine-Ferulic Acid Hybrids as Multifunctional Drug Candidates against Alzheimer’s Disease. Molecules.

[63] Zhu J., Yang H., Chen Y., Lin H., Li Q., Mo J., Bian Y., Pei Y., Sun H. (2018). Synthesis, pharmacology and molecular docking on multifunctional tacrine-ferulic acid hybrids as cholinesterase inhibitors against Alzheimer’s disease. J. Enzyme Inhib. Med. Chem..

[64] Fang L., Appenroth D., Decker M., Kiehntopf M., Roegler C., Deufel T., Fleck C., Peng S., Zhang Y., Lehmann J. (2008). Synthesis and Biological Evaluation of NO-Donor-Tacrine Hybrids as Hepatoprotective Anti-Alzheimer Drug Candidates. J. Med. Chem..

[65] Fang L., Appenroth D., Decker M., Kiehntopf M., Lupp A., Peng S., Fleck C., Zhang Y., Lehmann J. (2008). NO-Donating Tacrine Hybrid Compounds Improve Scopolamine-Induced Cognition Impairment and Show Less Hepatotoxicity. J. Med. Chem..

[66] Hui A., Chen Y., Zhu S., Gan C., Pan J., Zhou A. (2014). Design and synthesis of tacrine-phenothiazine hybrids as multitarget drugs for Alzheimer’s disease. Med. Chem. Res..

[67] Gorecki L., Uliassi E., Bartolini M., Janockova J., Hrabinova M., Hepnarova V., Prchal L., Muckova L., Pejchal J., Karasova J.Z. (2021). Phenothiazine-Tacrine Heterodimers: Pursuing Multitarget Directed Approach in Alzheimer’s Disease. ACS Chem. Neurosci..

[68] Huang L., Su T., Shan W., Luo Z., Sun Y., He F., Li X. (2012). Inhibition of cholinesterase activity and amyloid aggregation by berberine-phenyl-benzoheterocyclic and tacrine-phenyl-benzoheterocyclic hybrids. Bioorg. Med. Chem..

[69] Keri R.S., Quintanova C., Marques S.M., Esteves A.R., Cardoso S.M., Santos M.A. (2013). Design, synthesis and neuroprotective evaluation of novel tacrine–benzothiazole hybrids as multi-targeted compounds against Alzheimer’s disease. Bioorg. Med. Chem..

[70] Zha X., Lamba D., Zhang L., Lou Y., Xu C., Kang D., Chen L., Xu Y., Zhang L., De Simone A. (2016). Novel Tacrine–Benzofuran Hybrids as Potent Multitarget-Directed Ligands for the Treatment of Alzheimer’s Disease: Design, Synthesis, Biological Evaluation, and X-ray Crystallography. J. Med. Chem..

[71] Rajeshwari R., Chand K., Candeias E., Cardoso S., Chaves S., Santos M. (2019). New Multitarget Hybrids Bearing Tacrine and Phenylbenzothiazole Motifs as Potential Drug Candidates for Alzheimer’s Disease. Molecules.

[72] Fancellu G., Chand K., Tomás D., Orlandini E., Piemontese L., Silva D.F., Cardoso S.M., Chaves S., Santos M.A. (2020). Novel tacrine–benzofuran hybrids as potential multi-target drug candidates for the treatment of Alzheimer’s Disease. J. Enzyme Inhib. Med. Chem..

[73] Nepovimova E., Svobodova L., Dolezal R., Hepnarova V., Junova L., Jun D., Korabecny J., Kucera T., Gazova Z., Motykova K. (2021). Tacrine-Benzothiazoles: Novel class of potential multitarget anti-Alzheimeŕs drugs dealing with cholinergic, amyloid and mitochondrial systems. Bioorg. Chem..

[74] Chen Y., Sun J., Peng S., Liao H., Zhang Y., Lehmann J. (2013). Tacrine-Flurbiprofen Hybrids as Multifunctional Drug Candidates for the Treatment of Alzheimer’s Disease. Arch. Pharm..

[75] Chen Y., Sun J., Huang Z., Liao H., Peng S., Lehmann J., Zhang Y. (2013). NO-donating tacrine derivatives as potential butyrylcholinesterase inhibitors with vasorelaxation activity. Bioorg. Med. Chem. Lett..

[76] Chen Y., Sun J., Huang Z., Liao H., Peng S., Lehmann J., Zhang Y. (2013). Design, synthesis and evaluation of tacrine–flurbiprofen–nitrate trihybrids as novel anti-Alzheimer’s disease agents. Bioorg. Med. Chem..

[77] Zawada K., Czarnecka K., Girek M., Kręcisz P., Trejtnar F., Mandíková J., Jończyk J., Bajda M., Staśkiewicz M., Wójtowicz P. (2021). New hybrids of tacrine and indomethacin as multifunctional acetylcholinesterase inhibitors. Chem. Pap..

[78] Liu Z., Zhang B., Xia S., Fang L., Gou S. (2021). ROS-responsive and multifunctional anti-Alzheimer prodrugs: Tacrine-ibuprofen hybrids via a phenyl boronate linker. Eur. J. Med. Chem..

[79] Chen H., Wu X., Gu X., Zhou Y., Ye L., Zhang K., Pan H., Wang J., Wei H., Zhu B. (2018). Tacrine(10)-Hupyridone Prevents Post-operative Cognitive Dysfunction via the Activation of BDNF Pathway and the Inhibition of AChE in Aged Mice. Front. Cell. Neurosci..

[80] Chen H., Xiang S., Huang L., Lin J., Hu S., Mak S.-H., Wang C., Wang Q., Cui W., Han Y. (2018). Tacrine(10)-hupyridone, a dual-binding acetylcholinesterase inhibitor, potently attenuates scopolamine-induced impairments of cognition in mice. Metab. Brain Dis..

[81] Xuan Z., Gu X., Yan S., Xie Y., Zhou Y., Zhang H., Jin H., Hu S., Mak M.S.H., Zhou D. (2021). Dimeric Tacrine(10)-hupyridone as a Multitarget-Directed Ligand To Treat Alzheimer’s Disease. ACS Chem. Neurosci..

[82] Shao D., Zou C., Luo C., Tang X., Li Y. (2004). Synthesis and evaluation of tacrine–E2020 hybrids as acetylcholinesterase inhibitors for the treatment of Alzheimer’s disease. Bioorg. Med. Chem. Lett..

[83] Camps P., Formosa X., Galdeano C., Gómez T., Muñoz-Torrero D., Scarpellini M., Viayna E., Badia A., Clos M.V., Camins A. (2008). Novel Donepezil-Based Inhibitors of Acetyl- and Butyrylcholinesterase and Acetylcholinesterase-Induced β-Amyloid Aggregation. J. Med. Chem..

[84] Codony S., Pont C., Griñán-Ferré C., Di Pede-Mattatelli A., Calvó-Tusell C., Feixas F., Osuna S., Jarné-Ferrer J., Naldi M., Bartolini M. (2022). Discovery and In Vivo Proof of Concept of a Highly Potent Dual Inhibitor of Soluble Epoxide Hydrolase and Acetylcholinesterase for the Treatment of Alzheimer’s Disease. J. Med. Chem..

[85] Galdeano C., Viayna E., Sola I., Formosa X., Camps P., Badia A., Clos M.V., Relat J., Ratia M., Bartolini M. (2012). Huprine–Tacrine Heterodimers as Anti-Amyloidogenic Compounds of Potential Interest against Alzheimer’s and Prion Diseases. J. Med. Chem..

[86] Cen J., Guo H., Hong C., Lv J., Yang Y., Wang T., Fang D., Luo W., Wang C. (2018). Development of tacrine-bifendate conjugates with improved cholinesterase inhibitory and pro-cognitive efficacy and reduced hepatotoxicity. Eur. J. Med. Chem..

[87] Xu A., He F., Zhang X., Li X., Ran Y., Wei C., James Chou C., Zhang R., Wu J. (2020). Tacrine-hydroxamate derivatives as multitarget-directed ligands for the treatment of Alzheimer’s disease: Design, synthesis, and biological evaluation. Bioorg. Chem..

[88] Wang Y., Guan X.-L., Wu P.-F., Wang C.-M., Cao H., Li L., Guo X.-J., Wang F., Xie N., Jiang F.-C. (2012). Multifunctional Mercapto-tacrine Derivatives for Treatment of Age-Related Neurodegenerative Diseases. J. Med. Chem..

[89] Keri R.S., Quintanova C., Chaves S., Silva D.F., Cardoso S.M., Santos M.A. (2016). New Tacrine Hybrids with Natural-Based Cysteine Derivatives as Multitargeted Drugs for Potential Treatment of Alzheimer’s Disease. Chem. Biol. Drug Des..

[90] Cheng X., Gu J., Pang Y., Liu J., Xu T., Li X., Hua Y., Newell K.A., Huang X.-F., Yu Y. (2019). Tacrine–Hydrogen Sulfide Donor Hybrid Ameliorates Cognitive Impairment in the Aluminum Chloride Mouse Model of Alzheimer’s Disease. ACS Chem. Neurosci..

[91] Camps P., Formosa X., Galdeano C., Muñoz-Torrero D., Ramírez L., Gómez E., Isambert N., Lavilla R., Badia A., Clos M.V. (2009). Pyrano[3,2-c]quinoline−6-Chlorotacrine Hybrids as a Novel Family of Acetylcholinesterase- and β-Amyloid-Directed Anti-Alzheimer Compounds. J. Med. Chem..

[92] Di Pietro O., Pérez-Areales F., Juárez-Jiménez J., Espargaró A., Clos M.V., Pérez B., Lavilla R., Sabaté R., Luque F.J., Muñoz-Torrero D. (2014). Tetrahydrobenzo[h][1,6]naphthyridine-6-chlorotacrine hybrids as a new family of anti-Alzheimer agents targeting β-amyloid, tau, and cholinesterase pathologies. Eur. J. Med. Chem..

[93] da Costa J.S., Lopes J.P.B., Russowsky D., Petzhold C.L., de Borges A.C., Ceschi M.A., Konrath E., Batassini C., Lunardi P.S., Gonçalves C.A.S. (2013). Synthesis of tacrine-lophine hybrids via one-pot four component reaction and biological evaluation as acetyl- and butyrylcholinesterase inhibitors. Eur. J. Med. Chem..

[94] Marco-Contelles J., León R., de los Ríos C., Guglietta A., Terencio J., López M., García A., Villarroya M. (2006). Novel Multipotent Tacrine−Dihydropyridine Hybrids with Improved Acetylcholinesterase Inhibitory and Neuroprotective Activities as Potential Drugs for the Treatment of Alzheimer’s Disease. J. Med. Chem..

[95] Marco-Contelles J., León R., de los Ríos C., Samadi A., Bartolini M., Andrisano V., Huertas O., Barril X., Luque F.J., Rodríguez-Franco M. (2009). Tacripyrines, the First Tacrine−Dihydropyridine Hybrids, as Multitarget-Directed Ligands for the Treatment of Alzheimer’s Disease. J. Med. Chem..

[96] Bartolini M., Pistolozzi M., Andrisano V., Egea J., López M.G., Iriepa I., Moraleda I., Gálvez E., Marco-Contelles J., Samadi A. (2011). Chemical and Pharmacological Studies on Enantiomerically Pure p-Methoxytacripyrines, Promising Multi-Target-Directed Ligands for the Treatment of Alzheimer’s Disease. ChemMedChem.

[97] Wang X.-L., Xiong Y., Yang Y., Tuo Q., Wang X., Chen R., Tian Q., Zhang Z., Yan X., Yang Z. (2015). A novel tacrine-dihydropyridine hybrid (-)SCR1693 induces tau dephosphorylation and inhibits Aβ generation in cells. Eur. J. Pharmacol..

[98] Chioua M., Buzzi E., Moraleda I., Iriepa I., Maj M., Wnorowski A., Giovannini C., Tramarin A., Portali F., Ismaili L. (2018). Tacripyrimidines, the first tacrine-dihydropyrimidine hybrids, as multi-target-directed ligands for Alzheimer’s disease. Eur. J. Med. Chem..

[99] Sola I., Aso E., Frattini D., López-González I., Espargaró A., Sabaté R., Di Pietro O., Luque F.J., Clos M.V., Ferrer I. (2015). Novel Levetiracetam Derivatives That Are Effective against the Alzheimer-like Phenotype in Mice: Synthesis, in Vitro, ex Vivo, and in Vivo Efficacy Studies. J. Med. Chem..

[100] Więckowska A., Kołaczkowski M., Bucki A., Godyń J., Marcinkowska M., Więckowski K., Zaręba P., Siwek A., Kazek G., Głuch-Lutwin M. (2016). Novel multi-target-directed ligands for Alzheimer’s disease: Combining cholinesterase inhibitors and 5-HT 6 receptor antagonists. Design, synthesis and biological evaluation. Eur. J. Med. Chem..

[101] Więckowska A., Wichur T., Godyń J., Bucki A., Marcinkowska M., Siwek A., Więckowski K., Zaręba P., Knez D., Głuch-Lutwin M. (2018). Novel Multitarget-Directed Ligands Aiming at Symptoms and Causes of Alzheimer’s Disease. ACS Chem. Neurosci..

[102] Li X., Wang H., Xu Y., Liu W., Gong Q., Wang W., Qiu X., Zhu J., Mao F., Zhang H. (2017). Novel Vilazodone–Tacrine Hybrids as Potential Multitarget-Directed Ligands for the Treatment of Alzheimer’s Disease Accompanied with Depression: Design, Synthesis, and Biological Evaluation. ACS Chem. Neurosci..

[103] Elsinghorst P.W., Cieslik J.S., Mohr K., Tränkle C., Gütschow M. (2007). First Gallamine−Tacrine Hybrid: Design and Characterization at Cholinesterases and the M 2 Muscarinic Receptor. J. Med. Chem..

[104] Fang L., Jumpertz S., Zhang Y., Appenroth D., Fleck C., Mohr K., Tränkle C., Decker M. (2010). Hybrid Molecules from Xanomeline and Tacrine: Enhanced Tacrine Actions on Cholinesterases and Muscarinic M 1 Receptors. J. Med. Chem..

[105] Hepnarova V., Korabecny J., Matouskova L., Jost P., Muckova L., Hrabinova M., Vykoukalova N., Kerhartova M., Kucera T., Dolezal R. (2018). The concept of hybrid molecules of tacrine and benzyl quinolone carboxylic acid (BQCA) as multifunctional agents for Alzheimer’s disease. Eur. J. Med. Chem..

[106] Maspero M., Volpato D., Cirillo D., Chen N.Y., Messerer R., Sotriffer C., De Amici M., Holzgrabe U., Dallanoce C. (2020). Tacrine-xanomeline and tacrine-iperoxo hybrid ligands: Synthesis and biological evaluation at acetylcholinesterase and M1 muscarinic acetylcholine receptors. Bioorg. Chem..

[107] Lange J.H.M., Coolen H.K.A.C., van der Neut M.A.W., Borst A.J.M., Stork B., Verveer P.C., Kruse C.G. (2010). Design, Synthesis, Biological Properties, and Molecular Modeling Investigations of Novel Tacrine Derivatives with a Combination of Acetylcholinesterase Inhibition and Cannabinoid CB 1 Receptor Antagonism. J. Med. Chem..

[108] Spilovska K., Korabecny J., Kral J., Horova A., Musilek K., Soukup O., Drtinova L., Gazova Z., Siposova K., Kuca K. (2013). 7-Methoxytacrine-Adamantylamine Heterodimers as Cholinesterase Inhibitors in Alzheimer’s Disease Treatment—Synthesis, Biological Evaluation and Molecular Modeling Studies. Molecules.

[109] Pérez-Areales F.J., Turcu A.L., Barniol-Xicota M., Pont C., Pivetta D., Espargaró A., Bartolini M., De Simone A., Andrisano V., Pérez B. (2019). A novel class of multitarget anti-Alzheimer benzohomoadamantane chlorotacrine hybrids modulating cholinesterases and glutamate NMDA receptors. Eur. J. Med. Chem..

[110] Ceschi M.A., da Costa J.S., Lopes J.P.B., Câmara V.S., Campo L.F., de Borges A.C., Gonçalves C., de Souza D., Konrath E.L., Karl A.L.M. (2016). Novel series of tacrine-tianeptine hybrids: Synthesis, cholinesterase inhibitory activity, S100B secretion and a molecular modeling approach. Eur. J. Med. Chem..

[111] Lu C., Zhou Q., Yan J., Du Z., Huang L., Li X. (2013). A novel series of tacrine–selegiline hybrids with cholinesterase and monoamine oxidase inhibition activities for the treatment of Alzheimer’s disease. Eur. J. Med. Chem..

[112] Xie S.-S., Wang X., Jiang N., Yu W., Wang K.D.G., Lan J.-S., Li Z.-R., Kong L.-Y. (2015). Multi-target tacrine-coumarin hybrids: Cholinesterase and monoamine oxidase B inhibition properties against Alzheimer’s disease. Eur. J. Med. Chem..

[113] Xie S.-S., Wang X.-B., Li J.-Y., Yang L., Kong L.-Y. (2013). Design, synthesis and evaluation of novel tacrine–coumarin hybrids as multifunctional cholinesterase inhibitors against Alzheimer’s disease. Eur. J. Med. Chem..

[114] Hamulakova S., Janovec L., Hrabinova M., Spilovska K., Korabecny J., Kristian P., Kuca K., Imrich J. (2014). Synthesis and Biological Evaluation of Novel Tacrine Derivatives and Tacrine−Coumarin Hybrids as Cholinesterase Inhibitors. J. Med. Chem..

[115] Li S.-Y., Wang X.-B., Xie S.-S., Jiang N., Wang K.D.G., Yao H.-Q., Sun H.-B., Kong L.-Y. (2013). Multifunctional tacrine–flavonoid hybrids with cholinergic, β-amyloid-reducing, and metal chelating properties for the treatment of Alzheimer’s disease. Eur. J. Med. Chem..

[116] Viayna E., Sola I., Bartolini M., De Simone A., Tapia-Rojas C., Serrano F.G., Sabaté R., Juárez-Jiménez J., Pérez B., Luque F.J. (2014). Synthesis and Multitarget Biological Profiling of a Novel Family of Rhein Derivatives As Disease-Modifying Anti-Alzheimer Agents. J. Med. Chem..

[117] Thiratmatrakul S., Yenjai C., Waiwut P., Vajragupta O., Reubroycharoen P., Tohda M., Boonyarat C. (2014). Synthesis, biological evaluation and molecular modeling study of novel tacrine–carbazole hybrids as potential multifunctional agents for the treatment of Alzheimer’s disease. Eur. J. Med. Chem..

[118] Spilovska K., Korabecny J., Sepsova V., Jun D., Hrabinova M., Jost P., Muckova L., Soukup O., Janockova J., Kucera T. (2017). Novel Tacrine-Scutellarin Hybrids as Multipotent Anti-Alzheimer’s Agents: Design, Synthesis and Biological Evaluation. Molecules.

[119] Jeřábek J., Uliassi E., Guidotti L., Korábečný J., Soukup O., Sepsova V., Hrabinova M., Kuča K., Bartolini M., Peña-Altamira L.E. (2017). Tacrine-resveratrol fused hybrids as multi-target-directed ligands against Alzheimer’s disease. Eur. J. Med. Chem..

[120] Lopes J.P.B., Silva L., da Costa Franarin G., Ceschi M.A., Lüdtke D.S., Dantas R.F., de Salles C., Silva F.P., Senger M.R., Guedes I.A. (2018). Design, synthesis, cholinesterase inhibition and molecular modelling study of novel tacrine hybrids with carbohydrate derivatives. Bioorg. Med. Chem..

[121] Chalupova K., Korabecny J., Bartolini M., Monti B., Lamba D., Caliandro R., Pesaresi A., Brazzolotto X., Gastellier A.-J., Nachon F. (2019). Novel tacrine-tryptophan hybrids: Multi-target directed ligands as potential treatment for Alzheimer’s disease. Eur. J. Med. Chem..

[122] Cheng Z.-Q., Zhu K.-K., Zhang J., Song J.-L., Muehlmann L.A., Jiang C.-S., Liu C.-L., Zhang H. (2019). Molecular-docking-guided design and synthesis of new IAA-tacrine hybrids as multifunctional AChE/BuChE inhibitors. Bioorg. Chem..

[123] Rossi M., Freschi M., de Camargo Nascente L., Salerno A., de Melo Viana Teixeira S., Nachon F., Chantegreil F., Soukup O., Prchal L., Malaguti M. (2021). Sustainable Drug Discovery of Multi-Target-Directed Ligands for Alzheimer’s Disease. J. Med. Chem..

[124] Elsinghorst P.W., González Tanarro C.M., Gütschow M. (2006). Novel Heterobivalent Tacrine Derivatives as Cholinesterase Inhibitors with Notable Selectivity Toward Butyrylcholinesterase. J. Med. Chem..

[125] Chen X., Wehle S., Kuzmanovic N., Merget B., Holzgrabe U., König B., Sotriffer C.A., Decker M. (2014). Acetylcholinesterase Inhibitors with Photoswitchable Inhibition of β-Amyloid Aggregation. ACS Chem. Neurosci..

[126] Nepovimova E., Uliassi E., Korabecny J., Peña-Altamira L.E., Samez S., Pesaresi A., Garcia G.E., Bartolini M., Andrisano V., Bergamini C. (2014). Multitarget Drug Design Strategy: Quinone–Tacrine Hybrids Designed To Block Amyloid-β Aggregation and To Exert Anticholinesterase and Antioxidant Effects. J. Med. Chem..

[127] Mao F., Li J., Wei H., Huang L., Li X. (2015). Tacrine-propargylamine derivatives with improved acetylcholinesterase inhibitory activity and lower hepatotoxicity as a potential lead compound for the treatment of Alzheimers disease. J. Enzyme Inhib. Med. Chem..

[128] Korabecny J., Andrs M., Nepovimova E., Dolezal R., Babkova K., Horova A., Malinak D., Mezeiova E., Gorecki L., Sepsova V. (2015). 7-Methoxytacrine-p-Anisidine Hybrids as Novel Dual Binding Site Acetylcholinesterase Inhibitors for Alzheimer’s Disease Treatment. Molecules.

[129] Najafi Z., Mahdavi M., Saeedi M., Karimpour-Razkenari E., Asatouri R., Vafadarnejad F., Moghadam F.H., Khanavi M., Sharifzadeh M., Akbarzadeh T. (2017). Novel tacrine-1,2,3-triazole hybrids: In vitro, in vivo biological evaluation and docking study of cholinesterase inhibitors. Eur. J. Med. Chem..

[130] Riazimontazer E., Sadeghpour H., Nadri H., Sakhteman A., Tüylü Küçükkılınç T., Miri R., Edraki N. (2019). Design, synthesis and biological activity of novel tacrine-isatin Schiff base hybrid derivatives. Bioorg. Chem..

[131] Yao H., Uras G., Zhang P., Xu S., Yin Y., Liu J., Qin S., Li X., Allen S., Bai R. (2021). Discovery of Novel Tacrine–Pyrimidone Hybrids as Potent Dual AChE/GSK-3 Inhibitors for the Treatment of Alzheimer’s Disease. J. Med. Chem..

[132] Ozten O., Zengin Kurt B., Sonmez F., Dogan B., Durdagi S. (2021). Synthesis, molecular docking and molecular dynamics studies of novel tacrine-carbamate derivatives as potent cholinesterase inhibitors. Bioorg. Chem..

[133] Przybyłowska M., Dzierzbicka K., Kowalski S., Demkowicz S., Daśko M., Inkielewicz-Stepniak I. (2022). Design, synthesis and biological evaluation of novel N -phosphorylated and O -phosphorylated tacrine derivatives as potential drugs against Alzheimer’s disease. J. Enzyme Inhib. Med. Chem..

[134] Sun T.C., Liu X.C., Yang S.H., Song L.L., Zhou S.J., Deng S.L., Tian L., Cheng L.Y. (2020). Melatonin Inhibits Oxidative Stress and Apoptosis in Cryopreserved Ovarian Tissues via Nrf2/HO-1 Signaling Pathway. Front. Mol. Biosci..

[135] Rosini M., Andrisano V., Bartolini M., Bolognesi M.L., Hrelia P., Minarini A., Tarozzi A., Melchiorre C. (2005). Rational Approach To Discover Multipotent Anti-Alzheimer Drugs. J. Med. Chem..

[136] Yang N., Jia X., Wang D., Wei C., He Y., Chen L., Zhao Y. (2019). Silibinin as a natural antioxidant for modifying polysulfone membranes to suppress hemodialysis-induced oxidative stress. J. Memb. Sci..

[137] Schewe T. (1995). Molecular actions of Ebselen—An antiinflammatory antioxidant. Gen. Pharmacol. Vasc. Syst..

[138] Porciúncula L.O., Rocha J.B.T., Boeck C.R., Vendite D., Souza D.O. (2001). Ebselen prevents excitotoxicity provoked by glutamate in rat cerebellar granule neurons. Neurosci. Lett..

[139] Wlodek S.T., Antosiewicz J., McCammon J.A., Straatsma T.P., Gilson M.K., Briggs J.M., Humblet C., Sussman J.L. (1996). Binding of tacrine and 6-chlorotacrine by acetylcholinesterase. Biopolymers.

[140] Tran T.-D., Nguyen T.-C.-V., Nguyen N.-S., Nguyen D.-M., Nguyen T.-T.-H., Le M.-T., Thai K.-M. (2016). Synthesis of Novel Chalcones as Acetylcholinesterase Inhibitors. Appl. Sci..

[141] Mezeiova E., Soukup O., Korabecny J. (2020). Huprines—An insight into the synthesis and biological properties. Russ. Chem. Rev..

[142] Yang G., Wang Y., Tian J., Liu J.-P. (2013). Huperzine A for Alzheimer’s Disease: A Systematic Review and Meta-Analysis of Randomized Clinical Trials. PLoS ONE.

[143] Zhang H.Y., Tang X.C. (2006). Neuroprotective effects of huperzine A: New therapeutic targets for neurodegenerative disease. Trends Pharmacol. Sci..

[144] Muñoz-Torrero D., Camps P. (2008). Huprines for Alzheimer’s disease drug development. Expert Opin. Drug Discov..

[145] Kim J.K., Park S.U. (2019). A recent overview on the biological and pharmacological activities of ferulic acid. Excli J..

[146] Meng G., Meng X., Ma X., Zhang G., Hu X., Jin A., Zhao Y., Liu X. (2018). Application of Ferulic Acid for Alzheimer’s Disease: Combination of Text Mining and Experimental Validation. Front. Neuroinform..

[147] Simola N., Morelli M., Carta A.R. (2007). The 6-Hydroxydopamine model of parkinson’s disease. Neurotox. Res..

[148] Tsikas D. (2007). Analysis of nitrite and nitrate in biological fluids by assays based on the Griess reaction: Appraisal of the Griess reaction in the l-arginine/nitric oxide area of research. J. Chromatogr. B.

[149] Kerwin J.F., Heller M. (1994). The arginine-nitric oxide pathway: A target for new drugs. Med. Res. Rev..

[150] Esplugues J.V. (2002). NO as a signalling molecule in the nervous system. Br. J. Pharmacol..

[151] Balez R., Ooi L. (2016). Getting to NO Alzheimer’s Disease: Neuroprotection versus Neurotoxicity Mediated by Nitric Oxide. Oxid. Med. Cell. Longev..

[152] Webb D.J., Megson I.L. (2002). Nitric oxide donor drugs: Current status and future trends. Expert Opin. Investig. Drugs.

[153] Thatcher G., Bennett B., Reynolds J. (2005). Nitric Oxide Mimetic Molecules as Therapeutic Agents in Alzheimers Disease. Curr. Alzheimer Res..

[154] Chegaev K., Federico A., Marini E., Rolando B., Fruttero R., Morbin M., Rossi G., Fugnanesi V., Bastone A., Salmona M. (2015). NO-donor thiacarbocyanines as multifunctional agents for Alzheimer’s disease. Bioorg. Med. Chem..

[155] Jafari S., Fernandez-Enright F., Huang X.-F. (2012). Structural contributions of antipsychotic drugs to their therapeutic profiles and metabolic side effects. J. Neurochem..

[156] Krasnovskaya O., Spector D., Zlobin A., Pavlov K., Gorelkin P., Erofeev A., Beloglazkina E., Majouga A. (2020). Metals in Imaging of Alzheimer’s Disease. Int. J. Mol. Sci..

[157] Ono M., Kawashima H., Nonaka A., Kawai T., Haratake M., Mori H., Kung M.-P., Kung H.F., Saji H., Nakayama M. (2006). Novel Benzofuran Derivatives for PET Imaging of β-Amyloid Plaques in Alzheimer’s Disease Brains. J. Med. Chem..

[158] Kinney J.W., Bemiller S.M., Murtishaw A.S., Leisgang A.M., Salazar A.M., Lamb B.T. (2018). Inflammation as a central mechanism in Alzheimer’s disease. Alzheimer’s Dement. Transl. Res. Clin. Interv..

[159] Dokmeci D. (2004). Ibuprofen and Alzheimer’s disease. Folia Med..

[160] Lim G.P., Yang F., Chu T., Chen P., Beech W., Teter B., Tran T., Ubeda O., Ashe K.H., Frautschy S.A. (2000). Ibuprofen Suppresses Plaque Pathology and Inflammation in a Mouse Model for Alzheimer’s Disease. J. Neurosci..

[161] Weggen S., Eriksen J.L., Das P., Sagi S.A., Wang R., Pietrzik C.U., Findlay K.A., Smith T.E., Murphy M.P., Bulter T. (2001). A subset of NSAIDs lower amyloidogenic Aβ42 independently of cyclooxygenase activity. Nature.

[162] Wilkinson B.L., Cramer P.E., Varvel N.H., Reed-Geaghan E., Jiang Q., Szabo A., Herrup K., Lamb B.T., Landreth G.E. (2012). Ibuprofen attenuates oxidative damage through NOX2 inhibition in Alzheimer’s disease. Neurobiol. Aging.

[163] Zhang Y., Pike A. (2021). Pyridones in drug discovery: Recent advances. Bioorg. Med. Chem. Lett..

[164] Carlier P.R., Du D.M., Han Y., Liu J., Pang Y.P. (1999). Potent, easily synthesized huperzine A-tacrine hybrid acetylcholinesterase inhibitors. Bioorg. Med. Chem. Lett..

[165] Camps P., El Achab R., Görbig D.M., Morral J., Muñoz-Torrero D., Badia A., Baños J.E., Vivas N.M., Barril X., Orozco M. (1999). Synthesis, in Vitro Pharmacology, and Molecular Modeling of Very Potent Tacrine−Huperzine A Hybrids as Acetylcholinesterase Inhibitors of Potential Interest for the Treatment of Alzheimer’s Disease. J. Med. Chem..

[166] Li W., Kan K., Carlier P., Pang Y., Han Y. (2007). East Meets West in the Search for Alzheimers Therapeutics–Novel Dimeric Inhibitors from Tacrine and Huperzine A. Curr. Alzheimer Res..

[167] Mak S., Li W., Fu H., Luo J., Cui W., Hu S., Pang Y., Carlier P.R., Tsim K.W., Pi R. (2021). Promising tacrine/huperzine A-based dimeric acetylcholinesterase inhibitors for neurodegenerative disorders: From relieving symptoms to modifying diseases through multitarget. J. Neurochem..

[168] Nagahara A.H., Merrill D.A., Coppola G., Tsukada S., Schroeder B.E., Shaked G.M., Wang L., Blesch A., Kim A., Conner J.M. (2009). Neuroprotective effects of brain-derived neurotrophic factor in rodent and primate models of Alzheimer’s disease. Nat. Med..

[169] Giuffrida M.L., Copani A., Rizzarelli E. (2018). A promising connection between BDNF and Alzheimer’s disease. Aging.

[170] Knowles J. (2006). Donepezil in Alzheimer’s disease: An evidence-based review of its impact on clinical and economic outcomes. Core Evid..

[171] Alonso D., Dorronsoro I., Rubio L., Muñoz P., García-Palomero E., Del Monte M., Bidon-Chanal A., Orozco M., Luque F.J., Castro A. (2005). Donepezil–tacrine hybrid related derivatives as new dual binding site inhibitors of AChE. Bioorg. Med. Chem..

[172] Rose T.E., Morisseau C., Liu J.-Y., Inceoglu B., Jones P.D., Sanborn J.R., Hammock B.D. (2010). 1-Aryl-3-(1-acylpiperidin-4-yl)urea Inhibitors of Human and Murine Soluble Epoxide Hydrolase: Structure−Activity Relationships, Pharmacokinetics, and Reduction of Inflammatory Pain. J. Med. Chem..

[173] Jonnalagadda D., Wan D., Chun J., Hammock B.D., Kihara Y. (2021). A Soluble Epoxide Hydrolase Inhibitor, 1-trifluoromethoxyphenyl-3-(1-propionylpiperidin-4-yl) Urea, Ameliorates Experimental Autoimmune Encephalomyelitis. Int. J. Mol. Sci..

[174] Camps P., El Achab R., Morral J., Muñoz-Torrero D., Badia A., Baños J.E., Vivas N.M., Barril X., Orozco M., Luque F.J. (2000). New Tacrine−Huperzine A Hybrids (Huprines): Highly Potent Tight-Binding Acetylcholinesterase Inhibitors of Interest for the Treatment of Alzheimer’s Disease. J. Med. Chem..

[175] Chang J., Wang Q., Li Y. (2009). Synthesis and Biological Activity of Wuweizisu C and Analogs. Curr. Top. Med. Chem..

[176] Li Y., Li Y. (2001). Effect of dimethyl diphenyl bicarboxylate (DDB) on 9-amino-1,2,3,4-tetrahydroacridine-induced hepatotoxicity in mice. Yao Xue Xue Bao.

[177] Xu K., Dai X.-L., Huang H.-C., Jiang Z.-F. (2011). Targeting HDACs: A Promising Therapy for Alzheimer’s Disease. Oxid. Med. Cell. Longev..

[178] Xie R., Li Y., Tang P., Yuan Q. (2018). Design, synthesis and biological evaluation of novel 2-aminobenzamides containing dithiocarbamate moiety as histone deacetylase inhibitors and potent antitumor agents. Eur. J. Med. Chem..

[179] Jeong W.-H., Kim W.-I., Lee J.-W., Park H.-K., Song M.-K., Choi I.-S., Han J.-Y. (2021). Modulation of Long-Term Potentiation by Gamma Frequency Transcranial Alternating Current Stimulation in Transgenic Mouse Models of Alzheimer’s Disease. Brain Sci..

[180] Yang Y.-J., Wu P.-F., Long L.-H., Yu D.-F., Wu W.-N., Hu Z.-L., Fu H., Xie N., Jin Y., Ni L. (2010). Reversal of aging-associated hippocampal synaptic plasticity deficits by reductants via regulation of thiol redox and NMDA receptor function. Aging Cell..

[181] Eto K., Asada T., Arima K., Makifuchi T., Kimura H. (2002). Brain hydrogen sulfide is severely decreased in Alzheimer’s disease. Biochem. Biophys. Res. Commun..

[182] Giuliani D., Ottani A., Zaffe D., Galantucci M., Strinati F., Lodi R., Guarini S. (2013). Hydrogen sulfide slows down progression of experimental Alzheimer’s disease by targeting multiple pathophysiological mechanisms. Neurobiol. Learn. Mem..

[183] Chu Q.-J., He L., Zhang W., Liu C.-L., Ai Y.-Q., Zhang Q. (2013). Hydrogen sulfide attenuates surgical trauma-induced inflammatory response and cognitive deficits in mice. J. Surg. Res..

[184] Macdonald I.R., Martin E., Rosenberry T.L., Darvesh S. (2012). Probing the Peripheral Site of Human Butyrylcholinesterase. Biochemistry.

[185] Lopes J.P.B., Silva L., Ceschi M.A., Lüdtke D.S., Zimmer A.R., Ruaro T.C., Dantas R.F., de Salles C.M.C., Silva F.P., Senger M.R. (2019). Synthesis of new lophine–carbohydrate hybrids as cholinesterase inhibitors: Cytotoxicity evaluation and molecular modeling. MedChemComm.

[186] Sobrado M., López M., Carceller F., García A., Roda J. (2003). Combined nimodipine and citicoline reduce infarct size, attenuate apoptosis and increase bcl-2 expression after focal cerebral ischemia. Neuroscience.

[187] Shi J.-Q., Wang B.-R., Tian Y.-Y., Xu J., Gao L., Zhao S.-L., Jiang T., Xie H.-G., Zhang Y.-D. (2013). Antiepileptics Topiramate and Levetiracetam Alleviate Behavioral Deficits and Reduce Neuropathology in APPswe/PS1dE9 Transgenic Mice. CNS Neurosci. Ther..

[188] Camps P., Cusack B., Mallender W.D., El Achab R.E., Morral J., Muñoz-Torrero D., Rosenberry T.L. (2000). Huprine X is a novel high-affinity inhibitor of acetylcholinesterase that is of interest for treatment of Alzheimer’s disease. Mol. Pharmacol..

[189] Schneider L.S., Geffen Y., Rabinowitz J., Thomas R.G., Schmidt R., Ropele S., Weinstock M. (2019). Low-dose ladostigil for mild cognitive impairment. Neurology.

[190] Bender A.M., Jones C.K., Lindsley C.W. (2017). Classics in Chemical Neuroscience: Xanomeline. ACS Chem. Neurosci..

[191] Yeatman H.R., Lane J.R., Choy K.H.C., Lambert N.A., Sexton P.M., Christopoulos A., Canals M. (2014). Allosteric Modulation of M1 Muscarinic Acetylcholine Receptor Internalization and Subcellular Trafficking. J. Biol. Chem..

[192] Shirey J.K., Brady A.E., Jones P.J., Davis A.A., Bridges T.M., Kennedy J.P., Jadhav S.B., Menon U.N., Xiang Z., Watson M.L. (2009). A Selective Allosteric Potentiator of the M1 Muscarinic Acetylcholine Receptor Increases Activity of Medial Prefrontal Cortical Neurons and Restores Impairments in Reversal Learning. J. Neurosci..

[193] Xin R., Chen Z., Fu J., Shen F., Zhu Q., Huang F. (2020). Xanomeline Protects Cortical Cells From Oxygen-Glucose Deprivation via Inhibiting Oxidative Stress and Apoptosis. Front. Physiol..

[194] Micale V., Drago F., Noerregaard P.K., Elling C.E., Wotjak C.T. (2019). The Cannabinoid CB1 Antagonist TM38837 With Limited Penetrance to the Brain Shows Reduced Fear-Promoting Effects in Mice. Front. Pharmacol..

[195] Wise L.E., Iredale P.A., Stokes R.J., Lichtman A.H. (2007). Combination of Rimonabant and Donepezil Prolongs Spatial Memory Duration. Neuropsychopharmacology.

[196] Torres E., Duque M.D., López-Querol M., Taylor M.C., Naesens L., Ma C., Pinto L.H., Sureda F.X., Kelly J.M., Vázquez S. (2012). Synthesis of benzopolycyclic cage amines: NMDA receptor antagonist, trypanocidal and antiviral activities. Bioorg. Med. Chem..

[197] Cristóvão J.S., Gomes C.M. (2019). S100 Proteins in Alzheimer’s Disease. Front. Neurosci..

[198] Cai Z. (2014). Monoamine oxidase inhibitors: Promising therapeutic agents for Alzheimer’s disease (Review). Mol. Med. Rep..

[199] Shahid Nadeem M., Azam Khan J., Kazmi I., Rashid U. (2022). Design, Synthesis, and Bioevaluation of Indole Core Containing 2-Arylidine Derivatives of Thiazolopyrimidine as Multitarget Inhibitors of Cholinesterases and Monoamine Oxidase A/B for the Treatment of Alzheimer Disease. ACS Omega.

[200] Sterling J., Herzig Y., Goren T., Finkelstein N., Lerner D., Goldenberg W., Miskolczi I., Molnar S., Rantal F., Tamas T. (2002). Novel dual inhibitors of AChE and MAO derived from hydroxy aminoindan and phenethylamine as potential treatment for Alzheimer’s disease. J. Med. Chem..

[201] Fowler J.S., Logan J., Volkow N.D., Shumay E., McCall-Perez F., Jayne M., Wang G.-J., Alexoff D.L., Apelskog-Torres K., Hubbard B. (2015). Evidence that Formulations of the Selective MAO-B Inhibitor, Selegiline, which Bypass First-Pass Metabolism, also Inhibit MAO-A in the Human Brain. Neuropsychopharmacology.

[202] Piazzi L., Rampa A., Bisi A., Gobbi S., Belluti F., Cavalli A., Bartolini M., Andrisano V., Valenti P., Recanatini M. (2003). 3-(4-{[Benzyl(methyl)amino]methyl}phenyl)-6,7-dimethoxy-2 H -2-chromenone (AP2238) Inhibits Both Acetylcholinesterase and Acetylcholinesterase-Induced β-Amyloid Aggregation: A Dual Function Lead for Alzheimer’s Disease Therapy. J. Med. Chem..

[203] Chimenti F., Secci D., Bolasco A., Chimenti P., Bizzarri B., Granese A., Carradori S., Yáñez, M., Orallo F., Ortuso F. (2009). Synthesis, Molecular Modeling, and Selective Inhibitory Activity against Human Monoamine Oxidases of 3-Carboxamido-7-Substituted Coumarins. J. Med. Chem..

