# Supplementary material for: Tacrine-Based Hybrids: Past, Present, and Future
Source: Int J Mol Sci. 2023 Jan 15;24(2):1717. doi: 10.3390/ijms24021717 (PMC9863713; doi:10.3390/ijms24021717)
Supplement: Supplementary file 1 [file ijms-24-01717-s001.zip › ijms-2079161-supplementary.pdf]

Table S1. Tacrine – based hybrids with various biological activities, published in 2006 - 2022.

	Tacrin-like moiety	Second moiety	AChE/BChHE, IC_{50}	Biological action	Ref
		**Tacrine – Melatonine hybrids**			
**1 - 13**	Tacrine<br>6-Cl-THA	Melatonin	Human AChE/BChE<br>0.008 / 7.8 nM (**7**)	Antioxidant activity	[39]
**1 - 23**	Tacrine<br>6-Cl-THA	Melatonin	Human AChE/BChE<br>0.008 / 7.8 nM (**7**)<br>0.725 / 175 nM (**3**)<br>0.7 / 250 nM (**6**)	Inhibition of Aβ self-aggregation<br>Neuroprotection from Aβ and damage caused by mitochondrial free radicals – incuced oxidative stress	[40]
**24 - 31**	Tacrine<br>7-MEOTA	Melatonin + Ferulic acid	AChE from electric eel, BChE from equine serum<br>3.62 / 1.25 nM (**28**)<br>8.37 / 2.91 nM (**26**)<br>Human AChE/ BuChE<br>1290 / 234 nM (**26**)	Antioxidant activity<br>Neuroprotection	[41]
		**Another acrine hybrids with antioxidant activity**			
**32 - 48**	Tacrine<br>6-Cl-THA	8-Hydroxyquinoline	AChE from bovine, BChE from horse serum<br>20 / 5 nM (**35**)<br>75 / 2 nM (**40**)<br>85 / 6.5 nM (**45**)<br>Human AChE/BChE<br>5.5 / 20 nM (**35**)	Inhibition of Aβ aggregation, antioxidant activity, metal-chelating properties	[42]
**49 - 66**	Tacrine	Substituted benzene or pyridine moieties	AChE from electric eel, BChE from equine serum<br>4.55 / 3.41 nM (**58**)	Antioxidant activity<br>Antiaggregation properties	[43, 44]
**67 - 94**	Tacrine<br>6-Cl-THA	4-oxo-4H-chromene	AChE from bovine, BChE from horse serum<br>75 / 0.18 nM (**70**)<br>0.035 / 5.0 nM (**88**)<br>0.775 / 0.038 nM (**74**)<br><br>Human AChE/BChE<br>0.035 / 5.0 nM (**88**)	Antioxidant activity<br>Human BACE-1 inhibition	[45]
**95 - 100**	Tacrine<br>6-Cl-THA	Caffeic acid	AChE from electric eel, BChE from horse serum<br>0.3 / 29.5 μM (**99**)	Antioxidant activity<br>Antiaggregation properties<br>Neuroprotection<br>Metal- chelating properties	[46]
**101**	Tacrine	Silibinin	AChE from electric eel, BChE from equine serum<br>53.9 / 49.7 nM (**101**)	Low hepatotoxicity	[47, 48]
**102 - 110**	Tacrine	Ebselen	AChE from electric eel, BChE from equine serum<br>6.32 / 4.37 nM (**106**)<br>2.55 / 2.80 nM (**110**)	Antioxidant activity	[49]

**111 - 127**	Tacrine	b-Carbolines (pyrido[3,4 -b]indole)	AChE from electric eel, BChE from equine serum<br>21.6 / 39.8 nM (**122**)<br><br>Human AChE<br>63.2 ± 2.5 nM (**122**)	Antioxidant activity<br>Metal chelating properties<br>Neuroprotection<br>Antiaggregation properties	[50]
**128 - 148**	Tacrine<br>6-Cl-THA<br>7-MEOTA	Trolox	Human AChE/BChE<br>0.08 / 0.54 μM (**148**)	Antioxidant activity	[51]
**149 - 153**	Tacrine	N,N-dimethylated flavonoids	AChE from electric eel, BChE from equine serum<br>59.61 / 46.16 nM (**152**)<br>94.56 / 24.67 nM (**153**)	Antioxidant activity<br>Antiaggregation properties<br>Neuroprotection	[52]
**154 - 156**	Tacrine	Hydroxybenzoyl-pyridone	AChE from electric eel<br>0.57±0.05 μm (**156**)	Antioxidant activity<br>Metal-chelating properties	[53]
**157 - 191**	Tacrine	Phenolic acids Ligustrazine	AChE from electric eel, BChE from equine serum<br>3.9 / 24.3 nM (**165**)<br>2.6 / 28.6 nM (**175**)<br>Human AChE/BChE<br>65.2 / 48.8 nM (**165**)	Antioxidant activity<br>Antiaggregation properties<br>Neuroprotection<br>Low hepatotoxicity	[54, 55]
**192 - 210**	Tacrine<br>6-Cl-THA	Antioxidant CR- 6	Human AChE/BChE<br>0.44 / 17.4 nM (**207**)<br>0.12 / 13.4 nM (**208**)<br>0.27 /18.3 nM (**209**)	Antioxidant activity<br>Antiaggregation properties<br>Neuroprotection	[56]
**211 - 240**	Tacrine	Chalkone	BChE from equine serum<br>0.372 / 5.328 μM (**225**)<br>0.801 μM/- (**226**)<br>0.327 μM /- (**216**)	Neuroprotection	[57]
**241 - 249**	Huprine Y	Capsaicin	Human AChE/BChE<br>0.77 / 30.6 nM (**243**)<br>1.06 / 7.3 nM (**249**)	BACE-1 inhibition<br>Antioxidant activity	[58]
			**Tacrine - Ferulic acid hybrids**		
**250 - 254**	Tacrine	Ferulic acid	AChE from electric eel, BChE from equine serum<br>7.6 / 5.9 nM (**252**)<br>4.4 / 6.7 nM (**253**)	Antioxidant activity	[59, 60]
**255**	Tacrine	Ferulic acid	-	Antiaggregation properties<br>Antioxidant activity	[61]
**256 - 276**	Tacrine	Ferulic acid + NO-donors	AChE from electric eel, BChE from equine serum<br>10.9 / 17.1 nM (**262**)	Vascular relaxation (NO - donating properties)	[62]
**277 - 281**	Tacrine	Ferulic acid	52.7 / 215.4 nM (**279**)<br>61.7 / 109.6 nM (**280**)	Neuroprotection<br>Metal-chelating properties	[63]
**282 - 294**	Tacrine	Ferulic acid	AChE from electric eel, BChE from horse serum<br>49.5 / 69.4 nM (**285**)<br>37.0 / 101.4 nM (**288**)	Antiaggregation properties	[64]
			**Tacrine hybrids with NO-donating molecules**		

**295 - 309**	Tacrine	NO-donating groups	AChE from electric eel, BChE from equine serum<br>28.2 / 13.5 nM (**295**)<br>5.6 / 9.9 nM (**303**)<br>226.0 / 7.3 nM (**308**)	Vessel relaxant activity<br>Hepatoprotective effects	[65]
**310 - 317**	Tacrine	NO-donating groups	AChE from electric eel, BChE from equine serum<br>9.1 / 7.3 nM (**310**)<br>7.7 / 17.0 nM (**314**)	-	[66]
			**Tacrine – phenotiazine hybrids**		
**318 - 320**	Tacrine	Phenothiazine	AChE from rat brain homogenate 89 nM / - (**318**)	Reduce P-Tau accumulation<br>Binding with Aβ fibrils	[67]
**321 - 356**	Tacrine<br>6-Cl-THA<br>7-MEOTA	Phenothiazine	Human AChE/BChE<br>2040 / 15 nM (**321**)<br>8 / 190 nM (**332**)	Inhibition of Self-Induced $A\beta_{1-42}$ Aggregation<br>Inhibition of τ(306–336) peptide aggregation	[68]
			**Tacrine – benzotiazole/benzofuran hybrids**		
**357 - 364**	Tacrine	Benzotiazole<br>Benzofurane	AChE from electric eel, BChE from equine serum<br>0.017 / 0.122 μM (**359**)	Antiaggegating properties	[69, 70]
**365 - 369**	Tacrine	Benzotiazole	AChE from electric eel<br>0.34 / - μM (**365**)	Antiaggegating properties (**366**)<br>*Ex vivo* imrovement cell viability in cells treated with $A\beta_{42}$ peptide (**368, 369**)	[70]
**370 - 395**	Tacrine	Benzofuran	Human AChE/BChE<br>0.86 / 2.18 nM (**386**)	Antiaggegating properties<br>Inhibition of hBACE-1 activity	[71]
**396 - 401**	Tacrine<br>6-Cl-THA	Benzotiazole	AChE from electric eel<br>0.06 μM (**397**)	Antiaggegating properties<br>Neuroprotective effects	[72]
**402 - 413**	Tacrine<br>6-Cl-THA	Benzofuran	AChE from Electric eel<br>0.12 μM (**404**)<br>0.13 μM (**408**)<br>0.13 μM (**412**)	Antiaggegating properties<br>Neuroprotection	[73]
**414 - 438**	Tacrine<br>6-Cl-THA<br>7-MEOTA	Benzotiazole	Human AChE/BChE<br>1727 / 8 nM (**422**)<br>4 / 799 nM (**428**)<br>55 / 41 nM (**429**)<br>18 / 4657 nM (**436**)	Antiaggregation properties<br>Low hepatotoxicity	[74]
			**Tacrine hybrids with NSAIDS**		
**439 - 443**	Tacrine	Flurbiprofen	AChE from electric eel, BChE from equine serum<br>19.3 / 3.7 nM (**442**)<br>34.5 / 2.1 nM (**443**)	Inhibition of of amyloid-b formation	[75]
**444 - 455**	Tacrine	Flurbiprofen + NO-donors	AChE from electric eel, BChE from equine serum<br>4309.5 / 7.6 nM (**447**)<br>1456.4 / 3.9 nM (**455**)	Vasorelaxation effect	[76]
**456 - 463**	Tacrine	Flurbiprofen + NO-donors	AChE from electric eel, BChE from equine serum	Inhibition of of amyloid-b formation<br>Vascular relaxation	[77]

			9.1 / 2.5 nM (**456**)<br>12.5 / 1.0 nM (**460**)		
**464 - 471**	Tacrine	Indometacine	AChE from electric eel, BChE from equine serum<br>62 / 30 nM (**467**)<br>99 / 6 nM (**469**)<br>10 / 57 nM (**471**)	Antioxidant activity<br>Low hepatotoxicity	[78]
**472 - 475**	Tacrine	Ibuprofen	AChE from electric eel<br>47.25 nM / - (**475**)<br>39.16 nM (**475**, in the presence of H_2O_2)	Neuron-protective effects in the presence of H_2O_2.<br>H_2O_2 – responsible drug release<br>Inhibiting of proinflammatory cytokines (TNF-α and IL- 1β)<br>Regulating apoptosis related proteins (Bax, Bcl-2 and cleaved caspase- 3)	[79]
		**Tacrine-Hupyridone hybrids**			
**476**	Tacrine	Hupyridone	AChE from rat serum, BChE rat cortex homogenate<br>8.8 / 81.5 nM (**476**)	Low hepatotoxicity	[80 - 82]
		**Tacrine – Donepezil hybrids**			
**477 - 486**	Tacrine<br>6-Cl-THA	Donepezil	AChE/AChE/BChE<br>Bovine/human/human<br>0.09 / 0.27 / 66.3 nM (**480**)	Antiaggregating properties	[83, 84]
		**Tacrine-TPPU hybrids**			
**487 - 490**	6-Cl-THA<br>Huprine	TPPU, a epoxide hydrolase inhibitor	Human AChE/BChE<br>12.9 / 179 nM (**489**)	-	[85]
		**Tacrine - Huprine hybrids**			
**491 - 502**	Tacrine	Huprine	Human AChE/BChE<br>0.89/24.6 nM (**491**)<br>0.42/44.2 nM (**493**)<br>0.74/75.2 nM (**497**)<br>0.31/51.3 nM (**499**)	Antiaggregating properties	[86]
		**Tacrine - Bifendate hybrids**			
**503 – 507**	Tacrine	Bifendate	AChE from electric eel<br>BChE from equine serum<br>615.51 / 4.02 nM (**504**)<br>27.32 / 8.63 nM (**506**)<br><br>hAChE/hBChE (for 100 nM)<br>73.25 / 3.1% (**504**)<br>60.44 / 69.67% (**506**)	Antiaggreating properties<br>Low hepatotoxicity	[87]
		**Tacrine hybrids with HDAC inhibitors**			
**508- 535**	Tacrine<br>6-Cl-THA	Hydroxamates, HDAC inhibitors	AChE from electric eel, BChE from equine serum<br>13.00 / 0.58 nM (**512**)<br>0.12 / 361 nM (**517**)<br>0.26 / 140.06 nM (**535**)	Antioxidant activity<br>Metal – chelating properties<br>Inhibition ob Aβ aggregation<br>Dissolving of pre-formed Aβ aggregates	[88]
		**Tactine hybrids with thio derivatives**			

**536 - 541**	Tacrine<br>6-Cl-THA	Thiol group	AChE from electric eel, BChE from equine serum<br>7.37 / 7.04 (pIC_{50}) (**540**)	LTP enhancement<br>Neuroprotective activity<br>Low hepatotoxicity	[89]
**542-553**	Tacrine<br>6-Cl-THA	S-allylcysteine<br>S-propargylcysteine	AChE from Torpedo californica<br>0.30 μm (**545**)	Neuroprotection	[90]
**554**	Tacrine	H_2S donor	-	AChE inhibition in the serum and hippocampus of AD mice<br>Antiinflammatory activity	[91]
		**Tacrine hybrids with fluorescent probes**			
**555 - 564**	6-Cl-THA	Phenylpyrano[3,2 -c]quinoline	*AChE from bovine<br>human AChE/BChE<br>10.4* / 7.03 / 331 nM (**557**)	Inhibitors of $A\beta_{1-42}$ self-aggregation.<br>BACE-1 inhibition (681)	[92]
**565 - 568**	6-Cl-THA	Tetrahydrobenzo[h][1,6]naphthyridine	Human AChE/BChE<br>0.006 / 120 nM (**565**)	Antiaggregating properties	[93]
**569-591**	Tacrine	Lophine	AChE from mice brain<br>Human BChE<br>5.87 / 108.97 nM (**570**)<br>n.a. / 7.10 nM (**581**)	-	[94]
		**Tacrine hybrids with Ca^{2+} channel blocker**			
**592 - 600**	Tacrine	Nimodipine	*AChE from electric eel<br>Human AChE/BChE<br>*45 / 105/ 100000 nM (**600**)	Block of Ca^{2+} channels<br>Neuroprotection	[95, 96]
**601 - 605**	Tacrine	Nimodipine	AChE from electric eel, *Human AChE<br>45 / 105* (**S-600**)	Ca^{2+} channel blockade<br>Inhibition of AChE-induced $A\beta_{40}$ aggregation	[97]
**606**	Tacrine	Dihydropyridine	-	Reduction of phosphorylated tau levels,<br>inhibition of the generation and release of Aβ in cells	[98]
**607-618**	Tacrine	Dihydropyirimidine-thiones	Human AChE/BChE<br>0.0373 / 1.27 μm (**617**)<br>5.28 / 0.372 μm (**616**)	Ca^{2+} channel blockade<br>Weak inhibition of $A\beta_{40}$ self-aggregation	[99]
**619 - 622**	Tacrine<br>6-Cl-THA<br>Huprine	Levetiracetam	Human AChE/BChE<br>3.1 / 135 nM (**±620**)<br>4.2 / 232 nM (**SSR-621**)	Anti-aggregating activity ($A\beta_{42}$ and tau)<br>Reduction of AChE activity in brain (*ex vivo* test)<br>Reduction cortical amyloid burden and neuroinflammation around Aβ plaques were significantly reduced (*ex vivo* test after therapy)	[100]
		**Tacrine hybrids with serotonin receptors antagonists**			
**623 - 628**	Tacrine	1 -(phenylsulfonyl)- 4 -(piperazin- 1 -yl)- 1H-indole	AChE from electric eel, BChE from equine serum; human *AChE/**BuChE;<br>7.1 / 12.4 / 1.3* nm (**628**)<br>45.9 / 8.2 / 12.9*/ 29.7** nm (**626**)	$5HT_6$ in agonists	[101]

**629-638**	Tacrine	1 -(3 -(benzyloxy)- 2 -methylphenyl)piperazine<br>1 -benzyl- 4 -(piperazin- 1 -yl)- 1H-indole	AChE from electric eel, *human AChE, BChE from equine serum<br>0.005 / 0.003 / 0.024 μM (**634**)	Low hepatotoxicity<br>Antiaggregating properties	[102]
**639 - 668**	Tacrine	Vilazodone	AChE from rat cortex, BChE from rat serum<br>3.319 / 15.79 μm (**643**)	Low hepatotoxicity<br>Low cardiotoxocity<br>5 -HT1A agonist<br>5 -HT reuptake inhibitor	[103]
**Tacrine hybrids with modulator of muscarinic receptors**					
**669 - 674**	Tacrine<br>6-Cl-THA	Gallamine	*AChE from electric eel<br>Human AChE/BChE<br>0.467*/ 7.61/ 1.50 nM (**669**)<br>28.9*/ 23.2 / 2.40 nM (**673**)	Allosteric M2 receptor modulation	[104]
**675 - 690**	Tacrine	Xanomeline	AChE from electric eel, BChE from equine serum<br>8.18 / 8.29 (pIC_{50}) (**687**)<br>8.21 / 8.23 (pIC50) (**690**)	Enhanced M1 allosteric affinity	[105]
**691 - 711**	Tacrine<br>6-Cl-THA<br>7-MEOTA	Benzylquinolone carboxylic acid (BQCA)	Human AChE/BChE<br>1.5 / 49.3 μM (**696**)<br>0.13 / 0.67 μM (**699**)<br>42 / 372 nM (**706**)	Muscarinic receptors antagonists	[106]
**712 - 717**	Tacrine	Xanomeline	AChE from electric eel<br>pIC_{50} = 9.55 (**715**)	-	[107]
**Tacrine hybrids with cannabinoid CB1 receptor antagonists**					
**718 - 721**	Tacrine	Cannabinoid CB1 receptor antagonists	Human AChE<br>pIC_{50} = 6.5 (**720**)	CB1 receptor antagonists	[108]
**Tacrine hybrids with modulator of NMDA receptors**					
**722 - 728**	7 -MEOTA	Adamantylamine	Human AChE/BChE<br>0.47 / 0.11 μM (**725**)	-	[109]
**729 - 732**	6-Cl-THA	Benzohomoadamantane	Human AChE/BChE<br>0.3 / 0.5 nM (**731**)	NMDA inhibition	[110]
**Tacrine hybrids with modulator of opioid receptors**					
**733 - 747**	Tacrine<br>6-Cl-THA	Tianeptine	AChE from rat brain homogenate<br>Human BChE<br>6.79 / 52.44 nM (**736**)<br>156.19 / 3.59 nM (**737**)	Reduction of the *in vitro* basal secretion of S100B	[111]
**Tacrine hybrids with MAO inhubitors**					
**748 - 760**	Tacrine<br>6-Cl-THA	Selegiline	AChE from electric eel, BChE from equine serum<br>22.6 / 9.37 nM (**754**)	MAO-A,<br>MAO-B inhibition	[112]
**761 - 780**	Tacrine	Coumarin	AChE from electric eel, BChE from equine serum<br>17.70 / 38.00 nM (**766**)<br>33.63 / 80.72 nM (**773**)	MAO-B selective inhibition<br>Low neurotoxocity	[113]
**Tacrine hybrids with natural products**					

**781 - 800**	Tacrine	Coumarin	AChE from electric eel, BChE from equine serum<br>0.092 / 0.234 μM (**786**)<br>0.398 / 0.099 μM (**790**)	Metal-chelating ability<br>Antiaggregating properties<br>Low neurotoxicity,	[114]
**801-805**	Tacrine	Coumarin	Human AChE/BChE<br>0.0154 / 0.328 μM (**803**)	-	[115]
**806 - 826**	Tacrine	Flavonoid	AChE from electric eel, BChE from equine serum<br>8.4 / 35.0 nM (**825**)<br>10.3 / 25.8 nM (**826**)	Metal-chelating ability<br>Antiaggregating properties<br>Low neurotoxicity	[116]
**827 - 834**	Huprine Y	Rhein	Human AChE/BChE<br>1.07 / 950 nM (**±827**)<br>1.52 / 1070 nM (**±828**)	Antiaggregating propertiesd<br>BACE-1 inhibition<br>Prevent the loss of synaptic proteins in hippocampal slices (*ex vivo*)	[117]
**835 - 837**	Tacrine	Carbazole	AChE from electric eel; human BChE<br>0.48 / 52.14 μM (**835**)	Neuroprotection from ROS-or Aβ-induced toxicity	[118]
**838 - 844**	6-Cl-THA	Scutellarin	Human AChE/BChE<br>1.63 / 1210 nM (**838**)<br>1.9 / 174 nM (**839**)	-	[119]
**845 – 852**	Tacrin<br>6-Cl-THA<br>7-MEOTA	Resveratrol	Human AChE/BChE<br>0.8 / n.a. μM (**845**)	Antiaggreating properties<br>Antiinflammatory activity	[120]
**853 – 861**	Tacrine	Carbohydrates	AChE from mice brain extract, BChE from mice blood serum<br>84.4 / 11.7 nM (**854**)<br>2.2 / 4.93 nM (**857**)	**-**	[121]
**862 - 882**	Tacrine<br>6-Cl-THA<br>7-MEOTA	Tryptophan	Human AChE/BChE<br>6.3 / 9.1 nM (**S-873**)	Inhibition of Aβ_{42}-self-aggregation	[122]
**883 - 889**	Tacrine	Indole	AChE from electric eel, BChE from equine serum<br>0.173 / 0.066 μM (**887**)	**-**	[123]
**890 - 902**	Tacrine<br>6-Cl-THA	Anacardic acid, cardanol, and cardol from cashew nutshell liquid	Human AChE/BChE<br>2.54 / 0.265 nM (**891**)<br>20.8 / 0.0352 nM (**890**)<br>17.0 / 0.177 nM (**897**)	Antiinflammatory activity<br>Neuroprotection (**890, 891**) at 0.01 μM<br>Low hepatotoxicity<br>Low neurotoxicity	[124]
		**Tacrine hybrids with organic scaffolds**			
**903 - 917**	Tacrin	Trimethoxybenzene	Human AChE/BChE<br>5.08 / 1.38 nM (**912**)<br>59.00 / 0.139 nM (**916**)	-	[125]
**918 - 921**	Tacrine	Tiofen-based photoswitcher	AChE from electric eel, BChE from equine serum<br>*Human AChE<br>4.3 / 5.9 / 49.6* nM (open) (**921**)<br>1.8 / 7.2 / 19.1* nM (closed) (**921**)	Inhibition of AChE-induced Aβ aggergation	[126]
**922 - 939**	Tacrine<br>6-Cl-THA<br>7-MEOTA	Quinone	Human AChE/BChE<br>1.93 / 256 nM (**926**)<br>0.72 / 542 nM (**930**)	Antiaggregating properties<br>Neuroprotection from ROS-or Aβ-induced toxicity	[127]

				Low hepatotoxicity	
**940 - 944**	Tacrine<br>6-Cl-THA	Propargylamine	AChE from electric eel, BChE from equine serum<br>11.2 / 83.5 nM (**941**)<br><br>Human AChE<br>9.4 ± 0.7 (**941**)	Low hepatotoxicity<br>Low neurotoxicity	[128]
**945 - 953**	7-MEOTA	p-Anisidine	Human AChE/BChE<br>1.36 / 10.2 µm (**951**)<br>1.35 / 10.9 µm (**955**)	-	[129]
**959 - 973**	Tacrine<br>6-Cl-THA	Triazole	AChE from electric eel, BChE from equine serum<br>2.00 / 0.055 µM (**968**)<br>0.521 / 1.853 µM (**970**)	-	[130]
**974 - 989**	Tacrine	Isatine	AChE from electric eel, BChE from equine serum<br>57.85 / 0.11 nM (**977**)<br>0.42 / 0.57 nM (**984**)<br>0.62 / 3.52 nM (**986**)	Inhibition of self-induced $A\beta_{1-40}$ aggregation<br>Metal-chelating properties	[131]
**990 - 1039**	Tacrine<br>6-Cl-THA<br>7-MEOTA	Pyrimidone	AChE from the cerebral cortex of a mouse 51.1 / - nM (**1035**)	GSK-3β inhibition<br>Regulation of the tau protein pathway in SH-SY5Y-derived neurons<br>Neuroprotection	[132]
**1040 - 1052**	Tacrine	Carbamate	Human AChE/BChE<br>22.15 / 16.96 nM (**1050**)	**-**	[133]
**1053 - 1066**	Tacrine	Phosphorus group	AChE from electric eel, BChE from equine serum<br>6.11 / 12.86 nM (**1055**)	Lower hepatotoxicity *in vitro*	[134]

Table S2. Summary of in vivo study of anti-AD properties of the hybrids.

Hybrid	Tacrin	Second moiety	Mode of injection, drug dose	*In vivo* effects	Ref.
**101**	Tacrine	Silibinin	i.p.<br>2 µmol/100 g	Low hepatotoxicity *in vivo*<br>Cognitive improvement in scopolamine-injected mice, similar to that of tacrine	[47, 48]
**165**	Tacrine	Phenolic acids<br>Ligustrazine	i.g.<br>1.27 mg/100<br>daily<br>4 weeks	Low hepatotoxicity *in vivo*<br>After 4 weeks of intragastric administration, cognitive function and synaptic plasticity were significantly improved and Aβ plaques were decreased in the APP/PS1 mice	[55]
**193, 197**	6-Cl-THA	Antioxidant CR-6	i.p.<br>10 mg/kg/day<br>4 weeks	Positive tendencies in improving cognition, oxidative stress, and amyloid pathology on wild-type and APP/PS1 mice	[56]
**216, 225, 226**	Tacrine	Chalkone	5 mg/kg/day<br>1 week	*In vivo* behavior studies showed a cognitive improvement in mice pretreated with scopolamine. Recovery of scopolamine-induced glutathione depletion, in-brain malondialdehyde (MDA) level decrease	[57]

**249**	Huprine Y	Capsaicin	i.p.<br>2 mg/kg<br>3 times/week<br>4 weeks	Significant enhance of learning and memory in 10-month-old APP/PS1 mice. Decrease of the $A\beta_{42}/A\beta_{40}$ ratio in hippocampus. Improvement in basal synaptic efficacy, and significant reduction of hippocampal oxidative stress and neuroinflammation	[58]
**254**	Tacrine	Ferulic acid	i.p.<br>1.22 mg/100g	No improvement in scopolamine-injected mice (compd. amplifies scopolamine action)	[60]
**255**	Tacrine	Ferulic acid	i.g.<br>2 mg/kg/day,<br>20 mg/kg/day<br>3 weeks	Cognitive impairment, increase of ChAT activity and decreases AChE activity in Aβ icv mice	[61]
**262**	Tacrine	Ferulic acid + NO-donors	i.d.<br>Behavioral. studies<br>1.978 μmol/100 g<br>Hepatotoxicity study<br>11.86 μmol/100 g	Cognitive improvement in scopolamine-injected mice<br>Low hepatotoxicity *in vivo*	[62]
**285, 288**	Tacrine	Ferulic acid	i.g.<br>30 mg/kg/day	Cognition improving in scopolamine-injected adult ICR mice<br>Low hepatotoxocity *in vivo*	[64]
**295, 303, 310**	Tacrine	NO-donating groups	i.p.<br>Hepatotoxicity study<br>5.93 μmol/ 100 g<br>Behavioral studies<br>1.978 μmol/100 g	Much less hepatotoxicity compared to tacrine (**295, 303**).<br>Cognition improving in scopolamine-injected mice	[66]
332	6-Cl-THA	Phenothiazine	i.p.<br>14 mg/kg	Non-toxic in doses 14 mg/kg<br>*In vivo* CNS availability, relatively low elimination from the brain tissue	[68]
**386**	Tacrine	Benzofuran	Oral intake<br>Behavioral studies<br>20 μmol/kg/day<br>10 days<br>Hepatotoxicity<br>equimolar to 30 mg/kg of tacrine	Low hepatotoxicity *in vivo*<br>Cognitive improvements in ICR mice	[71]
**436**	6-Cl-THA	Benzotiazole	i.p.<br>2 mg/kg/day<br>4 days	Mild cognitive improvements	[74]
**442, 456**	Tacrine	Flurbiprofen + NO-donors	i.d.<br>Behavioral studies<br>1.978 μmol/100 g<br>Hepatotoxicity<br>11.86 μmol/100 g	Cognition improving activity in ICR mice<br>Low hepatotoxicity *in vivo* (Hybrid **456** showed the lowest toxicity)	[77]
**475**	Tacrine	Ibuprofen	I.p.<br>0.9 mg/100g/day<br>4 weeks	Improving spatial memory of Aβ-induced AD model rats	[79]
**476**	Tacrine	Hupyridone	I.p.<br>0.36 μmol/kg<br>0.72 μmol/kg every third day, 28 weeks	Attenuates scopolamine-induced impairments of cognition in APP/PS1 mice.<br>At high concentrations (10 μM) did not induce obvious hepatotoxicity	[80]
**489**	6-Cl-THA	TPPU, an epoxide hydrolase inhibitor	Oral intake<br>2 mg/kg/day<br>4 weeks	Significant amelioration in short-term and long-term working memory of SAMP8 mice<br>Reduction of neuroinflammation<br>lack of neurotoxicity	[85]
**506**	Tacrine	Bifendate	Oral intake<br>Behavioral studies<br>20 μmol/kg/day<br>4 days<br>Hepatotoxicity studies<br>i.g.<br>106 mg/kg	Low hepatotoxicity *in vivo*<br>Ameliorated the cognition impairment in the scopolamine treated ICR mice (8–10 weeks old).	[87]
**536, 537**	Tacrine	Thiol group	i.c.v.	Enhance of the hippocampal LTP *in vivo*	[89]

**541**	6-Cl-THA		<u>Synaptic plasticity study</u><br>50 µM (541)<br><u>Hepatotoxicity</u><br>6 µmol/100 g (536 - 537)	Low hepatotoxicity *in vivo*	
**542**	Tacrine	H_2S donor	i.p.<br>15 mmol/kg/day<br>20 days	Improvment of cognitive and locomotor activity in AD mice (therapeutic effect of the lower dose was only partial)<br>Low hepatotoxocity *in vivo*	[90]
**598, 605**	Tacrine<br>6-Cl-THA	Huprine	i.p.<br>10 µmol/kg	*Ex vivo* inhibition of AChE from OF1 mice	[96],[97]
**619, 622**	Tacrine<br>Huprine	Levetiracetam	<u>Inhibition of AChE</u><br>i.p.<br>2.5 mg/kg (**619**)<br>5 mg/kg (**621**)<br><u>Efficacy Studies</u><br>i.p.<br>5 mg/kg/day<br>4 weeks	Inhibition of C57BL6J mouse brain AChE 10 min after administration<br>A reduction of number of epileptic seizures and reduction of Aβ burden in the cortex of APP/PS1 mice (**621**)<br>Cognitive improvement in transgenic 5-month-old APP/PS1 mice (**619, 621**).	[100]
**626**	Tacrine	1 -(phenylsulfonyl)-4 -(piperazin- 1 -yl)- 1H-indole	i.p.<br>0.3 - 10.0 mg/kg	Cognitive improvementt in scopolamine-injected mice. Dose-dependent reduction of scolopamine-induced hyperlocomotion	[101]
**643**	Tacrine	Vilazodone	Oral intake<br>30 mg/kg	Behaviour impaimeint in scopalomine-treated mice<br>Alleviation of the depressive symptom in tail suspension test	[103]
**696**	Tacrine	Xanomeline	I.p.<br>2 µmol/100 g	Significantly enhance scopolamine action	[106]
**831**	Huprine Y	Rhein	<u>Determination of Synaptic Protein Levels</u><br>i.v.<br>10 µM<br><u>Aβ Lowering Effect</u><br>i.p.<br>2 mg/kg<br>3 times per week for 4 weeks	*Ex vivo* reduction of Aβ levels of in hippocam (2-month-old C57bl6 mice)<br>An increase of APP levels in brain of 10 -month-old APP/PS1 mice	[117]
**835**	Tacrine	Carbazole	i.p.<br>25 µmol/kg<br>50 µmol/kg	Improve memory deficit in mice induced by scopolamine	[118]
**(S)-873**	6-Cl-THA	Tryptophan	<u>Behavioral studies</u><br>i.c.v<br>10 nM 100 nM<br><u>Toxicity study</u><br>i.p.<br>70 mg/kg	Pro-cognitive potential in a scopolamine-induced cognitive deficit rat model (9 -12 weeks old wistar rats)	[122]
**926, 930**	6-Cl-THA	Quinone	i.p.<br>10 µmol/kg<br>50 µmol/kg	*Ex vivo* dose-dependent inhibition of cholinesterase activity in telencephalon	[127]
**970**	Tacrine	Triazole	i.p.<br>5 mg/kg/day<br>10 mg/kg/day<br>20 mg/kg/day.<br>4 days	Memory improvement in scopolamine-induced impairment for all concentrations	[130]
**1017**<br>**1035**	Tacrine<br>6-Cl-THA	Pyrimidone	i.p.<br>15 mg/kg/day<br>15 days	Significant amelioration of memory and spatial behavior in Scop-injected mice (8–10 weeks old)<br>Hybrid was **1035** slightly better than **1017**	[132]
